# The chloroplast genome of *Amygdalus* L. (Rosaceae) reveals the phylogenetic relationship and divergence time

**DOI:** 10.1186/s12864-021-07968-6

**Published:** 2021-09-07

**Authors:** Zhongyu Du, Ke Lu, Kai Zhang, Yiming He, Haitao Wang, Guaiqiang Chai, Jianguo Shi, Yizhong Duan

**Affiliations:** 1 College of life science, Shaanxi Key Laboratory of Ecological Restoration in Northern Shaanxi Mining Area, Yulin University, Yulin, China; 2 School of Ecology and environment, Breeding Base for State Key Laboratory of Land Degradation and Ecological Restoration in Northwest China, Ministry of Education Key Laboratory for Restoration and Reconstruction of Degraded Ecosystems in Northwest China, Ningxia University, Yinchuan, China

**Keywords:** *Amygdalus* L., Complete chloroplast genome, Phylogenetic relationship, Divergence time estimation

## Abstract

**Background:**

Limited access to genetic information has greatly hindered our understanding of the molecular evolution, phylogeny, and differentiation time of subg. *Amygdalus*. This study reported complete chloroplast (cp) genome sequences of subg. *Amygdalus*, which further enriched the available valuable resources of complete cp genomes of higher plants and deepened our understanding of the divergence time and phylogenetic relationships of subg. *Amygdalus*.

**Results:**

The results showed that subg. *Amygdalus* species exhibited a tetrad structure with sizes ranging from 157,736 bp (*P. kansuensis*) to 158,971 bp (*P. davidiana*), a pair of inverted repeat regions (IRa/IRb) that ranged from 26,137–26,467 bp, a large single-copy region that ranged from 85,757–86,608 bp, and a small single-copy region that ranged from 19,020–19,133 bp. The average GC content of the complete cp genomes in the 12 species was 36.80%. We found that the structure of the subg. *Amygdalus* complete cp genomes was highly conserved, and the 12 subg. *Amygdalus* species had an *rps*19 pseudogene. There was not rearrangement of the complete cp genome in the 12 subg. *Amygdalus* species. All 12 subg. *Amygdalus* species clustered into one clade based on both Bayesian inference and maximum likelihood. The divergence time analyses based on the complete cp genome sequences showed that subg. *Amygdalus* species diverged approximately 15.65 Mya.

**Conclusion:**

Our results provide data on the genomic structure of subg. *Amygdalus* and elucidates their phylogenetic relationships and divergence time.

**Supplementary Information:**

The online version contains supplementary material available at 10.1186/s12864-021-07968-6.

## Introduction

*Amygdalus* L. (Rosaceae), a subgenus of the genus *Prunus* L. [1–3], is a small group within *Prunus* that includes approximately 24 species. Subg. *Amygdalus* species are mainly distributed in Iran and eastern Turkey, but a few are distributed in southeastern Europe, the Mediterranean region, Mongolia, and China [4, 5]. Subg. *Amygdalus* members are shrubs or small trees that mostly grow between 1000 and 2500 m above sea level in mountainous areas [5]. Many studies have elucidated various aspects of subg. *Amygdalus* members; for example, Maatallaha et al. [6] evaluated mineral nutrients, phenolic and volatile profiles, and antioxidant activities of peach cultivars and assessed their potential for use in cultivar improvement [5, 7]. Moreover, subg. *Amygdalus* species are valuable fruit trees and can be common ornamental plants, among which *P. persica* has over one thousand years of cultural history and is one of the five oldest cultivated fruit species with distinct advantages in the world [8].

In recent years, members of subg. *Amygdalus* have become important subjects of many studies [9–12]. For example, *P. dulcis* produces a lot of simple gum exudates that are obtained from its trunk, branches, and fruits [13]. *Prunus davidiana* var. *potaninii* Rehd., as an important rootstock of drupe fruit trees in northwestern China, is a wild relative of *P. davidiana* [14]. Fang et al. [15] constructed regression equations between the ages and base diameters of *P. mira* by data processing system, and concluded that the populations of *P. mira* in Linzhi are declining; this species plays an important role in the germplasm improvement of cultivated peach. *Prunus mira* was also used to facilitate vegetation and rootstock recovery to mitigate land degradation in many areas because of its high tolerance to drought, cold, and barren soil [16]. *Prunus kansuensis*, which has strong cold resistance and high drought tolerance, can be used as ornamental woody plants. However, the yields of cultivars have recently been seriously affected by diseases and insect pests [17, 18]. Yazbek and Oh [5] reconstructed and analyzed the phylogenetic relationships of subg. *Amygdalus* by DNA sequencing and morphology, and then evaluated the morphological characteristics used for the classification of subg. *Amygdalus*. Vafadar et al. [19] identified the pollen morphology of hybrids of subg. *Amygdalus*, and analyzed common pollen grain features. Several studies have conducted phylogenetic analysis on stone fruits based on internal transcribed spacer (ITS) technology using genetic distance thresholds [20–22]. However, a study also showed that accurate identification of species may be impacted by a single threshold [23]. DNA metabarcoding is limited by bias of polymerase chain reaction, resolution of barcoding, universality, and perfect degree of database [24]. The chloroplast (cp) genome of subg. *Amygdalus* species may be essential for illuminating the evolution of and distinguishing the subg. *Amygdalus* species. No studies have addressed phylogenetic relationships and estimated divergence time of subg. *Amygdalus*.

The cp is an important self-replicating organelle that plays a vital role in photosynthesis and energy transformation [25, 26]. Previous studies have shown that the cp genome, which is 115 kb–165 kb in sequence length, consists of a characteristic circular quadripartite structure that includes a large single-copy (LSC) region, a small single-copy (SSC) region, and two inverted repeat (IRa and IRb) regions [27]. Furthermore, compared with the nuclear genome, angiosperm cps are highly conserved in gene composition and genome structure [28], and the structure of plastids, which have unique advantages in phylogenetic reconstruction, is stable, usually uniparental, haploid, and non-recombinant. In this study, complete cp genome sequences of 12 subg. *Amygdalus* species were compared and analyzed to explore their sequence characteristics and structural differences; these complete cp genome sequences provide additional valuable cp genomic resources of subg. *Amygdalus*. The aims of the present study were to: (1) explore the complete cp genome sequence of 12 subg. *Amygdalus* species; (2) clarify the subg. *Amygdalus* relationships in genus *Prunus*; and (3) estimate the divergence times of subg. *Amygdalus*.

## Results

### Comparative analysis of cp genomes of subg. *Amygdalus* species

**Fig. 1:**
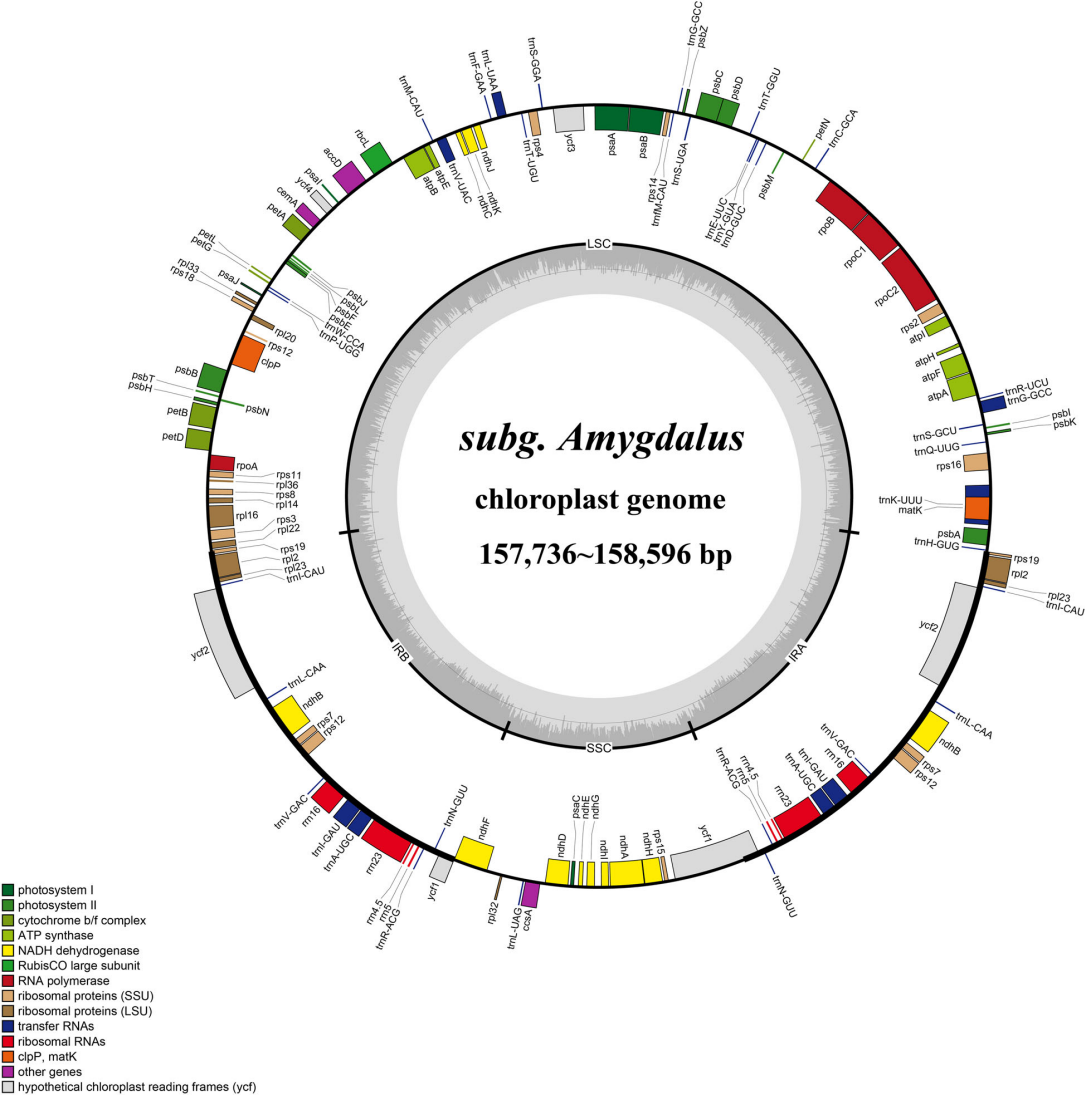
Complete chloroplast genome map of 12 subg. *Amygdalus* species

The complete cp genome sequence of 12 subg. *Amygdalus* species exhibited a circular DNA molecule with a typical quadripartite structure; they have a pair of inverted repeats regions (IRa and IRb), one LSC region, and one SSC region (Fig. 1, Table 1). The complete cp genome sequence of the 12 subg. *Amygdalus* species ranged from 157,736 bp (*P. kansuensis*) to 158,971 bp (*P. davidiana*) in length. The IRa/IRb regions ranged from 26,137–26,467 bp, the LSC region ranged from 85,757–86,608 bp, and the SSC region ranged from 19,020–19,133 bp (Fig. 1, Table 1). The average GC content of the complete cp genome in the 12 species was 36.80%, and the average GC content of the IR regions was 42.60% (Table 1). The cp genomes of the subg. *Amygdalus* species encoded a total of 131/133 genes, including 86 protein-coding genes (PCGs), 37/39 tRNA genes, and eight rRNA genes. In *P. tenella*, there were no *petB* and *ycf3* genes. In *P. davidiana* var. *potaninii* Rehd., *P. kansuensis*, *P. persica*, *P. dulcis*, *P. davidiana*, and *P. mongolica*, there were two *rps19* genes and the *ycf15* gene was lost. The *rps4* gene of *P. ferganensis*, *P. mira*, *P. tenella*, *P. pedunculata*, *P. tangutica*, and *P. triloba* were lost (Table 1, Table 2). There were 18 intron-containing genes; the *rps12*, *clpP*, and *ycf3* genes contained two introns, and the other genes had a single intron (Table 2).

**Table 1:** Comparison of complete chloroplast genome features of 12 subg. *Amygdalus* species

Species	Genome (bp)	LSC length (bp)	SSC length (bp)	IR length (bp)	Number of PCGs	Number of tRNAs genes (bp)	Number of rRNAs genes (bp)	GC content (%)	GC content in LSC (%)	GC content in SSC (%)	GC content in IR (%)	Accession number
*P. kansuensis*	157,736	85,757	19,133	26,380	86 (8)	37 (7)	8 (4)	36.8	34.6	30.3	42.6	NC023956
*P. persica*	157,790	85,882	19,060	26,467	86 (8)	37 (7)	8 (4)	36.8	34.6	30.4	42.6	HQ336405
*P. pedunculate*	157,851	86,052	19,029	26,385	86 (7)	37 (7)	8 (4)	36.8	34.6	30.4	42.6	MG602257
*P. davidiana*	158,971	86,607	19,027	26,381	86(7)	37(7)	8(4)	36.7	34.6	30.4	42.6	MK798145
*P. dulcis*	158,085	86,322	19,070	26,137	86(7)	37(7)	8(4)	36.8	34.6	30.5	42.7	MT019559
*P. tangutica*	158,166	86,146	19,040	26,386	86 (9)	37 (7)	8 (4)	36.8	34.6	30.5	42.6	MK780039
*P. mira*	158,198	86,198	19,032	26,380	86 (8)	37 (7)	8 (4)	36.8	34.6	30.3	42.5	MK798147
*P. davidiana* var. *potaninii* Rehd.	158,361	86,488	19,133	26,371	86 (9)	37 (7)	8 (4)	36.8	34.5	30.4	42.6	MT019558
*P. ferganensis*	158,365	86,471	19,008	26,386	86 (9)	37 (7)	8 (4)	36.8	34.7	30.5	42.6	MK798146
*P. mongolica*	158,039	86,173	19,084	26,391	86(7)	37(7)	8(4)	36.7	34.6	30.3	42.6	NC037849
*P. triloba*	158,455	86,422	19,031	26,317	86 (9)	37(7)	8(4)	36.8	34.6	30.5	42.7	MK790138
*P. tenella*	158,596	86,608	19,020	26,404	86 (8)	37 (7)	8 (4)	36.7	34.6	30.3	42.6	MK764428

**Table 2:** List of genes present of complete chloroplast genomes 12 subg. *Amygdalus* species

Category of Genes	Group of Gene	Name of Gene	Name of Gene	Name of Gene	Name of Gene	Name of Gene
Self-replication	Ribosomal RNA genes	rrn4.5^(× 2)^	rrn5^(× 2)^	rrn16^(× 2)^	rrn23^(× 2)^	
	Transfer RNA genes	trnA-UGC^*,(× 2)^	trnC-GCA	trnD-GUC	trnE-UUC	trnF-GAA
		trnfM-CAU	trnG-GCC*	trnG-UCC	trnH-GUG	trnI-CAU^(×2)^
		trnI-GAU^*,(× 2)^	trnK-UUU*	trnL-CAA^(× 2)^	trnL-UAA*	trnL-UAG
		trnM-CAU	trnN-GUU^(× 2)^	trnP-UGG	trnQ-UUG	trnR-ACG^(×2)^
		trnR-UCU	trnS-GCU	trnS-GGA	trnS-UGA	trnT-GGU
		trnT-UGU	trnV-GAC^(× 2)^	trnV-UAC^*^	trnW-CCA	trnY-GUA
	Small subunit of ribosome	rps2	rps3	rps4^(a,b,d,h,l)^	rps7^(×2)^	rps8
		rps11	rps12^**,(×2)^	rps14	rps15	rps16^*^
		rps18	rps19^1)^			
	Large subunit of ribosome	rpl2^*,(×2)^	rpl14	rpl16^*^	rpl20	rpl22
		rpl23^(×2)^	rpl32	rpl33	rpl36	
	DNA-dependent RNA polymerase	rpoA	rpoB	rpoC1^*^	rpoC2	
Genes for photosynthesis	Subunits of NADH-dehydrogenase	ndhA^*^	ndhB^*,(×2)^	ndhC	ndhD	ndhE
		ndhF	ndhG	ndhH	ndhI	ndhJ
		ndhK				
	Subunits of photosystem I	psaA	psaB	psaC	psaI	psaJ
	Subunits of photosystem II	psbA	psbB	psbC	psbD	psbE
		psbF	psbH	psbI	psbJ	psbK
		psbL	psbM	psbN	psbT	psbZ
	Subunits of cytochrome b/f complex	petA	petB^*^	petD^*^	petG	petL
		petN				
	Subunits of ATP synthase	atp A	atp B	atp E	atp F^*^	atp H
		atpI				
	Subunits of rubisco	rbcL				
Other genes	Maturase	matK				
	Protease	clpP^**^				
	Envelope membrane protein	cemA				
	Subunit of acetyl-CoA carboxylase	accD				
	C-type cytochrome synthesis gene	ccsA				
Genes of unknown function	Conserved open reading frames	ycf1^(×2)^	ycf2^(×2)^	ycf3^**(c)^	ycf4	ycf15^(×2)(e,f,g,i,j,k)^

^1)^ genes are two in *P. davidiana* var. *potaninii* Rehd., *P. kansuensis*, *P. persica*, *P. dulcis*, *P. davidiana* and *P. mongolica* and only one in *P. ferganensis*, *P. mira*, *P. tenella*, *P. pedunculata*, *P. tangutica*, *P. triloba*; ^a^ that does not have this gene in *P. ferganensis*; ^b^ that does not have this gene in *P. mira*; ^d^ that does not have this gene in *P. tangutica*; ^e^ that does not have this gene in *P. davidiana* var. *potaninii* Rehd.; ^f^ that does not have this gene in *P. kansuensis*; ^g^ that does not have this gene in *P. persica*; ^h^ that does not have this gene in *p. pedunculata*; ^i^ that does not have this gene in *P. dulcis*; ^j^ that does not have this gene in *P. davidiana*; ^k^ that does not have this gene in *P. mongolica*; ^l^ that does not have this gene in *P. triloba*; ^*^ Gene contains one intron; ^**^ gene contains two introns; ^(× 2)^ indicates that the number of the repeat unit is 2

### IR boundary changes and gene rearrangement

**Fig. 2:**
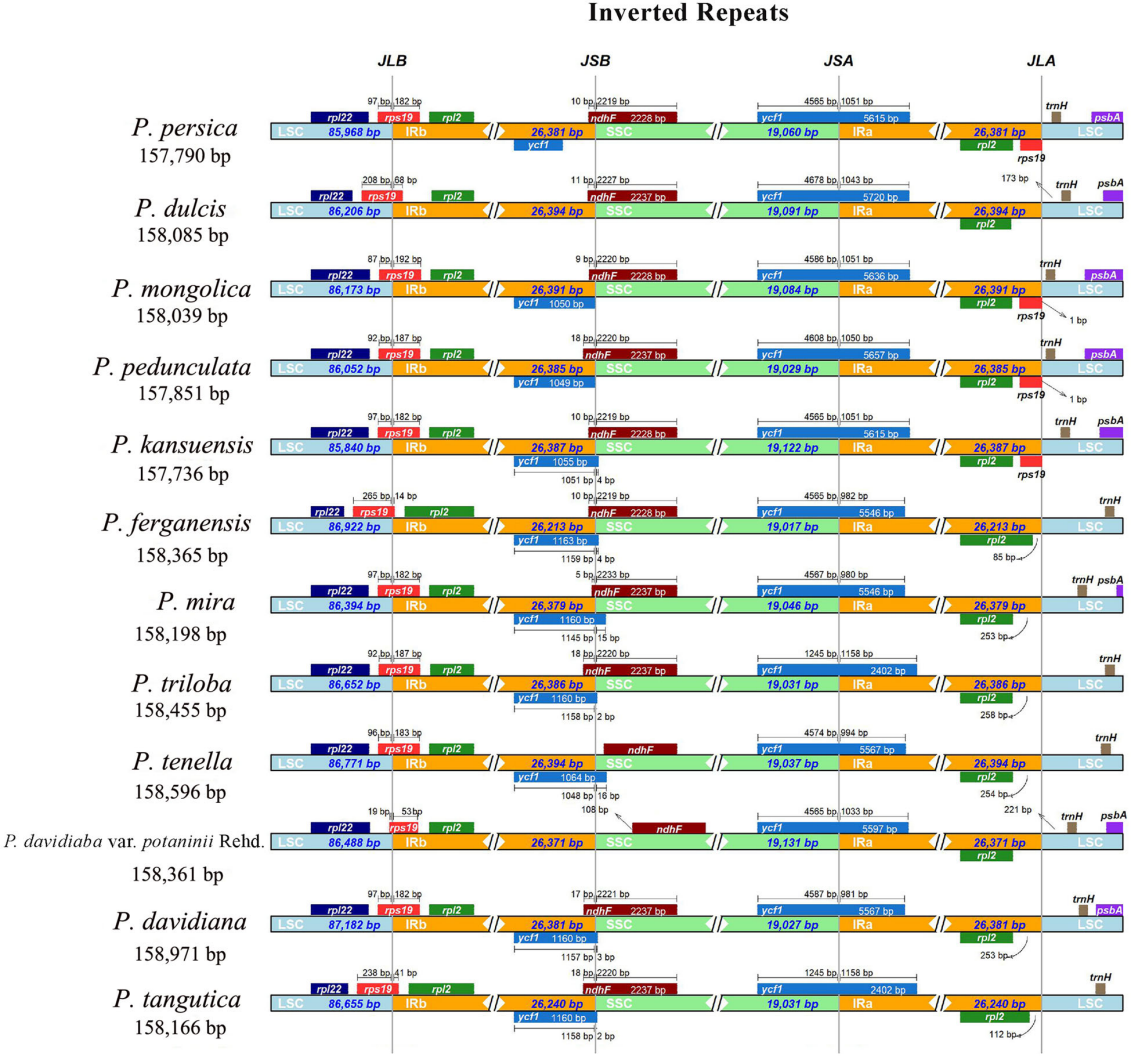
Comparison of the borders for LSC, SSC, and IR regions of complete chloroplast genomes of 12 subg. *Amygdalus* species

The complete cp genome structure of 12 subg. *Amygdalus* species differed. However, all species had eight genes located at the border of the IR region, i.e., *rpl22*, *rps19*, and *rpl2* at LSC/IRb; *ycf1* and *ndhF* at IRb/SSC; *ycf1* at SSC/IRa; and *rpl2* and *trnH* at IRa/LSC. The border between IRb and SSC extended into the *rps19* genes, and the 12 subg. *Amygdalus* species have similar *rps19* pseudogenes. The *ycf1* gene of *P. mongolica* was completely located in the IRb region and was 58 bp away from the IRb/SSC border. The IRb/SSC border extended into the *ycf1* genes in the other genomes with a short *ycf1* pseudogene of 1–16 bp. The IRa region expanded into *trnH* (Fig. 2).

**Fig. 3:**
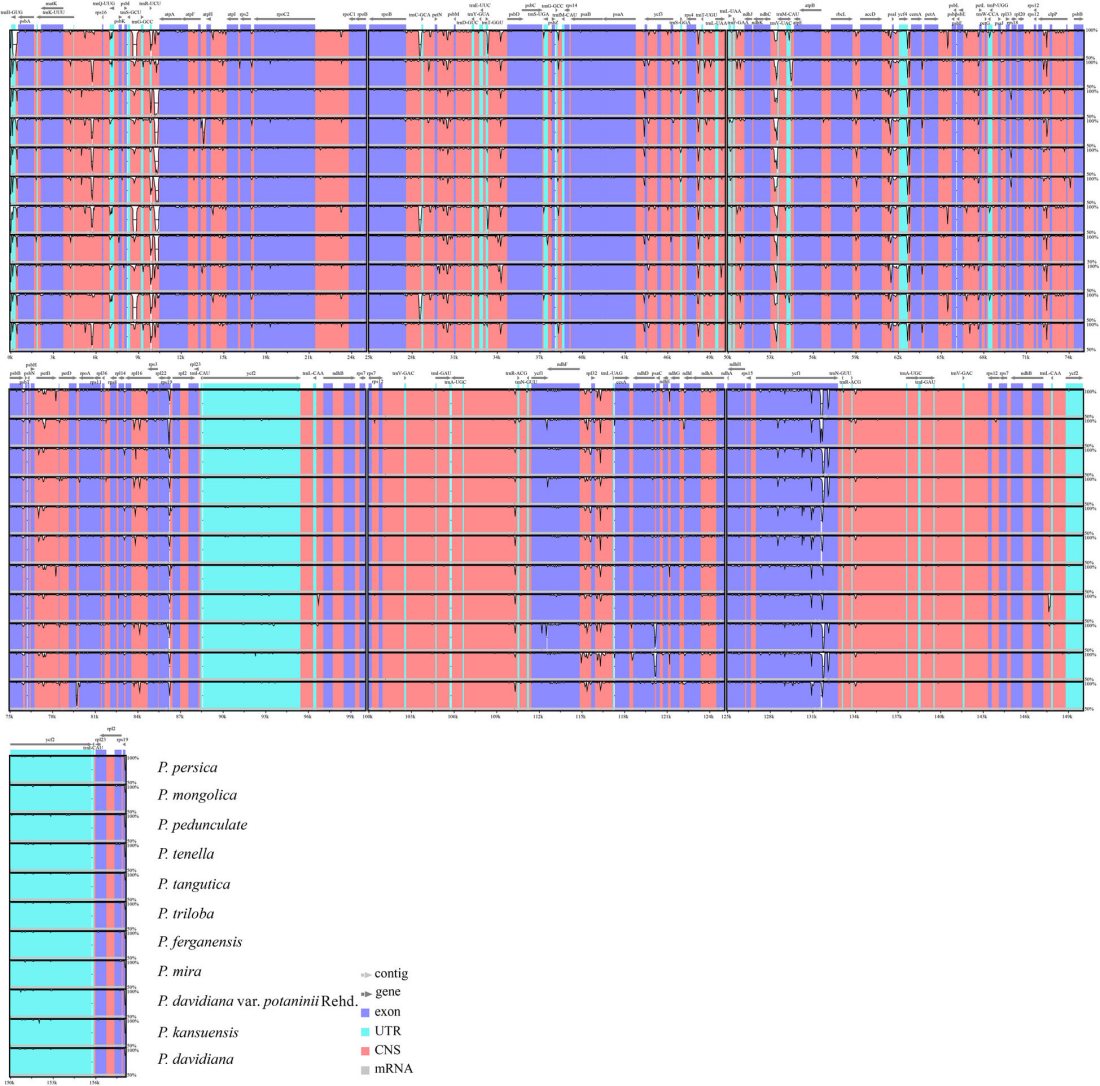
Sequence alignment of complete chloroplast genomes of 12 subg. *Amygdalus* specie

The complete cp genome structure and order of the 12 subg. *Amygdalus* species were relatively conservative; the genomes had high sequence similarity, but there were also some highly variable regions (Fig. 3). These variations mainly existed in non-coding regions. There were substantial differences in intergenic regions in the LSC and SSC regions, including *trnH–psbA, trnK–rps16, petN–psbM, rps4–trnT, ndhC–trnV, ycf4–cemA, rps19–rpl2, trnS–trnG*, and *rpoB–trnC*. In addition, there were differences in the coding region of the *ycf1* gene and the intron region of the *clpP* gene.

**Fig. 4:**
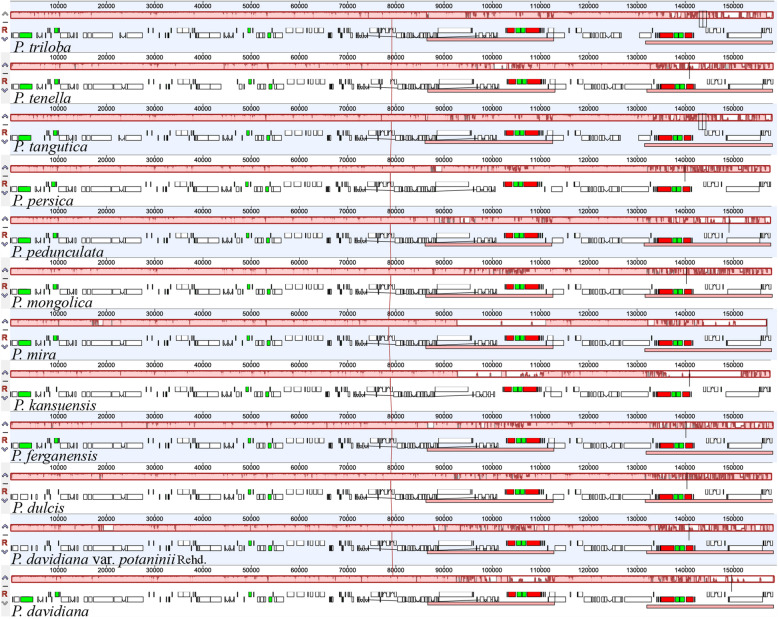
MAUVE genome alignments of complete chloroplast genome of 12 subg. *Amygdalus* species. *P. dulcis* was reference genome

The complete cp genome of *P. dulcis* was considered a reference sequence to compare the remaining subg. *Amygdalus* complete cp genomes. There was no rearrangement in the complete cp genomes of the 12 subg. *Amygdalus* species (Fig. 4).

### Repeats and simple sequence repeats analysis

**Fig. 5:**
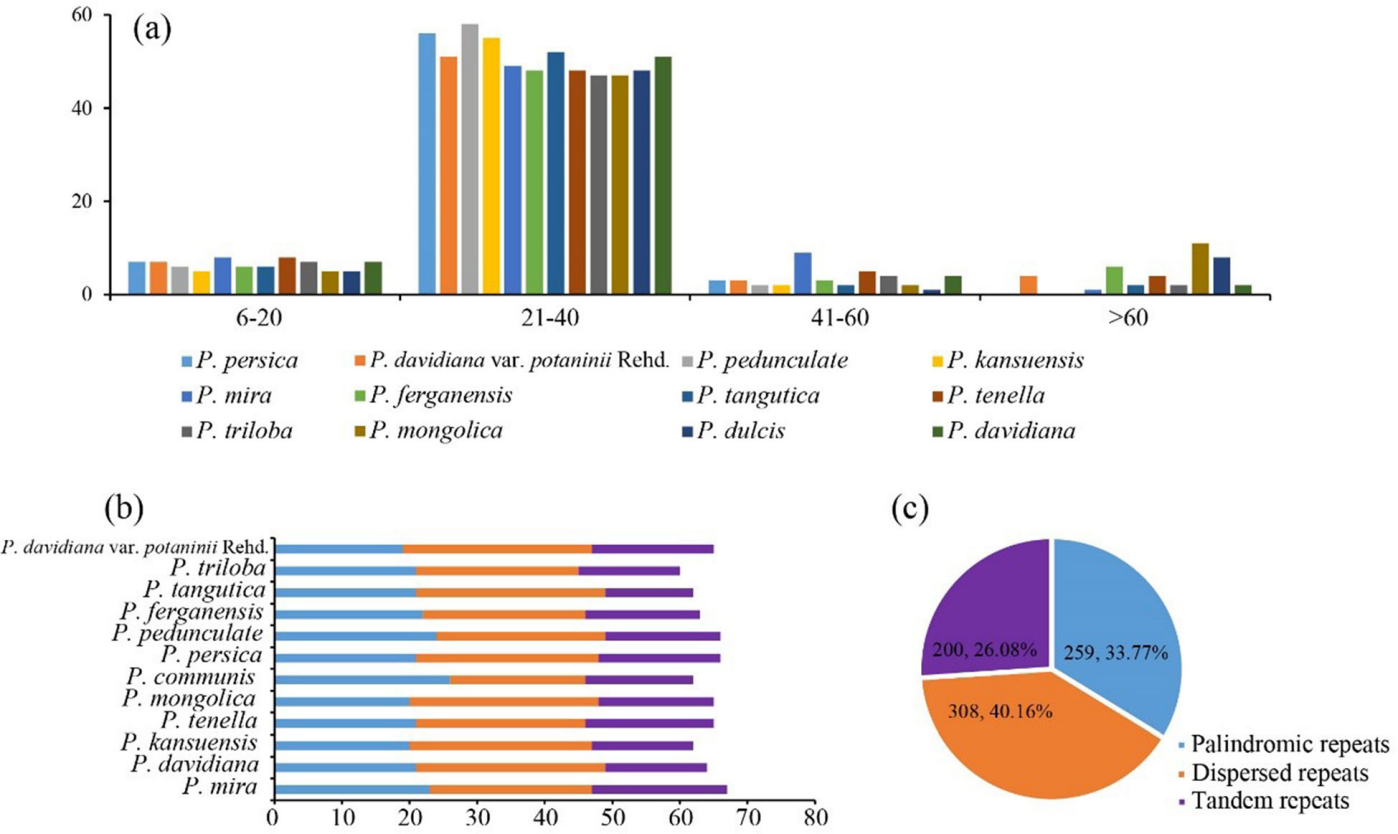
Analysis of repeated sequences of complete chloroplast genomes of 12 subg. *Amygdalus* species. **a** Summary of repeat sequences by length. **b** Numbers of three repeat types. **c** Distribution of repeat sequences

Palindromic repeats, dispersed repeats, and tandem repeats were identified in the complete cp genome sequences of 12 subg. *Amygdalus* species (Fig. 5, Table S1, & Table S2). The numbers and distributions of these three repeats of subg. *Amygdalus* were similar and conservative. There were 308 dispersed repeats, 259 palindrome repeats, and 199 tandem repeats, which accounted for 40.21, 33.81, and 25.98% of the total repeats, respectively. *Prunus mira* had the most repeats, including 24 dispersed repeats, 23 palindromic repeats, and 20 tandem repeats. *P. triloba* had the fewest repeats, including 24 dispersed repeats, 21 palindromic repeats, and 15 tandem repeats. The repeats were concentrated in the region of 24–127 bp; most were distributed in spacers or introns, although a few were distributed in gene regions.

**Fig. 6:**
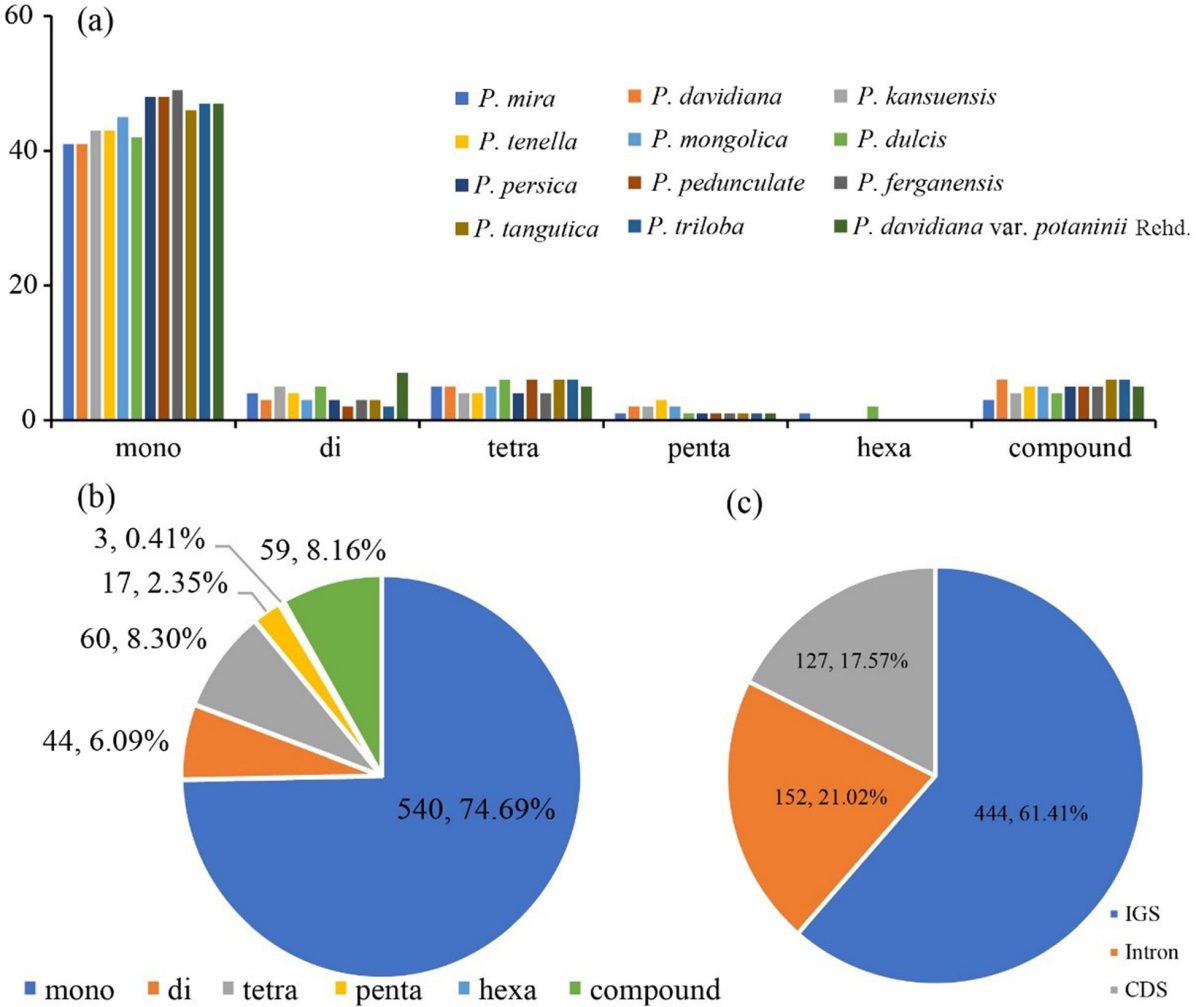
Analysis of SSRs of complete chloroplast genomes of 12 subg. *Amygdalus* species. **a** Number and types of SSRs locations. **b** Summary of SSRs locations. **c** Distribution of SSRs locations

There were 55–65 SSRs in the complete cp genome sequences of the 12 subg. *Amygdalus* species (Fig. 6, Table S3). An average of 82.40% of SSRs were located in the non-coding region of LSC/SSC, and 17.60% of SSRs were located in the PCGs (*matK, rpoC2, rpoB, atpB, rps18, rpl16, ycf1, ycf3, atpF, ndhE, ndhI, psbE*, and *psbZ*). *Prunus mira* and *P. dulcis* have 1 and 2 hexanucleotide repeats, respectively, and mononucleotide, dinucleotide, tetranucleotide, pentanucleotide, and compound nucleotide repeats accounted for averages of 74.69, 6.09, 8.30, 2.35, and 8.16% of all SSRs, respectively. Approximately 90.00% of mononucleotide repeats were A/T repeats. Moreover, there were 4–5 C/G mononucleotide repeats in each genome, and AT/TA was in dinucleotides repeats.

### Chloroplast phylogenetic and divergence time analysis

**Fig. 7:**
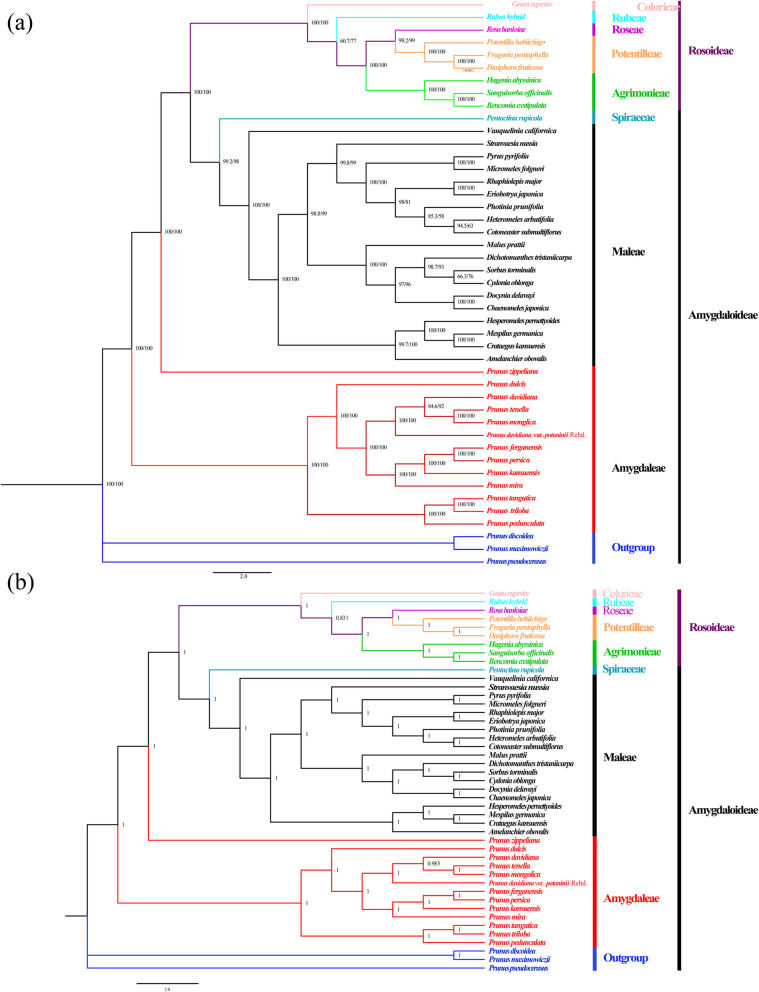
The (**a**) maximum likelihood (ML) and (**b**) bayesian inference (BI) phylogenetic tree of 12 subg. *Amygdalus* species and other 33 species (the NCBI accession numbers see Table S4). **a** The numbers at nodes correspond to ML bootstrap percentages, (**b**) the number of the branches represent Bayesian posterior probability (PP)

Phylogenetic trees were constructed based on the complete cp genome sequences of 45 species. The topologies of the maximum likelihood (ML) and Bayesian inference (BI) trees were nearly identical (Fig. 7 & Table S4). All 12 subg. *Amygdalus* species formed a monophyletic clade that was sister to Maleae and Spiraeae. Colurieae, Rubeae, Roseae, Potentilleae, and Agrimonieae clustered into one clade.

**Fig. 8:**
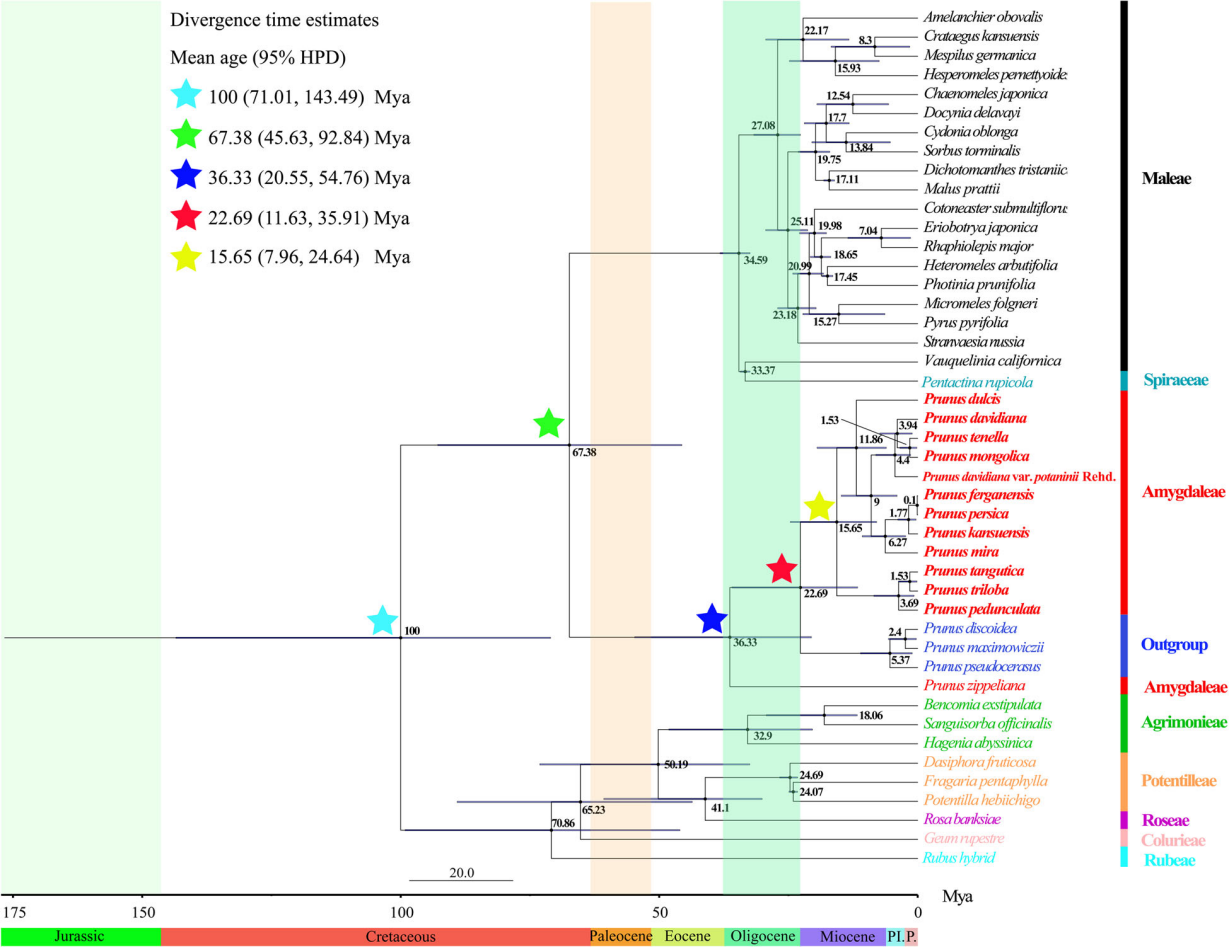
Divergence times of 12 subg. *Amygdalus* species obtained from BEAST analysis based on the complete chloroplast genome sequences. Mean divergence time of the nodes were shown next to the nodes while the blue bars correspond to the 95% highest posterior density (HPD)

Divergence time estimates suggested that the 12 subg. *Amygdalus* species shared a common ancestor around 22.69 Mya (95%HPD: 11.63–35.91 Mya); they diverged into two clades approximately 15.65 Mya (95%HPD: 7.96–24.64 Mya) (*P. dulcis*, *P. davidiana*, *P. tenella*, *P. mongolica*, *P. davidiana* var. *potaninii* Rehd., *P. ferganensis*, *P. kansuensis*, and *P. mira* belonged to one clade; *P. tangutica*, *P. triloba*, and *P. pedunculata* belonged to the other clade). *P. dulcis* was the oldest species of subg. *Amygdalus*, and started to independently evolve around 11.86 Mya (95%HPD: 6.07–19.46 Mya). Diversification within subg. *Amygdalus* occurred over a short period of approximately 0.1 Mya (Fig. 8).

## Discussion

The complete cp genomes of subg. *Amygdalus* species are typical quadripartite structures with LSC, SSC, and two IR regions. These genomes were similar to the previously reported complete cp genomes of *P. mume*, *P. armeniaca*, and *P. salicina* [29]. The cp genomes of most plants are 120–160 kb in size [30], although those of a few plants are 46–190 kb in size; for example, the cp genomes of Orobanchaceae are 46–190 kb in size [31, 32]. In our study, the cp genomes were conserved and similarly sized, with genomes sizes ranging from 157,736 bp in *P. kansuensis* to 158,971 bp in *P. davidiana*. The 12 subg. *Amygdalus* species encoded 130/133 genes. There were relatively longer LSC and SSC regions than IR regions; the pair of inverted IRa/IRb regions was 26,137–26,467 bp, the LSC region was 85,757–86,608 bp, and the SSC region was 19,020–19,133 bp. There were no differences in the GC contents and composition of subg. *Amygdalus* species; this indicates that the complete cp genome structure is relatively stable, and that the overall evolution rate is low [33].

In this study, the complete cp genomes of the 12 subg. *Amygdalus* species ranged from 157,736 bp in *P. tenella* to 158,971 bp in *P. kansuensis*. There were differences of only 1235 bp, which indicated that subg. *Amygdalus* species are highly conservative. Furthermore, there were a total of 130/133 genes present in the complete cp genome of subg. *Amygdalus*, which included 85/86 PCGs, 37 tRNAs, and 8 rRNAs. The GC contents were similar to those of other plants (such as angiosperms), which showed that they are highly conserved [27]. The average GC content of the complete cp genomes in the 12 species was 36.80%, which is similar to the findings of a previously study that studied *Gynostemma* species (GC content, 36.9–37.0%) [27]. This high GC content could be caused by the high GC content of the rRNA gene sequences located in IR regions. The function, order, and GC content of these genes are all highly conserved, which was also noted for other angiosperms [27]. The complete cp genomes of the 12 subg. *Amygdalus* species had an average GC content of 36.80%, which is consistent with the results of *Gynostemma* species genomes [27], and may be caused by the a high GC content in IR regions [27].

Size increases of plastomes are usually caused by expansion of the IR regions, which are the most conserved regions in the complete cp genome [34]. Our results showed that the cpDNA structure of the 12 subg. *Amygdalus* species slightly different from each other, although they all have eight genes located at the border of the IR region, i.e., *rpl22*, *rps19*, and *rpl2* at LSC/IRb; *ycf1* and *ndhF* at IRb/SSC; *ycf1* at SSC/IRa; and *rpl2* and *trnH* at IRa/LSC. The border between the IRb and SSC extended into the *rps19* genes, and there were *rps19* pseudogenes in the 12 subg. *Amygdalus* species. The expansion of the IR region into the *rps19* and *ycf1* genes is also present in *Cardiocrinum* and *Amana* [35, 36]. These significant differences may contribute to the development of molecular markers and genetic barcodes for subg. *Amygdalus* species.

SSRs are widely distributed throughout the genome and play important roles in genome recombination and rearrangement; in particular, polymorphic SSRs can be used to study genetic diversity, population structure, and biogeography within and between groups [37]. We identified 55–65 SSRs in the complete cp genomes of the 12 subg. *Amygdalus* species; on average, 82.40% of SSRs were located in the non-coding LSC or SSC regions, and 17.60% of SSRs were located in the protein-coding region. Furthermore, SSRs are dominated by single nucleotide repeats, and approximately 90% of single nucleotide repeats were A/T repeats in this study. A previous study revealed that the repeated sequences may play a very important role in sequence rearrangement of complete cp genomes [38]. The results of palindromic repeats, dispersed palindromic repeats, and tandem repeats showed that the number and distribution of these repeats in the 12 species of subg. *Amygdalus* species were similar and conservative. *Prunus mira* had a maximum of 24 scattered repeats, 23 palindromes, and 20 tandem repeats. *P. triloba* had the fewest repeats.

Phylogenetic relationships in Rosaceae have long been problematic because of frequent hybridization, apomixis, presumed rapid radiation, and historical diversification [29]. Development of the cp phylogeny and time estimation provides new evidence for future comparative evolutionary studies [29], and there have been an increasing number of studies using complete cp genome sequences to assess phylogenetic relationships among angiosperms [27, 39]. Our phylogenetic tree was based on complete cp genome data. The ML and BI methods were used to conduct phylogenetic analysis on the 12 subg. *Amygdalus* species. The phylogenetic trees indicated that the 12 subg. *Amygdalus* species were clearly closely related with high bootstrap support and posterior probabilities (Fig. 7). The results showed that the 12 subg. *Amygdalus* species were divided into one subclade, which consistent with results of Yazbek et al. [5]. In addition, the results are consistent with the traditional classification system; this indicated that the current classification of the 12 subg. *Amygdalus* species is reasonable, such as the classification used in the Flora of China (www.iplant.cn/frps). In morphological cladistic analysis, the appearance, shape, and other characteristics of the 12 subg. *Amygdalus* species were similar. However, Vafadar et al. [40] suggested that *P. mira* Koehne, *P. davidiana* (Carriere) Franch., *P. triloba* Ltdl., and *P. tenella* L. should be excluded from *Amygdalus*, and this may require further study. There are differences in the morphological tree structure and molecular phylogeny of the Rosaceae family and relationships among various genera. Therefore, these cp genome sequences will provide genetic information that may help elucidate the evolution of these species.

The divergence time of the 12 subg. *Amygdalus* was estimated. The results showed that the 12 subg. *Amygdalus* species shared a common ancestor around 22.69 Mya, and the two clades diverged approximately 15.65 Mya (95%HPD: 7.96–24.64 Mya). According to fossil evidence from southwest China, peach (*P. persica*) was present in the late Pliocene (ca. 2.6 Mya) [41]; however, we found that *P. persica* diverged from *P. ferganensis* approximately 0.1 Mya. In addition, Liu et al. [42] showed that *P. persica* diverged approximately 10.0 Mya based on plastid *ndhF*, *rps16*, and *rpl16* sequence data, which may be based on different data. Moreover, our results showed that Amygdaleae diverged approximately 36.33 Mya. The Rosaceae patterns indicate that hybridization and polyploidy may have played a pivotal role in the early evolution of the family in the Eocene [43], which indicated that the Rosaceae was different about Eocene (53–36.5 Mya), they are similar to the results of our study.

## Conclusion

This study reported the complete cp genome sequences of subg. *Amygdalus* species, which further enriches the availability of valuable complete cp genomes of subg. *Amygdalus* species. The results showed that subg. *Amygdalus* species exhibited a tetrad structure, with sizes ranging from 157,736 bp in *P. kansuensis* to 158,971 bp in *P. davidiana*; the pair of inverted IRa/IRb regions ranged from 26,137–26,467 bp, the LSC region ranged from 85,757–86,608 bp, and the SSC region ranged from 19,020–19,133 bp. The average GC content of the complete cp genome in the 12 species was 36.80%. In addition, it was found that the structure of the subg. *Amygdalus* complete cp genome was highly conserved, and all 12 subg. *Amygdalus* species had an *rps*19 pseudogene. There was no rearrangement in the complete cp of the 12 subg. *Amygdalus* species. All 12 subg. *Amygdalus* species clustered into one clade based on both the BI and ML methods. The divergence time analyses based on the complete cp genome sequences showed that subg. *Amygdalus* species events occurred approximately 15.65 Mya. Our study provides data on the phylogenetic structure of subg. *Amygdalus* and its phylogeny position with *Prunus*, and offers a reference for the divergence time of subg. *Amygdalus*.

## Materials and methods

### Plant material sampling and chloroplast genomic DNA extraction

Fresh and healthy leaves were collected from adult *P. pedunculate*, *P. mira*, *P. ferganensis*, *P. tangutica*, *P. tenella*, *P. triloba*, *P. mongolica*, *P. dulcis*, and *P. davidiana* var. *potaninii* Rehd. plants in the field in northwest China (Table 3). The voucher specimens were placed in the herbarium of the School of Life Science, Yulin University, and the spare materials were placed in an ultra-low temperature refrigerator at − 80 °C (for accession numbers, see Table 3; all nine subg. *Amygdalus* specimens were identified and sorted by Yizhong Duan). *Prunus persica*, *P. davidiana*, and *P. kansuensis* sequences were collected from the GenBank database (Table 1). The modified hexadecyl trimethyl ammonium bromide method [44, 45] was used to extract the total genomic DNA of the 9 species. The extracted DNA was subjected to 0.5% agarose gel electrophoresis and ultraviolet spectrophotometer to check the quality. After passing the total genomic DNA test, fragment it with ultrasound; then, fragment purification and end repair were performed, A was added at the 3′ end, and the sequencing adapter was connected. Subsequently, we used agarose gel electrophoresis to select the size of the fragment and performed polymerase chain reaction for sequencing library preparation. The built library was first subjected to library quality inspection. The library that passed quality inspection was sequenced by Beijing Biomax Biotechnology Co., Ltd. (http://www.biomarker.com.cn/) using the Illumina HiSeq Xten-PE150 platform.

**Table 3:** The collection location of plants material

No.	Species name	Longitude (E)	Latitude (N)	Above sea level (m)	Accession number in herbarium
1	*P. dulcis*	78°16′14.4″	37°27′51.1″	1809	20180901YI05
2	*P. mira*	113°42′25.3″	34°42′43.6″	107	20190702YI01
3	*P. ferganensis*	113°42′25.3″	34°42′43.6″	107	20180910YI03
4	*P. davidiana* var. *potaninii* Rehd.	113°42′25.3″	34°42′43.6″	107	20190702YI02
5	*P. tangutica*	113°42′25.3″	34°42′43.6″	107	20190702YI03
6	*P. triloba*	109°42′59.4″	38°17′37.2″	1081	20180802YI02
7	*P. pedunculate*	103°50′49.0″	38°35′15.8″	1354	20180903Yl01
8	*P. tenella*	103°50′49.0″	38°35′15.8″	1354	20180903Yl02
9	*P. mongolica*	105°48′4.0″	38°39′35.0″	1979	20180502YI01

### Chloroplast genome assembly and annotation

Raw sequencing read (raw read) data were stored in FASTQ format. The FASTQ data were filtered to obtain clean reads. For data filtering, reads were removed if: (1) they had an adapter; (2) the N content exceeded 10%; (3) they had a base value of less than 10 with a quality value exceeding 50%. The filtered reads were assembled using SOAPdenovo software [46] (http://soap.genomics.org.cn/soap denovo.html), and the assembly was then optimized according to the paired-end and overlap of reads result. For some gaps in the sequence, the assembly result was filled and corrected by SOAPdenovo software to obtain a complete cp genome. The OrganellarGenomeDRAW (OGDraw) online annotation software [47] (http://phylocluster.biosci. Ttexas.edu/dogma/) was used to annotate the complete cp genomes of the nine collected subg. *Amygdalus* species, and the remaining three species of subg. *Amygdalus* plants genomes were downloaded from the National Center for Biotechnology Information (NCBI) database (Table 1). The complete cp genome of *P. pseudocerasus* Lindl. (NC030599) [48] was used as the reference and was manually revised and annotated with Geneious R8 software [49]. Finally, OGDraw visualization software [50] (https://chlorobox.mpimp-golm.mpg.de/OGDraw.html) was used to draw a physical map of complete cp genomes of subg. *Amygdalus* species.

### Chloroplast genome sequence comparative analysis

The complete cp genome sequences of the 12 subg. *Amygdalus* species were compared. The IR, LSC, and SSC areas, and their boundary information were compared; the online IRscope tool (https://irscope.shinyapps.io/irapp/) [51] was used to map the IR boundary contrast figure. The differences of complete cp genome sequences of the 12 subg. *Amygdalus* species were studied, and the *P. dulcis* genome was used as the reference sequence. Each complete cp genome sequence annotation file format of “bed” was converted, which were uploaded to the online analysis program mVISTA (http://genome.lbl.gov/vista/mvista/submit.shtml) [52], and the Shuffle-Lagan mode was selected for genome-wide comparison. The complete cp genome sequence was imported into Geneious R8 software, and the mauve plug-in was used to alignment of global [53]. The gene rearrangement was detected by collinearity analysis with *P. dulcis* (MT019559) as a reference sequence.

### Chloroplast genome repeat sequence identification and SSR analysis

Three types of repeated sequences (palindromic repeats, dispersed repeats, and tandem repeats) of all 12 subg. *Amygdalus* species were searched and identified by the online REPuter software (https://bibiserv.cebitec.uni-bielefeld.de/reputer/manual.html) [54]. In this study, the parameters had a minimal repeat size of 20 bp and the Hamming distance was 3. Tandem repeat sequences were identified by the online software Tandem Repeats Finder (http://tandem.bu.edu/trf/trf.html) [55]. The alignment parameters for match, mismatch, and indels were 2, 7, and 7, respectively. The minimum alignment scores of reported repeats, maximum period size, and maximum TR array size were 80 bp, 500 bp, and 2 bp, respectively. SSR locations were identified by the online MISA software (https://webblast.ipk-gatersleben.de/misa/) [56]. Moreover, the minimum numbers of repeats of mononucleotides, dinucleotides, trinucleotides, tetranucleotides, pentanucleotides, and hexanucleotides were 10, 6, 5, 3, 3, and 3, respectively.

### Chloroplast phylogenetic relationships and divergence time estimate

### Chloroplast phylogenetic relationships

The complete cp genomes of 33 species from the NCBI database (https://www.ncbi.nlm.nih.gov/) and the 12 subg. *Amygdalus* species were selected to construct a phylogenetic tree; the complete cp genomes were obtained for nine species of Rosoideae, 19 species of Maleae, 1 species of Sporaeeae, 13 species of Amygdaleae, *P. discoidea*, *P. maximowiczii*, and *P. pseudocerasus* (for the NCBI accession numbers, see Table S4). First, the complete cp genomes of the 45 species were examined by multiple sequence alignment in MAFFT using the PhyloSuite software [57, 58]. Subsequently, the comparison results were imported into the ModelFinder program [59], and Akaike information criterion was selected for nucleotide substitution model testing. Then, the “.nex” files in the result were imported into the MrBayes program [60] to build a phylogenetic tree based on the BI method. *Prunus discoidea*, *P. maximowiczii*, and *P. pseudocerasus* were outgroups in reconstruction of the BI phylogenetic tree. We chose the GTR model and GAMMA distribution, and a Markov chain Monte Carlo with one cold and three heated chains [61, 62]. Analyses were run for 2,000,000 generations total, sampled once every 1000 generations, running end when the value of the average standard deviation of split frequencies was less than 0.01. We discarded the less than 25% of aging samples and constructed a consistent tree according to the remaining samples.

Then, based on the Akaike information criterion, the ModelFinder program was run again to select the optimal nucleotide substitution model from the nucleotide substitution models suitable for BEAST 2 analysis. The results were imported into the IQ-TREE program [63]; the GTR + I + G + F4 model and an ultrafast bootstrap approximation algorithm were selected [64]. The number of re-sampling was 10,000, and the SH-aLRT test was enabled to re-sample 1000 times [65]. The consistent tree file “.contree” was imported into Figtree v1.4.4 software to view and edit the tree, and the output tree diagram was saved to the file [66].

### Divergence time estimate

The BEAST v2.6.0 program [62] was used to estimate the divergence time of subg. *Amygdalus*. First, the MAFFT comparison results were imported to BEAUTi v2, and GTR + Gamma was selected as the nucleotide substitution model (shape = 0.241) [61]. “Empirical” was set of the basic frequency, and the strict molecular clock model was selected. Second, the “Yule Model” was used as the system tree model, and we set the number of iterations and sampling in the Markov chain Monte Carlo algorithm to 3,000,000 and 1000, respectively. Finally, the “.xml” file was obtained from BEAST v2.6.0 [62]. The result was imported into Tracer v1.7.1 software (http://tree.bio.ed.ac.uk /software/tracer/) to check the effective sample size; the effective sample size was greater than 200, which means the results were robust. We used the TreeAnnotator program (https://beast.community/index.html) [67] to perform optimal tree merging. Burn-in was set to 10%, the optimal tree was saved as the final result, and Figtree v1.4.4 software [66] was used to view phylogenetic trees with different time estimates.

We estimated the divergence time of the 12 subg. *Amygdalus* species with the TimeTree tool (http://www.timetree.org) [68] using the BI tree. We selected four nodes to determine the divergence time: (1) *Pentactina rupicola* and *Vauquelinia californica* diverged 62 Mya (range, 30–70 Mya) [69–71]; (2) *Duchesnea chrysantha* and *Fragaria pentaphylla* diverged 45 Mya (range, 22–52 Mya) [69, 70, 72–76]; (3) *M. prattii* and *Dichotomanthes tristaniicarpa* diverged 32.0 Mya (range, 19.4–46.4 Mya) [70, 77]; (5) *Photinia prunifolia* and *H. arbutifolia* diverged 32.9 Mya (range, 21.0–46.4 Mya) [70, 77]. The standard deviation of four nodes was 1.0 Mya. Finally, we analyzed the dated phylogeny in Figtree, and the value of node representing the tMRCA and 95%HPD were displayed in Figtree to make the results more intuitive.

## Supplementary Information

**Additional file 1: Table S1.** The repeats distribution in the chloroplast genomes of 12 subg. *Amygdalus* species. **Table S2.** The tandem repeats distribution in the chloroplast genomes of 12 subg. *Amygdalus* species. **Table S3.** Simple sequence repeats in the chloroplast genomes of 12 subg. *Amygdalus* species. **Table S4.** List of species accessions numbers were used in phylogenetic analysis.

## Data Availability

The data-sets used and/or analysed during the current study available from the corresponding author on reasonable request.
