# Supplementary material for: The chloroplast genome of Amygdalus L. (Rosaceae) reveals the phylogenetic relationship and divergence time
Source: BMC Genomics. 2021 Sep 7;22:645. doi: 10.1186/s12864-021-07968-6 (PMC8425060; doi:10.1186/s12864-021-07968-6)
Supplement: Supplementary file 1 — Additional file 1: Table S1. The repeats distribution in the chloroplast genomes of 12 subg. Amygdalus species. Table S2. The tandem repeats distribution in the chloroplast genomes of 12 subg. Amygdalus species. Table S3. Simple sequence repeats in the chloroplast genomes of 12 subg. Amygdalus species. Table S4. List of species accessions numbers were used in phylogenetic analysis. [file 12864_2021_7968_MOESM1_ESM.docx]

**The chloroplast genome of *Amygdalus* L. (Rosaceae)** **reveals the phylogenetic relationship and divergence time**

**Zhongyu Du^1,2,#^, Ke Lu^1,#^, Kai Zhang^1^, Yiming He^1^, Haitao Wang^1^, Guaiqiang Chai^1^, Jianguo Shi^1^, Yizhong Duan^1*^**

^1^ College of life science / Shaanxi Key Laboratory of Ecological Restoration in Northern Shaanxi Mining Area/, Yulin University, Yulin, China,

^2^ School of ecology and environment / Breeding Base for State Key Laboratory of Land Degradation and Ecological Restoration in Northwest China / Ministry of Education Key Laboratory for Restoration and Reconstruction of Degraded Ecosystems in Northwest China, Ningxia University, Yinchuan, China

**# these authors contributed equally to this study.**

* **Correspondence:** Yizhong Duan

**E-mail:** duanyizhong2006@163.com

**Supplementary Materials**

**Table S1** The repeats distribution in the chloroplast genomes of 12 subg. *Amygdalus* species.

**Table S2** The tandem repeats distribution in the chloroplast genomes of 12 subg. *Amygdalus* species.

**Table S3** Simple sequence repeats in the chloroplast genomes of 12 subg. *Amygdalus* species.

**Table S4** List of species accessions numbers were used in phylogenetic analysis.

**Table S1 The repeats distribution in the chloroplast genomes of 12 subg. *Amygdalus* species.**

| **Species name** | [**Repetitive**](javascript:;) [**mode**](javascript:;) |  | **Size** | **Position1** | **Type** | **Position2** | **Location1** | **Location2** | **Region1** | **Region2** |
| --- | --- | --- | --- | --- | --- | --- | --- | --- | --- | --- |
| *P. dulcis* | Dispersed repeats |  | 40 | 40559 | F | 42783 | *psaB* | *psaA* | LSC | LSC |
|  |  |  | 38 | 45565 | F | 123390 | *ycf3** | *ndhA** | LSC | SSC |
|  |  |  | 39 | 45565 | F | 100975 | *ycf3** | *rps7-trnV-GAC* | LSC | IRA |
|  |  |  | 24 | 48134 | F | 48158 | *rps4-trnT-UGU* | *rps4-trnT-UGU* | LSC | LSC |
|  |  |  | 32 | 62588 | F | 62620 | *ycf4* | *ycf4-cemA* | LSC | LSC |
|  |  |  | 40 | 86080 | F | 86206 | *rps19* | *rps19* | LSC | IRA |
|  |  |  | 87 | 86119 | F | 86246 | *rps19* | *rps19-rpl2* | LSC | IRA |
|  |  |  | 29 | 91200 | F | 91221 | *ycf2* | *ycf2* | IRA | IRA |
|  |  |  | 34 | 93619 | F | 93637 | *ycf2* | *ycf2* | IRA | IRA |
|  |  |  | 40 | 100973 | F | 123388 | *rps7-trnV-GAC* | *ndhA** | IRA | SSC |
|  |  |  | 33 | 109910 | F | 109941 | *rrn4.5-rrn5* | *rrn4.5-rrn5* | IRA | IRA |
|  |  |  | 24 | 112169 | F | 132098 | *ycf1* | *ycf1* | IRA | IRB |
|  |  |  | 84 | 131568 | F | 131691 | *ycf1* | *ycf1* | SSC | SSC |
|  |  |  | 92 | 131560 | F | 131683 | *ycf1* | *ycf1* | SSC | SSC |
|  |  |  | 32 | 131653 | F | 131779 | *ycf1* | *ycf1* | SSC | IRB |
|  |  |  | 33 | 134317 | F | 134348 | *rrn5 -rrn4.5* | *rrn5 -rrn4.5* | IRB | IRB |
|  |  |  | 28 | 150626 | F | 150644 | *ycf2* | *ycf2* | IRB | IRB |
|  |  |  | 34 | 150620 | F | 150638 | *ycf2* | *ycf2* | IRB | IRB |
|  |  |  | 24 | 52481 | R | 52481 | *ndhK-ndhC* | *ndhK-ndhC* | LSC | LSC |
|  |  |  | 26 | 61101 | R | 61101 | *accD-psaI* | *accD-psaI* | LSC | LSC |
|  | Palindromic repeats |  | 40 | 87 | P | 86080 | *trnH-GUG* | *rps19* | LSC | LSC |
|  |  |  | 32 | 133 | P | 53262 | *trnH-GUG* | *ndhC -trnV-UAC* | LSC | LSC |
|  |  |  | 34 | 5718 | P | 5718 | *rps16** | *rps16** | LSC | LSC |
|  |  |  | 30 | 8282 | P | 47048 | *trnS-GCU* | *trnS-GGA-rps4* | LSC | LSC |
|  |  |  | 53 | 9876 | P | 9876 | *trnR-UCU-atpA* | *trnR-UCU-atpA* | LSC | LSC |
|  |  |  | 33 | 13492 | P | 13492 | *atpF-atpH* | *atpF-atpH* | LSC | LSC |
|  |  |  | 38 | 29840 | P | 29840 | *petN -psbM* | *petN -psbM* | LSC | LSC |
|  |  |  | 34 | 30451 | P | 30491 | *petN -psbM* | *petN -psbM* | LSC | LSC |
|  |  |  | 35 | 45566 | P | 77523 | *ycf3** | *petB** | LSC | LSC |
|  |  |  | 39 | 45565 | P | 143277 | *ycf3** | *trnV-GAC-rps7* | LSC | IRB |
|  |  |  | 31 | 48738 | P | 48738 | *trnT-UGU-trnL-UAA* | *trnT-UGU-trnL-UAA* | LSC | LSC |
|  |  |  | 35 | 69224 | P | 69224 | *psaJ-rpl33* | *psaJ-rpl33* | LSC | LSC |
|  |  |  | 38 | 76194 | P | 76194 | *psbT-psbN* | *psbT-psbN* | LSC | LSC |
|  |  |  | 40 | 86080 | P | 158045 | *rps19* | *rps19* | LSC | IRB |
|  |  |  | 87 | 86119 | P | 157958 | *rps19* | *rps19* | LSC | IRB |
|  |  |  | 29 | 91200 | P | 153041 | *ycf2* | *ycf2* | IRA | IRB |
|  |  |  | 29 | 91221 | P | 153062 | *ycf2* | *ycf2* | IRA | IRB |
|  |  |  | 34 | 93619 | P | 150620 | *ycf2* | *ycf2* | IRA | IRB |
|  |  |  | 34 | 93637 | P | 150638 | *ycf2* | *ycf2* | IRA | IRB |
|  |  |  | 33 | 109910 | P | 134317 | *rrn4.5-rrn5* | *rrn5 -rrn4.5* | IRA | IRB |
|  |  |  | 33 | 109941 | P | 134348 | *rrn4.5-rrn5* | *rrn5 -rrn4.5* | IRA | IRB |
|  |  |  | 24 | 112169 | P | 112169 | *ycf1* | *ycf1* | IRA | IRA |
|  |  |  | 32 | 112480 | P | 131653 | *ycf1* | *ycf1* | IRA | SSC |
|  |  |  | 91 | 112516 | P | 131561 | *ycf1* | *ycf1* | SSC | SSC |
|  |  |  | 40 | 123388 | P | 143278 | *ndhA** | *trnV-GAC-rps7* | SSC | IRB |
|  |  |  | 24 | 132098 | P | 132098 | *ycf1* | *ycf1* | IRB | IRB |
| *P. davidiana var. potaninii* Rehd. | Dispersed repeats |  | 23 | 151 | F | 191 | *rps19 -trnH-GUG* | *rps19 -trnH-GUG* | LSC | LSC |
|  |  |  | 22 | 34175 | F | 34194 | *trnT-GGU-psbD* | *trnT-GGU-psbD* | LSC | LSC |
|  |  |  | 23 | 34370 | F | 34392 | *trnT-GGU-psbD* | *trnT-GGU-psbD* | LSC | LSC |
|  |  |  | 40 | 40671 | F | 42895 | *psaB* | *psaA* | LSC | LSC |
|  |  |  | 25 | 49843 | F | 49866 | *trnL-UAA** | *trnL-UAA** | LSC | LSC |
|  |  |  | 22 | 53514 | F | 53533 | *ndhC -trnV-UAC* | *ndhC -trnV-UAC* | LSC | LSC |
|  |  |  | 23 | 61609 | F | 61630 | *accD-psaI* | *accD-psaI* | LSC | LSC |
|  |  |  | 29 | 91467 | F | 91488 | *ycf2* | *ycf2* | IRA | IRA |
|  |  |  | 34 | 93889 | F | 93907 | *ycf2* | *ycf2* | IRA | IRA |
|  |  |  | 39 | 45685 | F | 101250 | *ycf3 ** | *rps7-trnV-GAC* | LSC | IRA |
|  |  |  | 33 | 110185 | F | 110216 | *rrn4.5 -rrn5* | *rrn4.5 -rrn5* | IRA | IRA |
|  |  |  | 127 | 112731 | F | 112858 | *ycf1-ndhF* | *ycf1-ndhF* | IRA | SSC |
|  |  |  | 24 | 115728 | F | 115757 | *ndhF-rpl32* | *ndhF-rpl32* | SSC | SSC |
|  |  |  | 38 | 45685 | F | 123805 | *ycf3 ** | *ndhA** | LSC | SSC |
|  |  |  | 40 | 101248 | F | 123803 | *rps7-trnV-GAC* | *ndhA** | IRA | SSC |
|  |  |  | 33 | 134600 | F | 134631 | *rrn5-rrn4.5* | *rrn5-rrn4.5* | IRB | IRB |
|  |  |  | 25 | 43905 | F | 148743 | *psaA-ycf3* | *trnL-CAA-ycf2* | LSC | IRB |
|  |  |  | 28 | 150914 | F | 150932 | *ycf2* | *ycf2* | IRB | IRB |
|  |  |  | 34 | 150908 | F | 150926 | *ycf2* | *ycf2* | IRB | IRB |
|  |  |  | 29 | 153332 | F | 153353 | *ycf2* | *ycf2* | IRB | IRB |
|  |  |  | 44 | 83 | F | 158317 | *rps19 -trnH-GUG* | *rps19 -trnH-GUG* | LSC | IRB |
|  |  |  | 32 | 10153 | R | 10153 | *trnR-UCU- atpA* | *trnR-UCU- atpA* | LSC | LSC |
|  |  |  | 23 | 50978 | R | 50978 | *trnF-GAA-ndhJ* | *trnF-GAA-ndhJ* | LSC | LSC |
|  |  |  | 23 | 52611 | R | 52611 | *ndhK-ndhC* | *ndhK-ndhC* | LSC | LSC |
|  |  |  | 29 | 61227 | R | 61227 | *accD-psaI* | *accD-psaI* | LSC | LSC |
|  |  |  | 28 | 65798 | R | 65798 | *petA-psbJ* | *petA-psbJ* | LSC | LSC |
|  |  |  | 25 | 116684 | R | 116684 | *rpl32 -trnL-UAG* | *rpl32 -trnL-UAG* | SSC | SSC |
|  |  |  | 32 | 116690 | R | 116690 | *rpl32 -trnL-UAG* | *rpl32 -trnL-UAG* | SSC | SSC |
|  | Palindromic repeats |  | 53 | 9898 | P | 9898 | *trnG-GCC-trnR-UCU* | *trnG-GCC-trnR-UCU* | LSC | LSC |
|  |  |  | 33 | 13547 | P | 13547 | *atpF -atpH* | *atpF -atpH* | LSC | LSC |
|  |  |  | 61 | 16970 | P | 16970 | *rps2 -rpoC2* | *rps2 -rpoC2* | LSC | LSC |
|  |  |  | 30 | 8299 | P | 47168 | *psbI-trnS-GCU* | *trnS-GGA* | LSC | LSC |
|  |  |  | 31 | 48834 | P | 48834 | *trnT-UGU-trnL-UAA* | *trnT-UGU-trnL-UAA* | LSC | LSC |
|  |  |  | 37 | 69669 | P | 69669 | *psaJ-rpl33* | *psaJ-rpl33* | LSC | LSC |
|  |  |  | 38 | 76618 | P | 76618 | *psbT-psbN* | *psbT-psbN* | LSC | LSC |
|  |  |  | 35 | 45686 | P | 77942 | *ycf3 ** | *petB** | LSC | LSC |
|  |  |  | 44 | 83 | P | 86488 | *rps19 -trnH-GUG* | *rps19* | LSC | IRA |
|  |  |  | 25 | 43905 | P | 96081 | *psaA-ycf3* | *ycf2-trnL-CAA* | LSC | IRA |
|  |  |  | 127 | 112858 | P | 131991 | *ycf1-ndhF* | *ycf1* | SSC | IRB |
|  |  |  | 33 | 110185 | P | 134600 | *rrn4.5 -rrn5* | *rrn5-rrn4.5* | IRA | IRB |
|  |  |  | 33 | 110216 | P | 134631 | *rrn4.5 -rrn5* | *rrn5-rrn4.5* | IRA | IRB |
|  |  |  | 39 | 45685 | P | 143560 | *ycf3 ** | *trnV-GAC-rps7* | LSC | IRB |
|  |  |  | 40 | 123803 | P | 143561 | *ndhA** | *trnV-GAC-rps7* | SSC | IRB |
|  |  |  | 34 | 93889 | P | 150908 | *ycf2* | *ycf2* | IRA | IRB |
|  |  |  | 34 | 93907 | P | 150926 | *ycf2* | *ycf2* | IRA | IRB |
|  |  |  | 29 | 91467 | P | 153332 | *ycf2* | *ycf2* | IRA | IRB |
|  |  |  | 29 | 91488 | P | 153353 | *ycf2* | *ycf2* | IRA | IRB |
| *P. davidiana* | Dispersed repeats |  | 31 | 5050 | F | 5074 | *trnK-UUU-rps16* | *trnK-UUU-rps16* | LSC | LSC |
|  |  |  | 26 | 38141 | F | 38167 | *psbZ-trnG-GCC* | *psbZ-trnG-GCC* | LSC | LSC |
|  |  |  | 40 | 40465 | F | 42689 | *psaB* | *psaA* | LSC | LSC |
|  |  |  | 22 | 67202 | F | 67221 | *psbE-petL* | *psbE-petL* | LSC | LSC |
|  |  |  | 29 | 91227 | F | 91248 | *ycf2* | *ycf2* | IRA | IRA |
|  |  |  | 28 | 93646 | F | 93664 | *ycf2* | *ycf2* | IRA | IRA |
|  |  |  | 39 | 45494 | F | 101002 | *ycf3** | *rps7-trnV-GAC* | LSC | IRA |
|  |  |  | 33 | 109937 | F | 109968 | *rrn4.5-rrn5* | *rrn4.5-rrn5* | IRA | IRA |
|  |  |  | 24 | 115371 | F | 115394 | *ndhF -rpl32* | *ndhF -rpl32* | SSC | SSC |
|  |  |  | 38 | 116334 | F | 116372 | *rpl33-trnL-UAG* | *rpl32-trnL-UAG* | SSC | SSC |
|  |  |  | 57 | 116334 | F | 116353 | *rpl32-trnL-UAG* | *rpl32-trnL-UAG* | SSC | SSC |
|  |  |  | 38 | 45494 | F | 123489 | *ycf3** | *ndhA ** | LSC | SSC |
|  |  |  | 40 | 101000 | F | 123487 | *rps7-trnV-GAC* | *ndhA ** | IRA | SSC |
|  |  |  | 24 | 112190 | F | 132089 | *ycf1* | *ycf1* | IRA | IRB |
|  |  |  | 33 | 134302 | F | 134333 | *rrn5-rrn4.5* | *rrn5-rrn4.5* | IRB | IRB |
|  |  |  | 25 | 98361 | F | 145917 | *ndhB** | *ndhB ** | IRA | IRB |
|  |  |  | 25 | 43699 | F | 148440 | *psaA-ycf3* | *trnL-CAA-ycf2* | LSC | IRB |
|  |  |  | 28 | 150611 | F | 150629 | *ycf2* | *ycf2* | IRB | IRB |
|  |  |  | 29 | 153026 | F | 153047 | *ycf2* | *ycf2* | IRB | IRB |
|  |  |  | 23 | 52400 | R | 52400 | *ndhK-ndhC* | *ndhK-ndhC* | LSC | LSC |
|  |  |  | 27 | 60992 | R | 60992 | *accD-psaI* | *accD-psaI* | LSC | LSC |
|  |  |  | 29 | 10042 | R | 115385 | *trnR-UCU-atpA* | *ndhF-rpl32* | LSC | SSC |
|  |  |  | 25 | 116334 | R | 116334 | *rpl34-trnL-UAG* | *rpl32-trnL-UAG* | SSC | SSC |
|  |  |  | 44 | 116334 | R | 116334 | *rpl32-trnL-UAG* | *rpl32-trnL-UAG* | SSC | SSC |
|  |  |  | 63 | 116334 | R | 116334 | *rpl32-trnL-UAG* | *rpl32-trnL-UAG* | SSC | SSC |
|  |  |  | 32 | 116378 | R | 116378 | *rpl37-trnL-UAG* | *rpl32-trnL-UAG* | SSC | SSC |
|  |  |  | 51 | 116359 | R | 116359 | *rpl36-trnL-UAG* | *rpl32-trnL-UAG* | SSC | SSC |
|  |  |  | 70 | 116340 | R | 116340 | *rpl35-trnL-UAG* | *rpl32-trnL-UAG* | SSC | SSC |
|  | Palindromic repeats |  | 53 | 9737 | P | 9737 | *trnG-GCC-trnR-UCU* | *trnG-GCC-trnR-UCU* | LSC | LSC |
|  |  |  | 33 | 13376 | P | 13376 | *atpF-atpH* | *atpF-atpH* | LSC | LSC |
|  |  |  | 27 | 16803 | P | 16836 | *rps2-rpoC2* | *rps2-rpoC2* | LSC | LSC |
|  |  |  | 30 | 8141 | P | 46977 | *trnS-GCU* | *trnS-GGA* | LSC | LSC |
|  |  |  | 31 | 48644 | P | 48644 | *trnT-UGU-trnL-UAA* | *trnT-UGU-trnL-UAA* | LSC | LSC |
|  |  |  | 35 | 69418 | P | 69418 | *psaJ-rpl33* | *psaJ-rpl33* | LSC | LSC |
|  |  |  | 38 | 76360 | P | 76360 | *psbT-psbN* | *psbT-psbN* | LSC | LSC |
|  |  |  | 35 | 45495 | P | 77683 | *ycf3** | *petB** | LSC | LSC |
|  |  |  | 25 | 43699 | P | 95838 | *psaA-ycf3* | *ycf2 -trnL-CAA* | LSC | IRA |
|  |  |  | 25 | 98361 | P | 98361 | *ndhB** | *ndhB** | IRA | IRA |
|  |  |  | 24 | 112190 | P | 112190 | *ycf1* | *ycf1* | IRA | IRA |
|  |  |  | 24 | 132089 | P | 132089 | *ycf1* | *ycf1* | IRB | IRB |
|  |  |  | 33 | 109937 | P | 134302 | *rrn4.5-rrn5* | *rrn5-rrn4.5* | IRA | IRB |
|  |  |  | 33 | 109968 | P | 134333 | *rrn4.5-rrn5* | *rrn5-rrn4.5* | IRA | IRB |
|  |  |  | 39 | 45494 | P | 143262 | *ycf3** | *trnV-GAC-rps7* | LSC | IRB |
|  |  |  | 40 | 123487 | P | 143263 | *ndhA ** | *trnV-GAC-rps7* | SSC | IRB |
|  |  |  | 25 | 145917 | P | 145917 | *ndhB ** | *ndhB ** | IRB | IRB |
|  |  |  | 28 | 93646 | P | 150611 | *ycf2* | *ycf2* | IRA | IRB |
|  |  |  | 28 | 93664 | P | 150629 | *ycf2* | *ycf2* | IRA | IRB |
|  |  |  | 29 | 91227 | P | 153026 | *ycf2* | *ycf2* | IRA | IRB |
|  |  |  | 29 | 91248 | P | 153047 | *ycf2* | *ycf2* | IRA | IRB |
| *P. ferganensis* | Dispersed repeats |  | 24 | 5262 | F | 5285 | *trnK-UUU-rps16* | *trnK-UUU-rps16* | LSC | LSC |
|  |  |  | 40 | 40544 | F | 42768 | *psaB* | *psaA* | LSC | LSC |
|  |  |  | 37 | 53347 | F | 53370 | *ndhC -trnV-UAC* | *ndhC -trnV-UAC* | LSC | LSC |
|  |  |  | 26 | 72755 | F | 72794 | *clpP** | *clpP** | LSC | LSC |
|  |  |  | 29 | 91451 | F | 91472 | *ycf2* | *ycf2* | IRA | IRA |
|  |  |  | 34 | 93870 | F | 93888 | *ycf2* | *ycf2* | IRA | IRA |
|  |  |  | 39 | 45552 | F | 101226 | *ycf3 ** | *rps7-trnV-GAC* | LSC | IRA |
|  |  |  | 33 | 110161 | F | 110192 | *rrn4.5-rrn5* | *rrn4.5-rrn5* | IRA | IRA |
|  |  |  | 40 | 116529 | F | 116548 | *rpl32-trnL-UAG* | *rpl32-trnL-UAG* | SSC | SSC |
|  |  |  | 38 | 45552 | F | 123680 | *ycf3 ** | *ndhA** | LSC | SSC |
|  |  |  | 40 | 101224 | F | 123678 | *rps7-trnV-GAC* | *ndhA** | IRA | SSC |
|  |  |  | 24 | 112414 | F | 132285 | *ycf1* | *ycf1* | IRA | IRB |
|  |  |  | 33 | 134498 | F | 134529 | *rrn5 -rrn4.5* | *rrn5 -rrn4.5* | IRB | IRB |
|  |  |  | 34 | 150801 | F | 150819 | *ycf2* | *ycf2* | IRB | IRB |
|  |  |  | 28 | 150807 | F | 150825 | *ycf2* | *ycf2* | IRB | IRB |
|  |  |  | 31 | 14122 | R | 14122 | *atpH -atpI* | *atpH -atpI* | LSC | LSC |
|  |  |  | 24 | 52444 | R | 52444 | *ndhK-ndhC* | *ndhK-ndhC* | LSC | LSC |
|  |  |  | 25 | 53382 | R | 53382 | *ndhC -trnV-UAC* | *ndhC -trnV-UAC* | LSC | LSC |
|  |  |  | 30 | 65652 | R | 65652 | *petA-psbJ* | *petA-psbJ* | LSC | LSC |
|  |  |  | 32 | 116535 | R | 116554 | *rpl32-trnL-UAG* | *rpl32-trnL-UAG* | SSC | SSC |
|  |  |  | 32 | 116554 | R | 116554 | *rpl32-trnL-UAG* | *rpl32-trnL-UAG* | SSC | SSC |
|  | Palindromic repeats |  | 33 | 13417 | P | 13417 | *atpF-atpH* | *atpF-atpH* | LSC | LSC |
|  |  |  | 32 | 14118 | P | 14118 | *atpH -atpI* | *atpH -atpI* | LSC | LSC |
|  |  |  | 30 | 14126 | P | 14126 | *atpH -atpI* | *atpH -atpI* | LSC | LSC |
|  |  |  | 36 | 38209 | P | 38209 | *psbZ-trnG-UCC* | *psbZ-trnG-UCC* | LSC | LSC |
|  |  |  | 30 | 8399 | P | 47044 | *psbI-trnS-GCU* | *trnS-GGA* | LSC | LSC |
|  |  |  | 31 | 48707 | P | 48707 | *trnT-UGU -trnL-UAA* | *trnT-UGU -trnL-UAA* | LSC | LSC |
|  |  |  | 34 | 53394 | P | 53400 | *ndhC -trnV-UAC* | *ndhC -trnV-UAC* | LSC | LSC |
|  |  |  | 37 | 69553 | P | 69553 | *psaJ-rpl33* | *psaJ-rpl33* | LSC | LSC |
|  |  |  | 38 | 76553 | P | 76553 | *psbT-psbN* | *psbT-psbN* | LSC | LSC |
|  |  |  | 35 | 45553 | P | 77882 | *ycf3 ** | *petB** | LSC | LSC |
|  |  |  | 24 | 112414 | P | 112414 | *ycf1* | *ycf1* | IRA | IRA |
|  |  |  | 24 | 132285 | P | 132285 | *ycf1* | *ycf1* | IRB | IRB |
|  |  |  | 33 | 110161 | P | 134498 | *rrn4.5-rrn5* | *rrn4.5-rrn5* | IRA | IRB |
|  |  |  | 33 | 110192 | P | 134529 | *rrn4.5-rrn5* | *rrn4.5-rrn5* | IRA | IRB |
|  |  |  | 39 | 45552 | P | 143458 | *ycf3 ** | *rrn4.5-rrn5* | LSC | IRB |
|  |  |  | 40 | 123678 | P | 143459 | *ndhA** | *rrn4.5-rrn5* | SSC | IRB |
|  |  |  | 34 | 93870 | P | 150801 | *ycf2* | *ycf2* | IRA | IRB |
|  |  |  | 34 | 93888 | P | 150819 | *ycf2* | *ycf2* | IRA | IRB |
| *P. kansuensis* | Dispersed repeats |  | 40 | 40061 | F | 42285 | *psaB* | *psaA* | LSC | LSC |
|  |  |  | 29 | 90733 | F | 90754 | *ycf2* | *ycf2* | IRA | IRA |
|  |  |  | 34 | 93158 | F | 93176 | *ycf2* | *ycf2* | IRA | IRA |
|  |  |  | 39 | 45083 | F | 100514 | *ycf3* | *rps7-trnV-GAC* | LSC | IRA |
|  |  |  | 33 | 109449 | F | 109480 | *rrn4.5 -rrn5* | *rrn4.5 -rrn5* | IRA | IRA |
|  |  |  | 26 | 115651 | F | 115676 | *rpl32-trnL-UAG* | *rpl32-trnL-UAG* | SSC | SSC |
|  |  |  | 31 | 115876 | F | 115895 | *rpl32-trnL-UAG* | *rpl32-trnL-UAG* | SSC | SSC |
|  |  |  | 37 | 115870 | F | 115889 | *rpl32-trnL-UAG* | *rpl32-trnL-UAG* | SSC | SSC |
|  |  |  | 37 | 115870 | F | 115908 | *rpl32-trnL-UAG* | *rpl32-trnL-UAG* | SSC | SSC |
|  |  |  | 34 | 115895 | F | 115914 | *rpl32-trnL-UAG* | *rpl32-trnL-UAG* | SSC | SSC |
|  |  |  | 38 | 45083 | F | 123073 | *ycf3* | *ndhA ** | LSC | SSC |
|  |  |  | 40 | 100512 | F | 123071 | *rps7 -trnV-GAC* | *ndhA ** | IRA | SSC |
|  |  |  | 24 | 111702 | F | 131678 | *ycf1* | *ycf1* | IRA | IRB |
|  |  |  | 33 | 133891 | F | 133922 | *rrn5-rrn4.5* | *rrn5-rrn4.5* | IRB | IRB |
|  |  |  | 28 | 150200 | F | 150218 | *ycf2* | *ycf2* | IRB | IRB |
|  |  |  | 34 | 150194 | F | 150212 | *ycf2* | *ycf2* | IRB | IRB |
|  |  |  | 29 | 152621 | F | 152642 | *ycf2* | *ycf2* | IRB | IRB |
|  |  |  | 31 | 13837 | R | 13837 | *atpH-atpI* | *atpH-atpI* | LSC | LSC |
|  |  |  | 29 | 45727 | R | 45727 | *ycf3* | *ycf3* | LSC | LSC |
|  |  |  | 24 | 51990 | R | 51990 | *ndhK -ndhC* | *ndhK -ndhC* | LSC | LSC |
|  |  |  | 24 | 60645 | R | 60645 | *accD-psaI* | *accD-psaI* | LSC | LSC |
|  |  |  | 30 | 65045 | R | 65045 | *petA-psbJ* | *petA-psbJ* | LSC | LSC |
|  |  |  | 37 | 115870 | R | 115877 | *rpl32-trnL-UAG* | *rpl32-trnL-UAG* | SSC | SSC |
|  |  |  | 36 | 115871 | R | 115896 | *rpl32-trnL-UAG* | *rpl32-trnL-UAG* | SSC | SSC |
|  |  |  | 38 | 115876 | R | 115908 | *rpl32-trnL-UAG* | *rpl32-trnL-UAG* | SSC | SSC |
|  |  |  | 32 | 115895 | R | 115914 | *rpl32-trnL-UAG* | *rpl32-trnL-UAG* | SSC | SSC |
|  |  |  | 32 | 115914 | R | 115914 | *rpl32-trnL-UAG* | *rpl32-trnL-UAG* | SSC | SSC |
|  | Palindromic repeats |  | 53 | 9492 | P | 9492 | *trnG-GCC-trnR-UCU* | *trnG-GCC-trnR-UCU* | LSC | LSC |
|  |  |  | 33 | 13132 | P | 13132 | *atpF -atpH* | *atpF -atpH* | LSC | LSC |
|  |  |  | 32 | 13833 | P | 13833 | *atpH-atpI* | *atpH-atpI* | LSC | LSC |
|  |  |  | 30 | 13841 | P | 13841 | *atpH-atpI* | *atpH-atpI* | LSC | LSC |
|  |  |  | 30 | 8180 | P | 46566 | *trnS-GCU* | *trnS-GGA* | LSC | LSC |
|  |  |  | 31 | 48237 | P | 48237 | *trnT-UGU-trnL-UAA* | *trnT-UGU-trnL-UAA* | LSC | LSC |
|  |  |  | 43 | 52774 | P | 52774 | *ndhC -trnV-UAC* | *ndhC -trnV-UAC* | LSC | LSC |
|  |  |  | 37 | 68892 | P | 68892 | *psaJ -rpl33* | *psaJ -rpl33* | LSC | LSC |
|  |  |  | 38 | 75851 | P | 75851 | *psbT-psbN* | *psbT-psbN* | LSC | LSC |
|  |  |  | 35 | 45084 | P | 77182 | *ycf3* | *petB** | LSC | LSC |
|  |  |  | 24 | 111702 | P | 111702 | *ycf1* | *ycf1* | IRA | IRA |
|  |  |  | 24 | 131678 | P | 131678 | *ycf1* | *ycf1* | IRB | IRB |
|  |  |  | 33 | 109449 | P | 133891 | *rrn4.5 -rrn5* | *rrn5-rrn4.5* | IRA | IRB |
|  |  |  | 33 | 109480 | P | 133922 | *rrn4.5 -rrn5* | *rrn5-rrn4.5* | IRA | IRB |
|  |  |  | 39 | 45083 | P | 142851 | *ycf3* | *trnV-GAC-rps7* | LSC | IRB |
|  |  |  | 40 | 123071 | P | 142852 | *ndhA** | *trnV-GAC-rps7* | SSC | IRB |
|  |  |  | 34 | 93158 | P | 150194 | *ycf2* | *ycf2* | IRA | IRB |
|  |  |  | 34 | 93176 | P | 150212 | *ycf2* | *ycf2* | IRA | IRB |
|  |  |  | 29 | 90733 | P | 152621 | *ycf2* | *ycf2* | IRA | IRB |
|  |  |  | 29 | 90754 | P | 152642 | *ycf2* | *ycf2* | IRA | IRB |
| *P.* *mongolica* | Dispersed repeats |  | 25 | 10085 | F | 10108 | *trnR-UCU-atpA* | *trnR-UCU-atpA* | LSC | LSC |
|  |  |  | 40 | 40450 | F | 42674 | *psaB* | *psaA* | LSC | LSC |
|  |  |  | 25 | 48917 | F | 48920 | *trnT-UGU-trnL-UAA* | *trnT-UGU-trnL-UAA* | LSC | LSC |
|  |  |  | 26 | 48917 | F | 48919 | *trnT-UGU-trnL-UAA* | *trnT-UGU-trnL-UAA* | LSC | LSC |
|  |  |  | 27 | 48917 | F | 48918 | *trnT-UGU-trnL-UAA* | *trnT-UGU-trnL-UAA* | LSC | LSC |
|  |  |  | 81 | 86310 | F | 86391 | *rps19* | *rps19* | IRA | IRA |
|  |  |  | 34 | 93804 | F | 93822 | *ycf2* | *ycf2* | IRA | IRA |
|  |  |  | 39 | 45453 | F | 101165 | *ycf3** | *rps7-trnV-GAC* | LSC | IRA |
|  |  |  | 33 | 110100 | F | 110131 | *rrn4.5 -rrn5* | *rrn4.5 -rrn5* | IRA | IRA |
|  |  |  | 83 | 112713 | F | 112796 | *ycf1-ndhF* | *ycf1-ndhF* | IRA | IRA |
|  |  |  | 31 | 116570 | F | 116585 | *rpl32-trnL-UAG* | *rpl32-trnL-UAG* | SSC | IRA |
|  |  |  | 38 | 45453 | F | 123733 | *ycf3** | *ndhA** | LSC | SSC |
|  |  |  | 40 | 101163 | F | 123731 | *rps7-trnV-GAC* | *ndhA** | IRA | SSC |
|  |  |  | 33 | 134574 | F | 134605 | *rrn5-rrn4.5* | *rrn5-rrn4.5* | IRB | IRB |
|  |  |  | 28 | 150888 | F | 150906 | *ycf2* | *ycf2* | IRB | IRB |
|  |  |  | 34 | 150882 | F | 150900 | *ycf2* | *ycf2* | IRB | IRB |
|  |  |  | 83 | 157959 | F | 158023 | *rps19-trnH-GUG* | *rps19-trnH-GUG* | IRB | IRB |
|  |  |  | 33 | 48445 | R | 48445 | *trnT-UGU-trnL-UAA* | *trnT-UGU-trnL-UAA* | LSC | LSC |
|  |  |  | 26 | 48917 | R | 48917 | *trnT-UGU-trnL-UAA* | *trnT-UGU-trnL-UAA* | LSC | LSC |
|  |  |  | 27 | 48917 | R | 48917 | *trnT-UGU-trnL-UAA* | *trnT-UGU-trnL-UAA* | LSC | LSC |
|  |  |  | 25 | 48920 | R | 48920 | *trnT-UGU-trnL-UAA* | *trnT-UGU-trnL-UAA* | LSC | LSC |
|  |  |  | 26 | 48919 | R | 48919 | *trnT-UGU-trnL-UAA* | *trnT-UGU-trnL-UAA* | LSC | LSC |
|  |  |  | 27 | 48918 | R | 48918 | *trnT-UGU-trnL-UAA* | *trnT-UGU-trnL-UAA* | LSC | LSC |
|  |  |  | 30 | 48916 | R | 48916 | *trnT-UGU-trnL-UAA* | *trnT-UGU-trnL-UAA* | LSC | LSC |
|  |  |  | 35 | 48907 | R | 48917 | *trnT-UGU-trnL-UAA* | *trnT-UGU-trnL-UAA* | LSC | LSC |
|  |  |  | 27 | 50779 | R | 50779 | *trnF-GAA-ndhJ* | *trnF-GAA-ndhJ* | LSC | LSC |
|  |  |  | 26 | 61020 | R | 61020 | *accD-psaI* | *accD-psaI* | LSC | LSC |
|  |  |  | 33 | 116602 | R | 116602 | *rpl32-trnL-UAG* | *rpl32-trnL-UAG* | SSC | IRA |
|  | Palindromic repeats |  | 53 | 9725 | P | 9725 | *trnG-GCC -trnR-UCU* | *trnG-GCC -trnR-UCU* | LSC | LSC |
|  |  |  | 33 | 13358 | P | 13358 | *atpF-atpH* | *atpF-atpH* | LSC | LSC |
|  |  |  | 59 | 16818 | P | 16818 | *rps2-rpoC2* | *rps2-rpoC2* | LSC | LSC |
|  |  |  | 30 | 8126 | P | 46949 | *trnS-GCU* | *trnS-GGA* | LSC | LSC |
|  |  |  | 31 | 48630 | P | 48630 | *trnT-UGU-trnL-UAA* | *trnT-UGU-trnL-UAA* | LSC | LSC |
|  |  |  | 35 | 69442 | P | 69442 | *psaJ-rpl33* | *psaJ-rpl33* | LSC | LSC |
|  |  |  | 38 | 76404 | P | 76404 | *psbT-psbN* | *psbT-psbN* | LSC | LSC |
|  |  |  | 35 | 45454 | P | 77752 | *ycf3** | *petB** | LSC | LSC |
|  |  |  | 25 | 83977 | P | 116634 | *rpl16** | *rpl32-trnL-UAG* | LSC | IRA |
|  |  |  | 83 | 112796 | P | 131942 | *ycf1-ndhF* | *ycf1* | SSC | IRB |
|  |  |  | 33 | 110100 | P | 134574 | *rrn4.5 -rrn5* | *rrn5-rrn4.5* | IRA | IRB |
|  |  |  | 33 | 110131 | P | 134605 | *rrn4.5 -rrn5* | *rrn5-rrn4.5* | IRA | IRB |
|  |  |  | 39 | 45453 | P | 143534 | *ycf3** | *trnV-GAC-rps7* | LSC | IRB |
|  |  |  | 40 | 123731 | P | 143535 | *ndhA** | *trnV-GAC-rps7* | SSC | IRB |
|  |  |  | 34 | 93804 | P | 150882 | *ycf2* | *ycf2* | IRA | IRB |
|  |  |  | 34 | 93822 | P | 150900 | *ycf2* | *ycf2* | IRA | IRB |
|  |  |  | 66 | 86325 | P | 157962 | *rps19* | *rps19-trnH-GUG* | IRA | IRB |
|  |  |  | 79 | 86312 | P | 157986 | *rps19* | *rps19-trnH-GUG* | IRA | IRB |
|  |  |  | 76 | 86315 | P | 157993 | *rps19* | *rps19-trnH-GUG* | IRA | IRB |
|  |  |  | 83 | 86396 | P | 157948 | *rps19* | *rps19-trnH-GUG* | IRA | IRB |
| *P.* *pedunculata* | Dispersed repeats |  | 40 | 40195 | F | 42419 | *psaB* | *psaA* | LSC | LSC |
|  |  |  | 32 | 52885 | F | 52907 | *ndhC-trnV-UAC* | *ndhC-trnV-UAC* | LSC | LSC |
|  |  |  | 22 | 53035 | F | 53057 | *ndhC-trnV-UAC* | *ndhC-trnV-UAC* | LSC | LSC |
|  |  |  | 29 | 91058 | F | 91079 | *ycf2* | *ycf2* | IRA | IRA |
|  |  |  | 34 | 93477 | F | 93495 | *ycf2* | *ycf2* | IRA | IRA |
|  |  |  | 39 | 45222 | F | 100833 | *ycf3** | *rps7-trnV-GAC* | LSC | IRA |
|  |  |  | 33 | 109768 | F | 109799 | *rrn4.5 -rrn5* | *rrn4.5 -rrn5* | IRA | IRA |
|  |  |  | 38 | 45222 | F | 123257 | *ycf3** | *ndhA** | LSC | SSC |
|  |  |  | 40 | 100831 | F | 123255 | *rps7-trnV-GAC* | *ndhA** | IRA | SSC |
|  |  |  | 23 | 128403 | F | 128424 | *ycf1* | *ycf1* | SSC | SSC |
|  |  |  | 24 | 112021 | F | 131902 | *ycf1* | *ycf1* | IRA | IRB |
|  |  |  | 33 | 134115 | F | 134146 | *rrn5-rrn4.5* | *rrn5-rrn4.5* | IRB | IRB |
|  |  |  | 25 | 98192 | F | 145730 | *ndhB** | *ndhB ** | IRA | IRB |
|  |  |  | 25 | 43429 | F | 148253 | *psaA-ycf3* | *trnL-CAA-ycf15* | LSC | IRB |
|  |  |  | 28 | 150424 | F | 150442 | *ycf2* | *ycf2* | IRB | IRB |
|  |  |  | 34 | 150418 | F | 150436 | *ycf2* | *ycf2* | IRB | IRB |
|  |  |  | 29 | 152839 | F | 152860 | *ycf2* | *ycf2* | IRB | IRB |
|  |  |  | 28 | 48196 | R | 48196 | *trnT-UGU-trnL-UAA* | *trnT-UGU-trnL-UAA* | LSC | LSC |
|  |  |  | 23 | 52120 | R | 52120 | *ndhK-ndhC* | *ndhK-ndhC* | LSC | LSC |
|  |  |  | 36 | 52896 | R | 52902 | *ndhC-trnV-UAC* | *ndhC-trnV-UAC* | LSC | LSC |
|  |  |  | 29 | 58684 | R | 58684 | *rbcL-accD* | *rbcL-accD* | LSC | LSC |
|  |  |  | 33 | 58692 | R | 58692 | *rbcL-accD* | *rbcL-accD* | LSC | LSC |
|  |  |  | 23 | 60790 | R | 60790 | *accD-psaI* | *accD-psaI* | LSC | LSC |
|  |  |  | 27 | 116141 | R | 116141 | *rpl32-trnL-UAG* | *rpl32-trnL-UAG* | SSC | SSC |
|  |  |  | 32 | 116148 | R | 116148 | *rpl32-trnL-UAG* | *rpl32-trnL-UAG* | SSC | SSC |
|  | Palindromic repeats |  | 22 | 6979 | P | 6979 | *rps16-trnQ-UUG* | *rps16-trnQ-UUG* | LSC | LSC |
|  |  |  | 53 | 9732 | P | 9732 | *trnG-GCC-trnR-UCU* | *trnG-GCC-trnR-UCU* | LSC | LSC |
|  |  |  | 33 | 13130 | P | 13130 | *atpF -atpH* | *atpF -atpH* | LSC | LSC |
|  |  |  | 38 | 29489 | P | 29489 | *petN-psbM* | *petN-psbM* | LSC | LSC |
|  |  |  | 30 | 8140 | P | 46713 | *trnS-GCU* | *trnS-GGA* | LSC | LSC |
|  |  |  | 31 | 48377 | P | 48377 | *trnT-UGU-trnL-UAA* | *trnT-UGU-trnL-UAA* | LSC | LSC |
|  |  |  | 57 | 69166 | P | 69166 | *psaJ -rpl33* | *psaJ -rpl33* | LSC | LSC |
|  |  |  | 31 | 16573 | P | 72371 | *rps2-rpoC2* | *clpP** | LSC | LSC |
|  |  |  | 38 | 76148 | P | 76148 | *psbN* | *psbN* | LSC | LSC |
|  |  |  | 25 | 67287 | P | 76982 | *psbE-petL* | *petB ** | LSC | LSC |
|  |  |  | 35 | 45223 | P | 77479 | *ycf3** | *petB** | LSC | LSC |
|  |  |  | 25 | 43429 | P | 95669 | *psaA-ycf3* | *ycf15-trnL-CAA* | LSC | IRA |
|  |  |  | 25 | 98192 | P | 98192 | *ndhB** | *ndhB** | IRA | IRA |
|  |  |  | 24 | 112021 | P | 112021 | *ycf1* | *ycf1* | IRA | IRA |
|  |  |  | 24 | 131902 | P | 131902 | *ycf1* | *ycf1* | IRB | IRB |
|  |  |  | 33 | 109768 | P | 134115 | *rrn4.5 -rrn5* | *rrn5-rrn4.5* | IRA | IRB |
|  |  |  | 33 | 109799 | P | 134146 | *rrn4.5 -rrn5* | *rrn5-rrn4.5* | IRA | IRB |
|  |  |  | 39 | 45222 | P | 143075 | *ycf3** | *trnV-GAC-rps7* | LSC | IRB |
|  |  |  | 40 | 123255 | P | 143076 | *ndhA** | *trnV-GAC-rps7* | SSC | IRB |
|  |  |  | 25 | 145730 | P | 145730 | *ndhB ** | *ndhB ** | IRB | IRB |
|  |  |  | 34 | 93477 | P | 150418 | *ycf2* | *ycf2* | IRA | IRB |
|  |  |  | 34 | 93495 | P | 150436 | *ycf2* | *ycf2* | IRA | IRB |
|  |  |  | 29 | 91058 | P | 152839 | *ycf2* | *ycf2* | IRA | IRB |
|  |  |  | 29 | 91079 | P | 152860 | *ycf2* | *ycf2* | IRA | IRB |
| *P.* *persica* | Dispersed repeats |  | 24 | 4997 | F | 5020 | *trnK-UUU -rps16* | *trnK-UUU -rps16* | LSC | LSC |
|  |  |  | 40 | 40041 | F | 42265 | *psaB* | *psaA* | LSC | LSC |
|  |  |  | 35 | 53743 | F | 53759 | *trnV-UAC-trnM-CAU* | *trnV-UAC-trnM-CAU* | LSC | LSC |
|  |  |  | 29 | 90861 | F | 90882 | *ycf2* | *ycf2* | IRA | IRA |
|  |  |  | 34 | 93280 | F | 93298 | *ycf2* | *ycf2* | IRA | IRA |
|  |  |  | 39 | 45049 | F | 100636 | *ycf3** | *rps7-trnV-GAC* | LSC | IRA |
|  |  |  | 33 | 109571 | F | 109602 | *rrn4.5-rrn5* | *rrn4.5-rrn5* | IRA | IRA |
|  |  |  | 26 | 115744 | F | 115769 | *rpl32-trnL-UAG* | *rpl32-trnL-UAG* | SSC | SSC |
|  |  |  | 37 | 115963 | F | 115982 | *rpl32-trnL-UAG* | *rpl32-trnL-UAG* | SSC | SSC |
|  |  |  | 37 | 115963 | F | 116001 | *rpl32-trnL-UAG* | *rpl32-trnL-UAG* | SSC | SSC |
|  |  |  | 40 | 115982 | F | 116001 | *rpl32-trnL-UAG* | *rpl32-trnL-UAG* | SSC | SSC |
|  |  |  | 38 | 45049 | F | 123133 | *ycf3** | *ndhA** | LSC | SSC |
|  |  |  | 40 | 100634 | F | 123131 | *rps7-trnV-GAC* | *ndhA** | IRA | SSC |
|  |  |  | 24 | 111824 | F | 131738 | *ycf1* | *ndhA** | IRA | IRB |
|  |  |  | 33 | 133951 | F | 133982 | *rrn5 -rrn4.5* | *rrn5 -rrn4.5* | IRB | IRB |
|  |  |  | 28 | 150260 | F | 150278 | *ycf2* | *ycf2* | IRB | IRB |
|  |  |  | 34 | 150254 | F | 150272 | *ycf2* | *ycf2* | IRB | IRB |
|  |  |  | 29 | 152675 | F | 152696 | *ycf2* | *ycf2* | IRB | IRB |
|  |  |  | 23 | 9708 | R | 9708 | *trnR-UCU-atpA* | *trnR-UCU-atpA* | LSC | LSC |
|  |  |  | 31 | 13791 | R | 13791 | *atpH-atpI* | *atpH-atpI* | LSC | LSC |
|  |  |  | 24 | 51941 | R | 51941 | *ndhK -ndhC* | *ndhK -ndhC* | LSC | LSC |
|  |  |  | 30 | 65155 | R | 65155 | *petA-psbJ* | *petA-psbJ* | LSC | LSC |
|  |  |  | 25 | 115963 | R | 115963 | *rpl32-trnL-UAG* | *rpl32-trnL-UAG* | SSC | SSC |
|  |  |  | 44 | 115963 | R | 115963 | *rpl32-trnL-UAG* | *rpl32-trnL-UAG* | SSC | SSC |
|  |  |  | 38 | 115969 | R | 116001 | *rpl32-trnL-UAG* | *rpl32-trnL-UAG* | SSC | SSC |
|  |  |  | 32 | 115988 | R | 116007 | *rpl32-trnL-UAG* | *rpl32-trnL-UAG* | SSC | SSC |
|  |  |  | 32 | 116007 | R | 116007 | *rpl32-trnL-UAG* | *rpl32-trnL-UAG* | SSC | SSC |
|  | Palindromic repeats |  | 53 | 9446 | P | 9446 | *trnG-GCC-trnR-UCU* | *trnG-GCC-trnR-UCU* | LSC | LSC |
|  |  |  | 33 | 13086 | P | 13086 | *atpF -atpH* | *atpF -atpH* | LSC | LSC |
|  |  |  | 32 | 13787 | P | 13787 | *atpH-atpI* | *atpH-atpI* | LSC | LSC |
|  |  |  | 30 | 13795 | P | 13795 | *atpH-atpI* | *atpH-atpI* | LSC | LSC |
|  |  |  | 36 | 37706 | P | 37706 | *psbZ -trnG-GCC* | *psbZ -trnG-GCC* | LSC | LSC |
|  |  |  | 30 | 8134 | P | 46541 | *trnS-GCU* | *trnS-GGA* | LSC | LSC |
|  |  |  | 31 | 48204 | P | 48204 | *trnT-UGU-trnL-UAA* | *trnT-UGU-trnL-UAA* | LSC | LSC |
|  |  |  | 43 | 52725 | P | 52725 | *ndhC-trnV-UAC* | *ndhC-trnV-UAC* | LSC | LSC |
|  |  |  | 37 | 69004 | P | 69004 | *psaJ -rpl33* | *psaJ -rpl33* | LSC | LSC |
|  |  |  | 38 | 75963 | P | 75963 | *psbT-psbN* | *psbT-psbN* | LSC | LSC |
|  |  |  | 35 | 45050 | P | 77292 | *ycf3** | *petB** | LSC | LSC |
|  |  |  | 24 | 111824 | P | 111824 | *ycf1* | *ycf1* | IRA | IRA |
|  |  |  | 24 | 131738 | P | 131738 | *ycf1* | *ycf1* | IRB | IRB |
|  |  |  | 33 | 109571 | P | 133951 | *rrn4.5-rrn5* | *rrn5 -rrn4.5* | IRA | IRB |
|  |  |  | 33 | 109602 | P | 133982 | *rrn4.5-rrn5* | *rrn5 -rrn4.5* | IRA | IRB |
|  |  |  | 39 | 45049 | P | 142911 | *ycf3** | *trnV-GAC-rps7* | LSC | IRB |
|  |  |  | 40 | 123131 | P | 142912 | *ndhA** | *trnV-GAC-rps7* | SSC | IRB |
|  |  |  | 34 | 93280 | P | 150254 | *ycf2* | *ycf2* | IRA | IRB |
|  |  |  | 34 | 93298 | P | 150272 | *ycf2* | *ycf2* | IRA | IRB |
|  |  |  | 29 | 90861 | P | 152675 | *ycf2* | *ycf2* | IRA | IRB |
|  |  |  | 29 | 90882 | P | 152696 | *ycf2* | *ycf2* | IRA | IRB |
| *P.* *tangutica* | Dispersed repeats |  | 40 | 40242 | F | 42466 | *psaB* | *psaA* | LSC | LSC |
|  |  |  | 28 | 68 | F | 53038 | *rpl2-trnH-GUG* | *ndhC-trnV-UAC* | LSC | LSC |
|  |  |  | 30 | 53019 | F | 53042 | *ndhC-trnV-UAC* | *ndhC-trnV-UAC* | LSC | LSC |
|  |  |  | 22 | 1687 | F | 84673 | *psbA -trnK-UUU* | *rpl16-rps3* | LSC | LSC |
|  |  |  | 29 | 91132 | F | 91153 | *ycf2* | *ycf2* | IRA | IRA |
|  |  |  | 34 | 93551 | F | 93569 | *ycf2* | *ycf2* | IRA | IRA |
|  |  |  | 39 | 45264 | F | 100908 | *ycf3** | *rps7-trnV-GAC* | LSC | IRA |
|  |  |  | 33 | 109843 | F | 109874 | *rrn4.5-rrn5* | *rrn4.5-rrn5* | IRA | IRA |
|  |  |  | 38 | 45264 | F | 123324 | *ycf3** | *ndhA** | LSC | SSC |
|  |  |  | 40 | 100906 | F | 123322 | *rps7 -trnV-GAC* | *ndhA** | IRA | SSC |
|  |  |  | 23 | 128470 | F | 128491 | *rps15 -ycf1* | *rps15 -ycf1* | SSS | SSC |
|  |  |  | 24 | 112096 | F | 131979 | *ycf1* | *ycf1* | IRA | IRB |
|  |  |  | 33 | 134192 | F | 134223 | *rrn5 -rrn4.5* | *rrn5 -rrn4.5* | IRB | IRB |
|  |  |  | 28 | 150502 | F | 150520 | *ycf2* | *ycf2* | IRB | IRB |
|  |  |  | 34 | 150496 | F | 150514 | *ycf2* | *ycf2* | IRB | IRB |
|  |  |  | 29 | 152917 | F | 152938 | *ycf2* | *ycf2* | IRB | IRB |
|  |  |  | 37 | 21 | F | 157767 | *rpl2-trnH-GUG* | *rpl2-psbA* | LSC | IRB |
|  |  |  | 28 | 53038 | F | 157947 | *ndhC-trnV-UAC* | *rpl2-psbA* | LSC | IRB |
|  |  |  | 124 | 63 | F | 157942 | *rpl2-psbA* | *rpl2-psbA* | LSC | IRB |
|  |  |  | 28 | 48238 | R | 48238 | *trnT-UGU-trnL-UAA* | *trnT-UGU-trnL-UAA* | LSC | LSC |
|  |  |  | 23 | 52169 | R | 52169 | *ndhK-ndhC* | *ndhK-ndhC* | LSC | LSC |
|  |  |  | 36 | 52938 | R | 52944 | *ndhC-trnV-UAC* | *ndhC-trnV-UAC* | LSC | LSC |
|  |  |  | 33 | 58733 | R | 58733 | *rbcL-accD* | *rbcL-accD* | LSC | LSC |
|  |  |  | 26 | 60830 | R | 60830 | *accD-psaI* | *accD-psaI* | LSC | LSC |
|  |  |  | 30 | 65354 | R | 65354 | *petA -psbJ* | *petA -psbJ* | LSC | LSC |
|  |  |  | 29 | 84675 | R | 84675 | *rpl16-rps3* | *rpl16-rps3* | LSC | LSC |
|  |  |  | 27 | 116209 | R | 116209 | *rpl32 -trnL-UAG* | *rpl32 -trnL-UAG* | SSC | SSC |
|  |  |  | 32 | 116216 | R | 116216 | *rpl32 -trnL-UAG* | *rpl32 -trnL-UAG* | SSC | SSC |
|  | Palindromic repeats |  | 53 | 9813 | P | 9813 | *trnG-GCC-trnR-UCU* | *trnG-GCC-trnR-UCU* | LSC | LSC |
|  |  |  | 33 | 13191 | P | 13191 | *atpF-atpH* | *atpF-atpH* | LSC | LSC |
|  |  |  | 38 | 29545 | P | 29545 | *petN-psbM* | *petN-psbM* | LSC | LSC |
|  |  |  | 30 | 8222 | P | 46755 | *psbI-trnS-GCU* | *psaA* | LSC | LSC |
|  |  |  | 31 | 48418 | P | 48418 | *trnT-UGU-trnL-UAA* | *trnT-UGU-trnL-UAA* | LSC | LSC |
|  |  |  | 35 | 69223 | P | 69223 | *psaJ-rpl33* | *psaJ-rpl33* | LSC | LSC |
|  |  |  | 36 | 16634 | P | 72414 | *rps2 -rpoC2* | *clpP** | LSC | LSC |
|  |  |  | 38 | 76196 | P | 76196 | *psbT-psbN* | *psbT-psbN* | LSC | LSC |
|  |  |  | 35 | 45265 | P | 77555 | *ycf3** | *petB** | LSC | LSC |
|  |  |  | 44 | 21 | P | 86288 | *rpl2-trnH-GUG* | *rps19* | LSC | IRA |
|  |  |  | 24 | 112096 | P | 112096 | *ycf1* | *ycf1* | IRA | IRA |
|  |  |  | 24 | 131979 | P | 131979 | *ycf1* | *ycf1* | IRB | IRB |
|  |  |  | 33 | 109843 | P | 134192 | *rrn4.5-rrn5* | *rrn5 -rrn4.5* | IRA | IRB |
|  |  |  | 33 | 109874 | P | 134223 | *rrn4.5-rrn5* | *rrn5 -rrn4.5* | IRA | IRB |
|  |  |  | 39 | 45264 | P | 143152 | *ycf3** | *trnV-GAC-rps7* | LSC | IRB |
|  |  |  | 40 | 123322 | P | 143153 | *ndhA ** | *trnV-GAC-rps7* | SSC | IRB |
|  |  |  | 34 | 93551 | P | 150496 | *ycf2* | *ycf2* | IRA | IRB |
|  |  |  | 34 | 93569 | P | 150514 | *ycf2* | *ycf2* | IRA | IRB |
|  |  |  | 29 | 91132 | P | 152917 | *ycf2* | *ycf2* | IRA | IRB |
|  |  |  | 29 | 91153 | P | 152938 | *ycf2* | *ycf2* | IRA | IRB |
|  |  |  | 147 | 86148 | P | 157811 | *rps19* | *rpl2-psbA* | IRA | IRB |
| *P. triloba* | Dispersed repeats |  | 40 | 40477 | F | 42701 | *psaB* | *psaA* | LSC | LSC |
|  |  |  | 65 | 0 | F | 86584 | *rpl2-trnH-GUG* | *rps19-rpl2* | LSC | IRA |
|  |  |  | 29 | 91337 | F | 91358 | *ycf2* | *ycf2* | IRA | IRA |
|  |  |  | 34 | 93756 | F | 93774 | *ycf2* | *ycf2* | IRA | IRA |
|  |  |  | 39 | 45499 | F | 101113 | *ycf3** | *rps7-trnV-GAC* | LSC | IRA |
|  |  |  | 33 | 110048 | F | 110079 | *rrn4.5-rrn5* | *rrn4.5-rrn5* | IRA | IRA |
|  |  |  | 38 | 45499 | F | 123529 | *ycf3** | *ndhA** | LSC | SSC |
|  |  |  | 40 | 101111 | F | 123527 | *rps7-trnV-GAC* | *ndhA** | IRA | SSC |
|  |  |  | 23 | 128675 | F | 128696 | *rps15-ycf1* | *rps15-ycf1* | SSC | SSC |
|  |  |  | 24 | 112301 | F | 132184 | *ycf1* | *ycf1* | IRA | IRB |
|  |  |  | 33 | 134397 | F | 134428 | *rrn5 -rrn4.5* | *rrn5 -rrn4.5* | IRB | IRB |
|  |  |  | 28 | 150707 | F | 150725 | *ycf2* | *ycf2* | IRB | IRB |
|  |  |  | 34 | 150701 | F | 150719 | *ycf2* | *ycf2* | IRB | IRB |
|  |  |  | 29 | 153122 | F | 153143 | *ycf2* | *ycf2* | IRB | IRB |
|  |  |  | 36 | 29 | F | 157889 | *rpl2-trnH-GUG* | *rpl2-trnH-GUG* | LSC | IRB |
|  |  |  | 48 | 17 | F | 157877 | *rpl2-trnH-GUG* | *rpl2-trnH-GUG* | LSC | IRB |
|  |  |  | 65 | 0 | F | 157860 | *rpl2-trnH-GUG* | *rpl2-trnH-GUG* | LSC | IRB |
|  |  |  | 23 | 52403 | R | 52403 | *ndhK-ndhC* | *ndhK-ndhC* | LSC | LSC |
|  |  |  | 36 | 53172 | R | 53178 | *ndhC-trnV-UAC* | *ndhC-trnV-UAC* | LSC | LSC |
|  |  |  | 33 | 58944 | R | 58944 | *rbcL -accD* | *rbcL -accD* | LSC | LSC |
|  |  |  | 26 | 61042 | R | 61042 | *accD-psaI* | *accD-psaI* | LSC | LSC |
|  |  |  | 30 | 65566 | R | 65566 | *petA-psbJ* | *petA-psbJ* | LSC | LSC |
|  |  |  | 27 | 116414 | R | 116414 | *rpl32 -trnL-UAG* | *rpl32 -trnL-UAG* | SSC | SSC |
|  |  |  | 32 | 116421 | R | 116421 | *rpl32 -trnL-UAG* | *rpl32 -trnL-UAG* | SSC | SSC |
|  | Palindromic repeats |  | 53 | 10022 | P | 10022 | *trnG-GCC-trnR-UCU* | *trnG-GCC-trnR-UCU* | LSC | LSC |
|  |  |  | 33 | 13424 | P | 13424 | *atpF-atpH* | *atpF-atpH* | LSC | LSC |
|  |  |  | 38 | 29780 | P | 29780 | *petN -psbM* | *petN -psbM* | LSC | LSC |
|  |  |  | 30 | 8431 | P | 46990 | *trnS-GCU* | *trnS-GGA* | LSC | LSC |
|  |  |  | 31 | 48653 | P | 48653 | *trnT-UGU-trnL-UAA* | *trnT-UGU-trnL-UAA* | LSC | LSC |
|  |  |  | 35 | 69435 | P | 69435 | *psaJ-rpl33* | *psaJ-rpl33* | LSC | LSC |
|  |  |  | 32 | 16867 | P | 72628 | *rps2-rpoC2* | *clpP** | LSC | LSC |
|  |  |  | 38 | 76430 | P | 76430 | *psbT-psbN* | *psbT-psbN* | LSC | LSC |
|  |  |  | 35 | 45500 | P | 77759 | *ycf3** | *petB** | LSC | LSC |
|  |  |  | 36 | 29 | P | 86584 | *rpl2-trnH-GUG* | *rps19-rpl2* | LSC | IRA |
|  |  |  | 48 | 17 | P | 86584 | *rpl2-trnH-GUG* | *rps19-rpl2* | LSC | IRA |
|  |  |  | 24 | 112301 | P | 112301 | *ycf1* | *ycf1* | IRA | IRA |
|  |  |  | 24 | 132184 | P | 132184 | *ycf1* | *ycf1* | IRB | IRB |
|  |  |  | 33 | 110048 | P | 134397 | *rrn4.5-rrn5* | *rrn5 -rrn4.5* | IRA | IRB |
|  |  |  | 33 | 110079 | P | 134428 | *rrn4.5-rrn5* | *rrn5 -rrn4.5* | IRA | IRB |
|  |  |  | 39 | 45499 | P | 143357 | *ycf3** | *trnV-GAC-rps7* | LSC | IRB |
|  |  |  | 40 | 123527 | P | 143358 | *ndhA ** | *trnV-GAC-rps7* | SSC | IRB |
|  |  |  | 34 | 93756 | P | 150701 | *ycf2* | *ycf2* | IRA | IRB |
|  |  |  | 34 | 93774 | P | 150719 | *ycf2* | *ycf2* | IRA | IRB |
|  |  |  | 29 | 91337 | P | 153122 | *ycf2* | *ycf2* | IRA | IRB |
|  |  |  | 29 | 91358 | P | 153143 | *ycf2* | *ycf2* | IRA | IRB |
| *P. mira* | Dispersed repeats |  | 29 | 42 | F | 71 | *rpl2 -trnH-GUG* | *rpl2 -trnH-GUG* | LSC | LSC |
|  |  |  | 24 | 37247 | F | 37268 | *trnS-UGA-psbZ* | *trnS-UGA-psbZ* | LSC | LSC |
|  |  |  | 40 | 40308 | F | 42532 | *psaB* | *psaA* | LSC | LSC |
|  |  |  | 30 | 53116 | F | 53137 | *ndhC -trnV-UAC* | *ndhC -trnV-UAC* | LSC | LSC |
|  |  |  | 29 | 91165 | F | 91186 | *ycf2* | *ycf2* | IRA | IRA |
|  |  |  | 34 | 93584 | F | 93602 | *ycf2* | *ycf2* | IRA | IRA |
|  |  |  | 44 | 96596 | F | 96629 | *trnL-CAA-ndhB* | *trnL-CAA-ndhB* | IRA | IRA |
|  |  |  | 53 | 96587 | F | 96620 | *trnL-CAA-ndhB* | *trnL-CAA-ndhB* | IRA | IRA |
|  |  |  | 39 | 45337 | F | 100952 | *ycf3** | *rps7-trnV-GAC* | LSC | IRA |
|  |  |  | 33 | 109887 | F | 109918 | *rrn4.5-rrn5* | *rrn4.5-rrn5* | IRA | IRA |
|  |  |  | 47 | 116271 | F | 116295 | *rpl32 -trnL-UAG* | *rpl32 -trnL-UAG* | SSC | SSC |
|  |  |  | 38 | 45337 | F | 123421 | *ycf3** | *trnL-CAA-ndhB* | LSC | SSC |
|  |  |  | 40 | 100950 | F | 123419 | *rps7-trnV-GAC* | *trnL-CAA-ndhB* | IRA | SSC |
|  |  |  | 24 | 112140 | F | 132024 | *ycf1* | *ycf1* | IRA | IRB |
|  |  |  | 33 | 134237 | F | 134268 | *rrn5 -rrn4.5* | *rrn5 -rrn4.5* | IRB | IRB |
|  |  |  | 44 | 147482 | F | 147515 | *ndhB -trnL-CAA* | *ndhB -trnL-CAA* | IRB | IRB |
|  |  |  | 28 | 150546 | F | 150564 | *ycf2* | *ycf2* | IRB | IRB |
|  |  |  | 34 | 150540 | F | 150558 | *ycf2* | *ycf2* | IRB | IRB |
|  |  |  | 29 | 71 | F | 157992 | *rpl2 -trnH-GUG* | *rpl2 -trnH-GUG* | LSC | IRB |
|  |  |  | 29 | 42 | F | 158021 | *rpl2 -trnH-GUG* | *rpl2 -trnH-GUG* | LSC | IRB |
|  |  |  | 29 | 157992 | F | 158021 | *rpl2 -trnH-GUG* | *rpl2 -trnH-GUG* | IRB | IRB |
|  |  |  | 27 | 33778 | R | 33778 | *trnT-GGU -psbD* | *trnT-GGU -psbD* | LSC | LSC |
|  |  |  | 24 | 52233 | R | 52233 | *ndhK-ndhC* | *ndhK-ndhC* | LSC | LSC |
|  |  |  | 32 | 116260 | R | 116260 | *rpl32 -trnL-UAG* | *rpl32 -trnL-UAG* | SSC | SSC |
|  | Palindromic repeats |  | 53 | 9793 | P | 9793 | *trnG-GCC-trnR-UCU* | *trnG-GCC-trnR-UCU* | LSC | LSC |
|  |  |  | 33 | 13214 | P | 13214 | *atpF-atpH* | *atpF-atpH* | LSC | LSC |
|  |  |  | 30 | 8205 | P | 46809 | *trnS-GCU* | *trnS-GGA* | LSC | LSC |
|  |  |  | 31 | 48472 | P | 48472 | *trnT-UGU-trnL-UAA* | *trnT-UGU-trnL-UAA* | LSC | LSC |
|  |  |  | 37 | 69296 | P | 69296 | *psaJ -rpl33* | *psaJ -rpl33* | LSC | LSC |
|  |  |  | 38 | 76288 | P | 76288 | *psbT-psbN* | *psbT-psbN* | LSC | LSC |
|  |  |  | 35 | 45338 | P | 77611 | *ycf3** | *petB** | LSC | LSC |
|  |  |  | 40 | 0 | P | 86186 | *rpl2 -trnH-GUG* | *rps19* | LSC | IRA |
|  |  |  | 24 | 112140 | P | 112140 | *ycf1* | *ycf1* | IRA | IRA |
|  |  |  | 24 | 132024 | P | 132024 | *ycf1* | *ycf1* | IRB | IRB |
|  |  |  | 33 | 109887 | P | 134237 | *rrn4.5-rrn5* | *rrn5 -rrn4.5* | IRA | IRB |
|  |  |  | 33 | 109918 | P | 134268 | *rrn4.5-rrn5* | *rrn5 -rrn4.5* | IRA | IRB |
|  |  |  | 39 | 45337 | P | 143197 | *ycf3** | *trnV-GAC-rps7* | LSC | IRB |
|  |  |  | 40 | 123419 | P | 143198 | *ndhA** | *trnV-GAC-rps7* | SSC | IRB |
|  |  |  | 44 | 96596 | P | 147482 | *trnL-CAA-ndhB* | *ndhB -trnL-CAA* | IRA | IRB |
|  |  |  | 53 | 96587 | P | 147482 | *trnL-CAA-ndhB* | *ndhB -trnL-CAA* | IRA | IRB |
|  |  |  | 44 | 96629 | P | 147515 | *trnL-CAA-ndhB* | *ndhB -trnL-CAA* | IRA | IRB |
|  |  |  | 53 | 96620 | P | 147515 | *trnL-CAA-ndhB* | *ndhB -trnL-CAA* | IRA | IRB |
|  |  |  | 129 | 96546 | P | 147480 | *trnL-CAA-ndhB* | *ndhB -trnL-CAA* | IRA | IRB |
|  |  |  | 34 | 93584 | P | 150540 | *ycf2* | *ycf2* | IRA | IRB |
|  |  |  | 34 | 93602 | P | 150558 | *ycf2* | *ycf2* | IRA | IRB |
|  |  |  | 29 | 91165 | P | 152961 | *ycf2* | *ycf2* | IRA | IRB |
|  |  |  | 29 | 91186 | P | 152982 | *ycf2* | *ycf2* | IRA | IRB |
| *P. tenella* | Dispersed repeats |  | 37 | 253 | F | 290 | *rpl2-trnH-GUG* | *rpl2-trnH-GUG* | LSC | LSC |
|  |  |  | 28 | 251 | F | 311 | *rpl2-trnH-GUG* | *rpl2-trnH-GUG* | LSC | LSC |
|  |  |  | 26 | 290 | F | 313 | *rpl2-trnH-GUG* | *rpl2-trnH-GUG* | LSC | LSC |
|  |  |  | 34 | 13402 | F | 13421 | *atpF-atpH* | *atpF-atpH* | LSC | LSC |
|  |  |  | 103 | 13504 | F | 13615 | *atpF-atpH* | *atpF-atpH* | LSC | LSC |
|  |  |  | 40 | 40623 | F | 42847 | *psaB* | *psaA* | LSC | LSC |
|  |  |  | 41 | 44373 | F | 44427 | *psaA-trnS-GGA* | *psaA-trnS-GGA* | LSC | LSC |
|  |  |  | 66 | 53480 | F | 53544 | *ndhC-trnV-UAC* | *ndhC-trnV-UAC* | LSC | LSC |
|  |  |  | 44 | 72933 | F | 72991 | *clpP** | *clpP** | LSC | LSC |
|  |  |  | 29 | 91588 | F | 91609 | *ycf2* | *ycf2* | IRA | IRA |
|  |  |  | 34 | 94007 | F | 94025 | *ycf2* | *ycf2* | IRA | IRA |
|  |  |  | 39 | 45681 | F | 101378 | *psaA-trnS-GGA* | *rps7 -trnV-GAC* | LSC | IRA |
|  |  |  | 33 | 110313 | F | 110344 | *rrn4.5-rrn5* | *rrn4.5-rrn5* | IRA | IRA |
|  |  |  | 38 | 45681 | F | 123844 | *psaA-trnS-GGA* | *ndhA** | LSC | SSC |
|  |  |  | 40 | 101376 | F | 123842 | *rps7-trnV-GAC* | *ndhA** | IRA | SSC |
|  |  |  | 33 | 128978 | F | 128999 | *ycf1* | *ycf1* | SSC | SSC |
|  |  |  | 24 | 112566 | F | 132466 | *ycf1* | *ycf1* | IRA | IRB |
|  |  |  | 33 | 134679 | F | 134710 | *rrn5 -rrn4.5* | *rrn5 -rrn4.5* | IRB | IRB |
|  |  |  | 28 | 150988 | F | 151006 | *ycf2* | *ycf2* | IRB | IRB |
|  |  |  | 34 | 150982 | F | 151000 | *ycf2* | *ycf2* | IRB | IRB |
|  |  |  | 34 | 13420 | R | 13420 | *atpF-atpH* | *atpF-atpH* | LSC | LSC |
|  |  |  | 31 | 61278 | R | 61278 | *accD -psaI* | *accD -psaI* | LSC | LSC |
|  |  |  | 29 | 65839 | R | 65839 | *petA-psbJ* | *petA-psbJ* | LSC | LSC |
|  |  |  | 25 | 116715 | R | 116715 | *rpl32-trnL-UAG* | *rpl32-trnL-UAG* | SSC | SSC |
|  |  |  | 32 | 116721 | R | 116721 | *rpl32-trnL-UAG* | *rpl32-trnL-UAG* | SSC | SSC |
|  | Palindromic repeats |  | 53 | 10043 | P | 10043 | *trnG-GCC-trnR-UCU* | *trnG-GCC-trnR-UCU* | LSC | LSC |
|  |  |  | 33 | 13516 | P | 13516 | *atpF-atpH* | *atpF-atpH* | LSC | LSC |
|  |  |  | 33 | 13516 | P | 13627 | *atpF-atpH* | *atpF-atpH* | LSC | LSC |
|  |  |  | 33 | 13627 | P | 13627 | *atpF-atpH* | *atpF-atpH* | LSC | LSC |
|  |  |  | 59 | 16968 | P | 16968 | *rps2-rpoC2* | *rps2-rpoC2* | LSC | LSC |
|  |  |  | 30 | 8460 | P | 47171 | *trnS-GCU* | *trnS-GGA* | LSC | LSC |
|  |  |  | 31 | 48843 | P | 48843 | *trnT-UGU -trnL-UAA* | *trnT-UGU -trnL-UAA* | LSC | LSC |
|  |  |  | 35 | 69703 | P | 69703 | *psaJ -rpl33* | *psaJ -rpl33* | LSC | LSC |
|  |  |  | 38 | 76703 | P | 76703 | *psbT-psbN* | *psbT-psbN* | LSC | LSC |
|  |  |  | 35 | 45682 | P | 78025 | *psaA-trnS-GGA* | *psbH-petD* | LSC | LSC |
|  |  |  | 24 | 112566 | P | 112566 | *ycf1* | *ycf1* | IRA | IRA |
|  |  |  | 25 | 84259 | P | 116739 | *rpl16** | *rpl32-trnL-UAG* | LSC | SSC |
|  |  |  | 24 | 132466 | P | 132466 | *ycf1* | *ycf1* | IRB | IRB |
|  |  |  | 33 | 110313 | P | 134679 | *rrn4.5-rrn5* | *rrn5 -rrn4.5* | IRA | IRB |
|  |  |  | 33 | 110344 | P | 134710 | *rrn4.5-rrn5* | *rrn5 -rrn4.5* | IRA | IRB |
|  |  |  | 39 | 45681 | P | 143639 | *psaA-trnS-GGA* | *trnV-GAC -rps7* | LSC | IRB |
|  |  |  | 40 | 123842 | P | 143640 | *ndhA** | *trnV-GAC -rps7* | SSC | IRB |
|  |  |  | 34 | 94007 | P | 150982 | *ycf2* | *ycf2* | IRA | IRB |
|  |  |  | 34 | 94025 | P | 151000 | *ycf2* | *ycf2* | IRA | IRB |
|  |  |  | 29 | 91588 | P | 153403 | *ycf2* | *ycf2* | IRA | IRB |
|  |  |  | 29 | 91609 | P | 153424 | *ycf2* | *ycf2* | IRA | IRB |

Note: * Indicates that the repeat sequence is in the intron; F is Forward; R is Reverse repeat; P is Palindromic repeat.

**Table S2 The tandem repeats distribution in the chloroplast genomes of 12 subg. *Amygdalus* species.**

| **Species Name** | **Indices** | **Size(bp)** | **Copy Number** | **Start Position** | **Location** | **Region** |
| --- | --- | --- | --- | --- | --- | --- |
| *P. dulcis* | 396--438 | 21 | 2 | 396 | *trnH-GUG -psbA* | LSC |
|  | 10320--10433 | 62 | 2 | 10320 | *trnR-UCU-atpA* | LSC |
|  | 29148--29202 | 17 | 3 | 29148 | *trnC-GCA-petN* | LSC |
|  | 29155--29194 | 19 | 2 | 29155 | *trnC-GCA-petN* | LSC |
|  | 34218--34261 | 20 | 2 | 34218 | *trnT-GGU-psbD* | LSC |
|  | 48135--48186 | 24 | 2 | 48135 | *rps4-trnT-UGU* | LSC |
|  | 53335--53414 | 30 | 3 | 53335 | *ndhC -trnV-UAC* | LSC |
|  | 62589--62652 | 32 | 2 | 62589 | *ycf4 -cemA* | LSC |
|  | 86081--86333 | 127 | 2 | 86081 | *rps19-rpl2* | LSC |
|  | 91201--91268 | 21 | 3 | 91201 | *ycf2* | IRA |
|  | 93620--93671 | 18 | 3 | 93620 | *ycf2* | IRA |
|  | 109911--109974 | 31 | 2 | 109911 | *rrn4.5-rrn5* | IRA |
|  | 131561--131811 | 123 | 2 | 131561 | *ycf1* | SSC |
|  | 134318--134381 | 31 | 2 | 134318 | *rrn5-rrn4.5* | IRB |
|  | 150621--150672 | 18 | 3 | 150621 | *ycf2* | IRB |
|  | 153025--153113 | 21 | 4 | 153025 | *ycf2* | IRB |
| *P.* *davidiana* var*. potaninii* Rehd. | 121--191 | 23 | 3 | 121 | *rps19 -trnH-GUG* | LSC |
|  | 151--214 | 23 | 3 | 151 | *rps19 -trnH-GUG* | LSC |
|  | 10164--10221 | 20 | 3 | 10164 | *trnR-UCU-atpA* | LSC |
|  | 29209--29263 | 17 | 3 | 29209 | *trnC-GCA-petN* | LSC |
|  | 29216--29255 | 19 | 2 | 29216 | *trnC-GCA-petN* | LSC |
|  | 34176--34216 | 19 | 2 | 34176 | *trnT-GGU-psbD* | LSC |
|  | 34371--34415 | 22 | 2 | 34371 | *trnT-GGU-psbD* | LSC |
|  | 49844--49891 | 23 | 2 | 49844 | *trnL-UAA** | LSC |
|  | 53515--53555 | 19 | 2 | 53515 | *ndhC-trnV-UAC* | LSC |
|  | 61610--61653 | 21 | 2 | 61610 | *accD-psaI* | LSC |
|  | 91468--91535 | 21 | 3 | 91468 | *ycf2* | IRA |
|  | 93890--93941 | 18 | 3 | 93890 | *ycf2* | IRA |
|  | 110186--110249 | 31 | 2 | 110186 | *rrn4.5-rrn5* | IRA |
|  | 112732--112985 | 127 | 2 | 112732 | *ycf1* | SSC |
|  | 115731--115797 | 29 | 3 | 115731 | *ndhF-rpl32* | SSC |
|  | 134601--134664 | 31 | 2 | 134601 | *rrn5-rrn4.5* | IRB |
|  | 150909--150960 | 18 | 3 | 150909 | *ycf2* | IRB |
|  | 153316--153404 | 21 | 4 | 153316 | *ycf2* | IRB |
| *P.* *davidiana* | 5051--5105 | 24 | 2 | 5051 | *trnK-UUU-rps16* | LSC |
|  | 9992--10057 | 21 | 3 | 9992 | *trnR-UCU-atpA* | LSC |
|  | 29043--29097 | 17 | 3 | 29043 | *trnC-GCA-petN* | LSC |
|  | 29050--29089 | 19 | 2 | 29050 | *trnC-GCA-petN* | LSC |
|  | 38142--38193 | 26 | 2 | 38142 | *psbZ -trnG-GCC* | LSC |
|  | 67203--67243 | 19 | 2 | 67203 | *psbE-petL* | LSC |
|  | 91228--91295 | 21 | 3 | 91228 | *ycf2* | IRA |
|  | 93647--93698 | 18 | 3 | 93647 | *ycf2* | IRA |
|  | 109938--110001 | 31 | 2 | 109938 | *rrn4.5-rrn5* | IRA |
|  | 115372--115418 | 23 | 2 | 115372 | *ndhF-rpl32* | SSC |
|  | 116335--116409 | 6 | 12 | 116335 | *rpl32 -trnL-UAG* | SSC |
|  | 116335--116410 | 19 | 4 | 116335 | *rpl32 -trnL-UAG* | SSC |
|  | 116342--116409 | 13 | 5 | 116342 | *rpl32 -trnL-UAG* | SSC |
|  | 134303--134366 | 31 | 2 | 134303 | *trnR-ACG-rrn5* | IRB |
|  | 153010--153098 | 21 | 4 | 153010 | *ycf2* | IRB |
| *P. ferganensis* | 5263--5309 | 23 | 2 | 5263 | *trnK-UUU-rps16* | LSC |
|  | 7170--7266 | 27 | 4 | 7170 | *rps16 -trnQ-UUG* | LSC |
|  | 8557--9148 | 295 | 2 | 8557 | *trnS-GCU-trnG-GCC* | LSC |
|  | 28350--28668 | 165 | 2 | 28350 | *rpoB-trnC-GCA* | LSC |
|  | 29114--29168 | 17 | 3 | 29114 | *trnC-GCA-petN* | LSC |
|  | 29121--29160 | 19 | 2 | 29121 | *trnC-GCA-petN* | LSC |
|  | 53341--53434 | 23 | 4 | 53341 | *ndhC-trnV-UAC* | LSC |
|  | 53367--53474 | 22 | 5 | 53367 | *ndhC-trnV-UAC* | LSC |
|  | 78577--78617 | 20 | 2 | 78577 | *petB -petD* | LSC |
|  | 91452--91519 | 21 | 3 | 91452 | *ycf2* | IRA |
|  | 93871--93922 | 18 | 3 | 93871 | *ycf2* | IRA |
|  | 110162--110225 | 31 | 2 | 110162 | *rrn4.5-rrn5* | IRA |
|  | 116531--116588 | 19 | 3 | 116531 | *rpl32-trnL-UAG* | SSC |
|  | 121434--121477 | 21 | 2 | 121434 | *ndhE-ndhG* | SSC |
|  | 134499--134562 | 31 | 2 | 134499 | *rrn5-rrn4.5* | IRB |
|  | 150802--150853 | 18 | 3 | 150802 | *ycf2* | IRB |
|  | 153206--153294 | 21 | 4 | 153206 | *ycf2* | IRB |
| *P. kansuensis* | 6951--7047 | 27 | 4 | 6951 | *rps16-trnQ-UUG* | LSC |
|  | 9748--9834 | 26 | 3 | 9748 | *trnR-UCU-atpA* | LSC |
|  | 28664--28718 | 17 | 3 | 28664 | *trnC-GCA-petN* | LSC |
|  | 28671--28710 | 19 | 2 | 28671 | *trnC-GCA-petN* | LSC |
|  | 90734--90801 | 21 | 3 | 90734 | *ycf2* | IRA |
|  | 93159--93210 | 18 | 3 | 93159 | *ycf2* | IRA |
|  | 109450--109513 | 31 | 2 | 109450 | *rrn4.5-rrn5* | IRA |
|  | 114497--114540 | 22 | 2 | 114497 | *ndhF-rpl32* | SSC |
|  | 115652--115702 | 25 | 2 | 115652 | *rpl32-trnL-UAG* | SSC |
|  | 115871--115946 | 19 | 4 | 115871 | *rpl32-trnL-UAG* | SSC |
|  | 115871--115945 | 38 | 2 | 115871 | *rpl32-trnL-UAG* | SSC |
|  | 120827--120870 | 21 | 2 | 120827 | *ndhE-ndhG* | SSC |
|  | 133892--133955 | 31 | 2 | 133892 | *rrn5-rrn4.5* | IRB |
|  | 150195--150246 | 18 | 3 | 150195 | *ycf2* | IRB |
|  | 152605--152693 | 21 | 4 | 152605 | *ycf2* | IRB |
| *P. mira* | 43--100 | 29 | 2 | 43 | *rpl2-trnH-GUG* | LSC |
|  | 28874--28928 | 17 | 3 | 28874 | *trnC-GCA-petN* | LSC |
|  | 28881--28920 | 19 | 2 | 28881 | *trnC-GCA-petN* | LSC |
|  | 37248--37292 | 21 | 2 | 37248 | *trnS-UGA-psbZ* | LSC |
|  | 53096--53216 | 21 | 6 | 53096 | *ndhC -trnV-UAC* | LSC |
|  | 91166--91233 | 21 | 3 | 91166 | *ycf2* | IRA |
|  | 93585--93636 | 18 | 3 | 93585 | *ycf2* | IRA |
|  | 96588--96673 | 33 | 3 | 96588 | *trnL-CAA-ndhB* | IRA |
|  | 96588--96687 | 33 | 3 | 96588 | *trnL-CAA-ndhB* | IRA |
|  | 96590--96673 | 17 | 5 | 96590 | *trnL-CAA-ndhB* | IRA |
|  | 96595--96665 | 16 | 5 | 96595 | *trnL-CAA-ndhB* | IRA |
|  | 109888--109951 | 31 | 2 | 109888 | *rrn4.5-rrn5* | IRA |
|  | 116272--116342 | 24 | 3 | 116272 | *rpl32-trnL-UAG* | SSC |
|  | 134238--134301 | 31 | 2 | 134238 | *rrn5-rrn4.5* | IRB |
|  | 147483--147568 | 33 | 3 | 147483 | *trnL-CAA-ycf15* | IRB |
|  | 147485--147570 | 16 | 5 | 147485 | *trnL-CAA-ycf15* | IRB |
|  | 147491--147578 | 15 | 6 | 147491 | *trnL-CAA-ycf15* | IRB |
|  | 150541--150592 | 18 | 3 | 150541 | *ycf2* | IRB |
|  | 152945--153033 | 21 | 4 | 152945 | *ycf2* | IRB |
|  | 157993--158050 | 29 | 2 | 157993 | *rpl2-trnH-GUG* | IRB |
| *P. mongolica* | 10086--10133 | 23 | 2 | 10086 | *trnR-UCU-* *atpA* | LSC |
|  | 15981--16026 | 23 | 2 | 15981 | *atpI-rps2* | LSC |
|  | 48416--48474 | 29 | 2 | 48416 | *trnT-UGU-trnL-UAA* | LSC |
|  | 77627--77666 | 20 | 2 | 77627 | *petB** | LSC |
|  | 86311--86472 | 81 | 2 | 86311 | *rps19* | LSC |
|  | 91386--91453 | 21 | 3 | 91386 | *ycf2* | IRA |
|  | 93805--93856 | 18 | 3 | 93805 | *ycf2* | IRA |
|  | 110101--110164 | 31 | 2 | 110101 | *rrn4.5-rrn5* | IRA |
|  | 112714--112882 | 83 | 2 | 112714 | *ycf1-ndhF* | IRA |
|  | 116539--116579 | 20 | 2 | 116539 | *rpl32-trnL-UAG* | SSC |
|  | 116571--116643 | 15 | 5 | 116571 | *rpl32-trnL-UAG* | SSC |
|  | 116571--116657 | 30 | 3 | 116571 | *rpl32-trnL-UAG* | SSC |
|  | 128879--128922 | 21 | 2 | 128879 | *ycf1* | SSC |
|  | 134575--134638 | 31 | 2 | 134575 | *rrn5-rrn4.5* | IRB |
|  | 150883--150934 | 18 | 3 | 150883 | *ycf2* | IRB |
|  | 153287--153375 | 21 | 4 | 153287 | *ycf2* | IRB |
|  | 157941--158028 | 87 | 2 | 158028 | *rps19* | IRB |
| *P. pedunculata* | 6933--7003 | 30 | 3 | 6933 | *rps16-trnQ-UUG* | LSC |
|  | 28795--28849 | 17 | 3 | 28795 | *trnC-GCA -petN* | LSC |
|  | 28802--28841 | 19 | 2 | 28802 | *trnC-GCA -petN* | LSC |
|  | 43981--44022 | 21 | 2 | 43981 | *psaA-ycf3* | LSC |
|  | 52884--52939 | 22 | 3 | 52884 | *ndhC-trnV-UAC* | LSC |
|  | 52969--53053 | 32 | 3 | 52969 | *ndhC-trnV-UAC* | LSC |
|  | 52969--53074 | 36 | 3 | 52969 | *ndhC-trnV-UAC* | LSC |
|  | 53036--53079 | 22 | 2 | 53036 | *ndhC-trnV-UAC* | LSC |
|  | 83791--83830 | 20 | 2 | 83791 | *rpl16** | LSC |
|  | 91059--91126 | 21 | 3 | 91059 | *ycf2* | IRA |
|  | 93478--93529 | 18 | 3 | 93478 | *ycf2* | IRA |
|  | 109769--109832 | 31 | 2 | 109769 | *rrn4.5-rrn5* | IRA |
|  | 116132--116180 | 19 | 3 | 116132 | *rpl32-trnL-UAG* | SSC |
|  | 128404--128447 | 21 | 2 | 128404 | *ycf1* | SSC |
|  | 134116--134179 | 31 | 2 | 134116 | *rrn5-rrn4.5* | IRB |
|  | 150419--150470 | 18 | 3 | 150419 | *ycf2* | IRB |
|  | 152823--152911 | 21 | 4 | 152823 | *ycf2* | IRB |
| *P. persica* | 4998--5044 | 23 | 2 | 4998 | *trnK-UUU-rps16* | LSC |
|  | 6905--7001 | 27 | 4 | 6905 | *rps16 -trnQ-UUG* | LSC |
|  | 9702--9788 | 26 | 3 | 9702 | *trnR-UCU-atpA* | LSC |
|  | 28618--28672 | 17 | 3 | 28618 | *trnC-GCA-petN* | LSC |
|  | 28625--28664 | 19 | 2 | 28625 | *trnC-GCA-petN* | LSC |
|  | 52811--52905 | 21 | 5 | 52811 | *ndhC-trnV-UAC* | LSC |
|  | 52864--52947 | 21 | 4 | 52864 | *ndhC-trnV-UAC* | LSC |
|  | 53744--53794 | 16 | 3 | 53744 | *trnV-UAC -trnM-CAU* | LSC |
|  | 77987--78027 | 20 | 2 | 77987 | *petB** | LSC |
|  | 90862--90929 | 21 | 3 | 90862 | *ycf2* | IRA |
|  | 93281--93332 | 18 | 3 | 93281 | *ycf2* | IRA |
|  | 109572--109635 | 31 | 2 | 109572 | *rrn4.5-rrn5* | IRA |
|  | 115745--115795 | 25 | 2 | 115745 | *rpl32-trnL-UAG* | SSC |
|  | 115964--116039 | 19 | 4 | 115964 | *rpl32-trnL-UAG* | SSC |
|  | 120887--120930 | 21 | 2 | 120887 | *ndhE-ndhG* | SSC |
|  | 133952--134015 | 31 | 2 | 133952 | *rrn5 -rrn4.5* | IRB |
|  | 150255--150306 | 18 | 3 | 150255 | *ycf2* | IRB |
|  | 152659--152747 | 21 | 4 | 152659 | *ycf2* | IRB |
| *P. tangutica* | 7014--7084 | 30 | 3 | 7014 | *rps16-trnQ-UUG* | LSC |
|  | 28852--28906 | 17 | 3 | 28852 | *trnC-GCA-petN* | LSC |
|  | 28859--28898 | 19 | 2 | 28859 | *trnC-GCA-petN* | LSC |
|  | 53011--53105 | 23 | 5 | 53011 | *ndhC-trnV-UAC* | LSC |
|  | 53027--53140 | 9 | 13 | 53027 | *ndhC-trnV-UAC* | LSC |
|  | 58726--58766 | 20 | 2 | 58726 | *rbcL -accD* | LSC |
|  | 91133--91200 | 21 | 3 | 91133 | *ycf2* | IRA |
|  | 93552--93603 | 18 | 3 | 93552 | *ycf2* | IRA |
|  | 109844--109907 | 31 | 2 | 109844 | *rrn4.5-rrn5* | IRA |
|  | 128471--128514 | 21 | 2 | 128471 | *rps15 -ycf1* | SSC |
|  | 134193--134256 | 31 | 2 | 134193 | *rrn5-rrn4.5* | IRB |
|  | 150497--150548 | 18 | 3 | 150497 | *ycf2* | IRB |
|  | 152901--152989 | 21 | 4 | 152901 | *ycf2* | IRB |
| *P. triloba* | 7223--7293 | 30 | 3 | 7223 | *rps16-trnQ-UUG* | LSC |
|  | 29087--29141 | 17 | 3 | 29087 | *trnC-GCA-petN* | LSC |
|  | 29094--29133 | 19 | 2 | 29094 | *trnC-GCA-petN* | LSC |
|  | 53244--53351 | 34 | 3 | 53244 | *ndhC-trnV-UAC* | LSC |
|  | 53251--53348 | 51 | 2 | 53251 | *ndhC-trnV-UAC* | LSC |
|  | 53254--53316 | 19 | 3 | 53254 | *ndhC-trnV-UAC* | LSC |
|  | 53264--53353 | 18 | 5 | 53264 | *ndhC-trnV-UAC* | LSC |
|  | 58937--58977 | 20 | 2 | 58937 | *rbcL-accD* | LSC |
|  | 91338--91405 | 21 | 3 | 91338 | *ycf2* | IRA |
|  | 93757--93808 | 18 | 3 | 93757 | *ycf2* | IRA |
|  | 110049--110112 | 31 | 2 | 110049 | *rrn4.5-rrn5* | IRA |
|  | 128676--128719 | 21 | 2 | 128676 | *rps15-ycf1* | SSC |
|  | 134398--134461 | 31 | 2 | 134398 | *rrn5-rrn4.5* | IRB |
|  | 150702--150753 | 18 | 3 | 150702 | *ycf2* | IRB |
|  | 153106--153194 | 21 | 4 | 153106 | *ycf2* | IRB |
| *P. tenella* | 254--327 | 37 | 2 | 254 | *rpl2 -trnH-GUG* | LSC |
|  | 283--339 | 23 | 3 | 283 | *rpl2 -trnH-GUG* | LSC |
|  | 5348--5389 | 20 | 2 | 5348 | *trnK-UUU-rps16* | LSC |
|  | 10303--10347 | 20 | 2 | 10303 | *trnR-UCU-atpA* | LSC |
|  | 13403--13455 | 19 | 3 | 13403 | *atpF-atpH* | LSC |
|  | 13499--13724 | 111 | 2 | 13499 | *atpF-atpH* | LSC |
|  | 29214--29268 | 17 | 3 | 29214 | *trnC-GCA-petN* | LSC |
|  | 29221--29260 | 19 | 2 | 29221 | *trnC-GCA-petN* | LSC |
|  | 44358--44484 | 54 | 2 | 44358 | *psaA-trnS-GGA* | LSC |
|  | 53371--53454 | 20 | 4 | 53371 | *ndhC-trnV-UAC* | LSC |
|  | 53480--53604 | 64 | 2 | 53480 | *ndhC-trnV-UAC* | LSC |
|  | 53483--53602 | 29 | 4 | 53483 | *ndhC-trnV-UAC* | LSC |
|  | 91589--91656 | 21 | 3 | 91589 | *ycf2* | IRA |
|  | 94008--94059 | 18 | 3 | 94008 | *ycf2* | IRA |
|  | 110314--110377 | 31 | 2 | 110314 | *rrn4.5-rrn5* | IRA |
|  | 128989--129032 | 21 | 2 | 128989 | *ycf1* | SSC |
|  | 134680--134743 | 31 | 2 | 134680 | *rrn5-rrn4.5* | IRB |
|  | 150983--151034 | 18 | 3 | 150983 | *ycf2* | IRB |
|  | 153387--153475 | 21 | 4 | 153387 | *ycf2* | IRB |

**Table S3 Simple sequence repeats in the chloroplast genomes of 12 subg. *Amygdalus* species.**

| **Species Name** | **SSR type** | **SSR** | **size** | **start** | **end** | **Location** | **Region** |
| --- | --- | --- | --- | --- | --- | --- | --- |
| *P. dulcis* | p1 | (T)12 | 12 | 1778 | 1789 | *psbA-trnK-UUU* | LSC |
|  | p1 | (A)15 | 15 | 6990 | 7004 | *rps16-trnQ-UUG* | LSC |
|  | p4 | (ATAA)3 | 12 | 8730 | 8741 | *trnS-GCU -trnG-GCC* | LSC |
|  | p1 | (T)10 | 10 | 10067 | 10076 | *trnR-UCU-atpA* | LSC |
|  | p1 | (T)10 | 10 | 14344 | 14353 | *atpH-atpI* | LSC |
|  | p1 | (T)10 | 10 | 15058 | 15067 | *atpH -atpI* | LSC |
|  | c | (T)11ctttg(A)13 | 29 | 16931 | 16959 | *rps2 -rpoC2* | LSC |
|  | p1 | (A)13 | 13 | 27936 | 27948 | *rpoB-trnC-GCA* | LSC |
|  | p2 | (TA)6 | 12 | 30683 | 30694 | *petN-psbM* | LSC |
|  | p4 | (TTTA)3 | 12 | 31314 | 31325 | *psbM -trnD-GUC* | LSC |
|  | p1 | (A)11 | 11 | 31649 | 31659 | *psbM -trnD-GUC* | LSC |
|  | p1 | (T)12 | 12 | 37773 | 37784 | *trnS-UGA-psbZ* | LSC |
|  | p4 | (AAAT)3 | 12 | 48153 | 48164 | *rps4-trnT-UGU* | LSC |
|  | p2 | (AT)6 | 12 | 48571 | 48582 | *trnT-UGU -trnL-UAA* | LSC |
|  | p1 | (A)16 | 16 | 49024 | 49039 | *trnT-UGU -trnL-UAA* | LSC |
|  | p1 | (T)10 | 10 | 52489 | 52498 | *ndhK-ndhC* | LSC |
|  | p1 | (T)13 | 13 | 59199 | 59211 | *rbcL-accD* | LSC |
|  | p1 | (C)12 | 12 | 61109 | 61120 | *accD-psaI* | LSC |
|  | p1 | (T)15 | 15 | 61675 | 61689 | *psaI-ycf4* | LSC |
|  | p1 | (A)10 | 10 | 62786 | 62795 | *ycf4-cemA* | LSC |
|  | p1 | (A)12 | 12 | 65362 | 65373 | *petA-psbJ* | LSC |
|  | p1 | (G)10 | 10 | 66552 | 66561 | *psbE-petL* | LSC |
|  | p1 | (T)10 | 10 | 66994 | 67003 | *psbE-petL* | LSC |
|  | p1 | (A)13 | 13 | 67336 | 67348 | *psbE-petL* | LSC |
|  | c | (T)12agaataacttattct(A)10 | 37 | 69223 | 69259 | *psaJ-rpl33* | LSC |
|  | p1 | (T)15 | 15 | 69517 | 69531 | *psaJ-rpl33* | LSC |
|  | p2 | (AT)6 | 12 | 74090 | 74101 | *clpP-psbB* | LSC |
|  | p1 | (T)13 | 13 | 75911 | 75923 | *psbB -psbT* | LSC |
|  | p1 | (T)11 | 11 | 81414 | 81424 | *rps11-rpl36* | LSC |
|  | c | (T)12caatgcaagggtctaa  aaaaaaagaaatat  tatttgttcaaaacaa  aaaacctgcaa(T)10 | 79 | 83035 | 83113 | *rpl14-rpl16* | LSC |
|  | p1 | (T)10 | 10 | 85424 | 85433 | *rps3 -rpl22* | LSC |
|  | p1 | (G)10 | 10 | 96259 | 96268 | *ycf2-trnL-CAA* | IRA |
|  | p6 | (TTCAAA)3 | 18 | 110270 | 110287 | *rrn5-trnR-ACG* | IRA |
|  | p2 | (TA)6 | 12 | 115258 | 115269 | *ndhF-rpl32* | SSC |
|  | p1 | (A)10 | 10 | 120203 | 120212 | *ndhD-psaC* | SSC |
|  | p6 | (TTTGAA)3 | 18 | 134005 | 134022 | *trnR-ACG-rrn5* | IRB |
|  | p1 | (C)10 | 10 | 148024 | 148033 | *trnL-CAA-ycf2* | IRB |
| *P. davidiana* var*. potaninii* Rehd. | p1 | (A)10 | 10 | 409 | 418 | *trnH-GUG-psbA* | LSC |
|  | p1 | (T)16 | 16 | 1807 | 1822 | *psbA-trnK-UUU* | LSC |
|  | p1 | (T)11 | 11 | 4232 | 4242 | *trnK-UUU-rps16* | LSC |
|  | p1 | (A)14 | 14 | 7018 | 7031 | *rps16-trnQ-UUG* | LSC |
|  | p1 | (A)11 | 11 | 8606 | 8616 | *trnS-GCU-trnG-GCC* | LSC |
|  | p4 | (ATAA)4 | 16 | 8745 | 8760 | *trnS-GCU-trnG-GCC* | LSC |
|  | c | (T)12agatttctatgtcaagaaa  tatattttgaatgacttgaataa  gaaacgctcttattacaca  (TTAAT)3 | 88 | 10089 | 10176 | *trnR-UCU-atpA* | LSC |
|  | p1 | (T)11 | 11 | 13535 | 13545 | *atpF-atpH* | LSC |
|  | p1 | (T)11 | 11 | 15112 | 15122 | *atpH-atpI* | LSC |
|  | c | (T)13ctttg(A)13 | 31 | 16986 | 17016 | *rps2-rpoC2* | LSC |
|  | p1 | (A)11 | 11 | 27998 | 28008 | *rpoB-trnC-GCA* | LSC |
|  | p1 | (T)11 | 11 | 29861 | 29871 | *petN-psbM* | LSC |
|  | p2 | (TA)6 | 12 | 30774 | 30785 | *petN-psbM* | LSC |
|  | p4 | (TTTA)3 | 12 | 31405 | 31416 | *psbM-trnD-GUC* | LSC |
|  | p1 | (T)10 | 10 | 32644 | 32653 | *trnD-GUC -trnY-GUA* | LSC |
|  | p1 | (T)12 | 12 | 37398 | 37409 | *psbC-trnS-UGA* | LSC |
|  | p1 | (T)10 | 10 | 37888 | 37897 | *trnS-UGA-psbZ* | LSC |
|  | p2 | (AT)6 | 12 | 48667 | 48678 | *rps4 -trnT-UGU* | LSC |
|  | p1 | (T)10 | 10 | 48789 | 48798 | *trnT-UGU-trnL-UAA* | LSC |
|  | p1 | (A)16 | 16 | 49122 | 49137 | *trnT-UGU-trnL-UAA* | LSC |
|  | p2 | (TA)6 | 12 | 58979 | 58990 | *rbcL-accD* | LSC |
|  | p1 | (T)10 | 10 | 59099 | 59108 | *rbcL-accD* | LSC |
|  | p1 | (T)12 | 12 | 59325 | 59336 | *rbcL-accD* | LSC |
|  | p1 | (C)15 | 15 | 61235 | 61249 | *accD-psaI* | LSC |
|  | p1 | (T)12 | 12 | 61841 | 61852 | *psaI-ycf4* | LSC |
|  | p1 | (T)11 | 11 | 63076 | 63086 | *ycf4-cemA* | LSC |
|  | p1 | (A)10 | 10 | 65808 | 65817 | *petA-psbJ* | LSC |
|  | p1 | (G)13 | 13 | 66994 | 67006 | *psbE-petL* | LSC |
|  | p1 | (T)13 | 13 | 67439 | 67451 | *psbE-petL* | LSC |
|  | p1 | (A)20 | 20 | 67784 | 67803 | *psbE-petL* | LSC |
|  | c | (T)12agaataacttattct(A)11 | 38 | 69669 | 69706 | *psaJ-rpl33* | LSC |
|  | p1 | (T)14 | 14 | 69968 | 69981 | *psaJ-rpl33* | LSC |
|  | p2 | (AT)6 | 12 | 74517 | 74528 | *clpP-psbB* | LSC |
|  | p1 | (T)11 | 11 | 83460 | 83470 | *rpl14-rpl16* | LSC |
|  | p1 | (T)12 | 12 | 85028 | 85039 | *rpl16-rps3* | LSC |
|  | p1 | (T)10 | 10 | 85825 | 85834 | *rps3-rpl22* | LSC |
|  | p1 | (G)11 | 11 | 96529 | 96539 | *ycf2-trnL-CAA* | IRA |
|  | p1 | (A)10 | 10 | 115206 | 115215 | *ndhF-rpl32* | SSC |
|  | p2 | (AT)6 | 12 | 115748 | 115759 | *ndhF-rpl32* | SSC |
|  | p1 | (A)11 | 11 | 120617 | 120627 | *ndhD-psaC* | SSC |
|  | c | (A)10tgaaccttagtattattta  ttagtaaagtaatagtcttagta  aagtaattataaattattcttt  aatcaagagatttac(T)11 | 100 | 122568 | 122667 | *ndhG-ndhI* | SSC |
|  | p1 | (C)11 | 11 | 148311 | 148321 | *trnL-CAA-ycf2* | IRB |
| *P. davidiana* | p1 | (T)16 | 16 | 1620 | 1635 | *psbA* | LSC |
|  | p1 | (A)11 | 11 | 2935 | 2945 | *matK* | LSC |
|  | p1 | (T)11 | 11 | 3281 | 3291 | *matK* | LSC |
|  | p1 | (T)11 | 11 | 4043 | 4053 | *trnK-UUU ** | LSC |
|  | c | (AAAT)3taattaaattattgat  tttaagtacttttttcttattcttccc  caaccg(A)16 | 77 | 5585 | 5661 | *rps16** | LSC |
|  | p5 | (TTTGA)3 | 15 | 6000 | 6014 | *rps16** | LSC |
|  | p1 | (A)14 | 14 | 6853 | 6866 | *rps16-trnQ-UUG* | LSC |
|  | p1 | (A)10 | 10 | 7697 | 7706 | *psbK-psbI* | LSC |
|  | p1 | (A)13 | 13 | 8448 | 8460 | *trnS-GCU-trnG-GCC* | LSC |
|  | p4 | (ATAA)3 | 12 | 8588 | 8599 | *trnS-GCU-trnG-GCC* | LSC |
|  | p1 | (T)10 | 10 | 9520 | 9529 | *trnG-GCC** | LSC |
|  | c | (T)11agatttctatgtcaag  aaatatattttgaatgacttgaa  taagaaacgctcttattacata  (TTAAT)3 | 87 | 9928 | 10014 | *trnR-UCU-atpA* | LSC |
|  | p1 | (A)11 | 11 | 12788 | 12798 | *atpF** | LSC |
|  | p2 | (AT)6 | 12 | 12984 | 12995 | *atpF** | LSC |
|  | p1 | (T)11 | 11 | 14946 | 14956 | *atpH-atpI* | LSC |
|  | c | (T)12ctttg(A)13 | 30 | 16819 | 16848 | *rps2-rpoC2* | LSC |
|  | p1 | (T)11 | 11 | 19039 | 19049 | *rpoC2* | LSC |
|  | p1 | (T)10 | 10 | 26750 | 26759 | *rpoB* | LSC |
|  | p1 | (A)12 | 12 | 27831 | 27842 | *rpoB -trnC-GCA* | LSC |
|  | p1 | (T)11 | 11 | 29689 | 29699 | *petN-psbM* | LSC |
|  | p4 | (TTTA)3 | 12 | 31215 | 31226 | *psbM-trnD-GUC* | LSC |
|  | p1 | (T)11 | 11 | 37166 | 37176 | *psbC-trnS-UGA* | LSC |
|  | p1 | (T)11 | 11 | 37655 | 37665 | *trnS-UGA - psbZ* | LSC |
|  | p5 | (AAATA)3 | 15 | 38161 | 38175 | *psbZ-trnG-GCC* | LSC |
|  | p4 | (TTTC)3 | 12 | 45184 | 45195 | *ycf3** | LSC |
|  | p2 | (AT)6 | 12 | 48478 | 48489 | *trnT-UGUtrnL-UAA* | LSC |
|  | p1 | (A)16 | 16 | 48935 | 48950 | *trnT-UGUtrnL-UAA* | LSC |
|  | c | (TA)6tttttttc(ATATT)3 | 35 | 50770 | 50804 | *trnF-GAA -ndhJ* | LSC |
|  | p1 | (T)10 | 10 | 56389 | 56398 | *atpB* | LSC |
|  | p1 | (T)10 | 10 | 58863 | 58872 | *rbcL-accD* | LSC |
|  | p1 | (T)13 | 13 | 59089 | 59101 | *rbcL-accD* | LSC |
|  | p1 | (C)13 | 13 | 61000 | 61012 | *accD -psaI* | LSC |
|  | p1 | (T)10 | 10 | 61583 | 61592 | *psaI-ycf4* | LSC |
|  | p1 | (T)11 | 11 | 62810 | 62820 | *ycf4-cemA* | LSC |
|  | p1 | (G)12 | 12 | 66728 | 66739 | *psbE* | LSC |
|  | p1 | (T)12 | 12 | 67172 | 67183 | *psbE-petL* | LSC |
|  | p1 | (A)17 | 17 | 67535 | 67551 | *psbE-petL* | LSC |
|  | c | (T)12agaataacttattct  (A)10 | 37 | 69417 | 69453 | *psaJ-rpl33* | LSC |
|  | p1 | (T)14 | 14 | 69711 | 69724 | *psaJ-rpl33* | LSC |
|  | p1 | (A)13 | 13 | 70444 | 70456 | *rps18* | LSC |
|  | p4 | (TAAA)3 | 12 | 72464 | 72475 | *clpP** | LSC |
|  | p1 | (T)14 | 14 | 73607 | 73620 | *clpP** | LSC |
|  | p2 | (AT)6 | 12 | 74259 | 74270 | *clpP-psbB* | LSC |
|  | p1 | (T)11 | 11 | 77194 | 77204 | *petB ** | LSC |
|  | p1 | (T)10 | 10 | 83278 | 83287 | *rpl14-rpl16* | LSC |
|  | p1 | (A)11 | 11 | 84036 | 84046 | *rpl16** | LSC |
|  | p1 | (T)17 | 17 | 84775 | 84791 | *rpl16-rps3* | LSC |
|  | p1 | (T)10 | 10 | 85577 | 85586 | *rps3-rpl22* | LSC |
|  | p1 | (G)10 | 10 | 96286 | 96295 | *ycf2-trnL-CAA* | IRA |
|  | p1 | (A)10 | 10 | 114849 | 114858 | *ndhF-rpl32* | SSC |
|  | p1 | (A)11 | 11 | 120297 | 120307 | *ndhD-psaC* | SSC |
|  | p4 | (TTGA)3 | 12 | 120860 | 120871 | *ndhE* | SSC |
|  | c | (A)11tgaaccttagta  ttatttattagtaaagtaata  gtcttagtaaagtaattata  aattattctttaatcaa  gagatttac(T)14 | 104 | 122248 | 122351 | *ndhG-ndhI* | SSC |
|  | p1 | (T)11 | 11 | 129833 | 129843 | *ycf1* | SSC |
|  | p1 | (A)13 | 13 | 130443 | 130455 | *ycf1* | SSC |
|  | p1 | (T)10 | 10 | 130827 | 130836 | *ycf1* | SSC |
|  | p1 | (C)10 | 10 | 148009 | 148018 | *trnL-CAA-ycf2* | IRB |
| *P. ferganensis* | p1 | (T)14 | 14 | 1856 | 1869 | *psbA -trnK-UUU* | LSC |
|  | p1 | (A)11 | 11 | 3170 | 3180 | *matK* | LSC |
|  | p1 | (T)11 | 11 | 3516 | 3526 | *matK* | LSC |
|  | p1 | (A)10 | 10 | 4007 | 4016 | *trnK-UUU** | LSC |
|  | p1 | (T)12 | 12 | 4279 | 4290 | *trnK-UUU ** | LSC |
|  | c | (C)10tagaaacgtat  aagaagttttctcct  cgtacggctcaaga  aaattataaattatgt  ctatagtatagaatt  ataacatcaaacata  tccata(AAAT)3taa  ttaaattattgattt  taagtactttt  ttcttattcttcccc  aaccg(A)14 | 177 | 5722 | 5898 | *rps16** | LSC |
|  | p5 | (TTTGA)3 | 15 | 6242 | 6256 | *rps16** | LSC |
|  | c | (A)11gaatcacattcta  taccaatattatacc  cttaaacggaagactgttc  gaaaagacaatctt  taatttttatatacttt  attatgatatag(AT)6 | 113 | 7092 | 7204 | *rps16-trnQ-UUG* | LSC |
|  | p1 | (A)10 | 10 | 8712 | 8721 | *trnS-GCU-trnG-GCC* | LSC |
|  | p1 | (A)10 | 10 | 9007 | 9016 | *trnS-GCU-trnG-GCC* | LSC |
|  | c | (T)12agatttctat  gtcaagaaatatattt  tgaatgacttga  ataagaaacgctcttatt  acata(TTAAT)3 | 88 | 10197 | 10284 | *trnR-UCU-atpA* | LSC |
|  | p1 | (T)10 | 10 | 11943 | 11952 | *atpA-atpF* | LSC |
|  | p1 | (A)12 | 12 | 12828 | 12839 | *atpF** | LSC |
|  | p1 | (T)10 | 10 | 15001 | 15010 | *atpH-atpI* | LSC |
|  | c | (T)12ctttg(A)11 | 28 | 16879 | 16906 | *rps2 -rpoC2* | LSC |
|  | p1 | (T)11 | 11 | 19086 | 19096 | *rpoC2* | LSC |
|  | p1 | (T)15 | 15 | 23208 | 23222 | *rpoC1** | LSC |
|  | p1 | (T)10 | 10 | 26814 | 26823 | *rpoB* | LSC |
|  | p1 | (A)11 | 11 | 27894 | 27904 | *rpoB-trnC-GCA* | LSC |
|  | p1 | (T)11 | 11 | 29755 | 29765 | *petN-psbM* | LSC |
|  | p2 | (TA)6 | 12 | 30652 | 30663 | *petN-psbM* | LSC |
|  | p4 | (TTTA)3 | 12 | 31283 | 31294 | *psbM-trnD-GUC* | LSC |
|  | p1 | (C)10 | 10 | 34312 | 34321 | *trnT-GGU-psbD* | LSC |
|  | p1 | (T)19 | 19 | 37245 | 37263 | *psbC-trnS-UGA* | LSC |
|  | p1 | (T)10 | 10 | 37742 | 37751 | *trnS-UGA-psbZ* | LSC |
|  | p1 | (T)10 | 10 | 44674 | 44683 | *ycf3** | LSC |
|  | p4 | (TTTC)3 | 12 | 45242 | 45253 | *ycf3** | LSC |
|  | p1 | (A)13 | 13 | 46217 | 46229 | *ycf3** | LSC |
|  | p2 | (AT)6 | 12 | 48541 | 48552 | *trnT-UGU-trnL-UAA* | LSC |
|  | p1 | (A)15 | 15 | 48995 | 49009 | *trnT-UGU-trnL-UAA* | LSC |
|  | p1 | (T)10 | 10 | 52452 | 52461 | *ndhK -ndhC* | LSC |
|  | p1 | (T)10 | 10 | 56523 | 56532 | *atpB* | LSC |
|  | p1 | (T)16 | 16 | 59222 | 59237 | *rbcL -accD* | LSC |
|  | p1 | (C)11 | 11 | 61136 | 61146 | *accD-psaI* | LSC |
|  | p1 | (T)13 | 13 | 61717 | 61729 | *psaI -ycf4* | LSC |
|  | p1 | (T)12 | 12 | 62823 | 62834 | *ycf4-cemA* | LSC |
|  | p1 | (T)11 | 11 | 62946 | 62956 | *ycf4-cemA* | LSC |
|  | p1 | (A)10 | 10 | 63085 | 63094 | *ycf4-cemA* | LSC |
|  | p1 | (A)12 | 12 | 65662 | 65673 | *petA-psbJ* | LSC |
|  | p1 | (G)10 | 10 | 66873 | 66882 | *psbE-petL* | LSC |
|  | p1 | (A)12 | 12 | 67668 | 67679 | *psbE-petL* | LSC |
|  | c | (T)11agaataagtt  attct(A)13 | 39 | 69554 | 69592 | *psaJ -rpl33* | LSC |
|  | p1 | (T)15 | 15 | 69851 | 69865 | *psaJ -rpl33* | LSC |
|  | p1 | (A)13 | 13 | 70585 | 70597 | *rps18 -rpl20* | LSC |
|  | p1 | (A)12 | 12 | 71288 | 71299 | *rpl20-clpP* | LSC |
|  | p4 | (AAAT)3 | 12 | 72609 | 72620 | *clpP** | LSC |
|  | p1 | (A)11 | 11 | 73449 | 73459 | *clpP** | LSC |
|  | p1 | (A)11 | 11 | 73581 | 73591 | *clpP** | LSC |
|  | p1 | (T)17 | 17 | 73797 | 73813 | *clpP** | LSC |
|  | p2 | (AT)6 | 12 | 74452 | 74463 | *clpP-psbB* | LSC |
|  | p1 | (T)10 | 10 | 77388 | 77397 | *petB** | LSC |
|  | p1 | (T)11 | 11 | 85005 | 85015 | *rpl16-rps3* | LSC |
|  | p1 | (T)10 | 10 | 85801 | 85810 | *rpl22* | LSC |
|  | p1 | (G)10 | 10 | 96510 | 96519 | *trnL-CAA* | IRA |
|  | p1 | (A)10 | 10 | 112846 | 112855 | *ndhF* | SSC |
|  | p1 | (T)10 | 10 | 115914 | 115923 | *ndhF-rpl32* | SSC |
|  | p4 | (TTGA)3 | 12 | 121035 | 121046 | *ndhE* | SSC |
|  | p1 | (A)10 | 10 | 121400 | 121409 | *ndhE-ndhG* | SSC |
|  | p1 | (T)10 | 10 | 130036 | 130045 | *ycf1* | SSC |
|  | p1 | (A)13 | 13 | 130645 | 130657 | *ycf1* | SSC |
|  | p1 | (T)10 | 10 | 131029 | 131038 | *ycf1* | SSC |
|  | p1 | (C)10 | 10 | 148205 | 148214 | *trnL-CAA-ycf15* | IRB |
| *P. kansuensis* | p1 | (T)13 | 13 | 1663 | 1675 | *psbA-trnK-UUU* | LSC |
|  | c | (A)12gaatcacattcta  taccaatattataccctt  aaacggaagactgttc  gaaaagacaatctttaattt  ttatatactttattatgatatag(AT)6 | 114 | 6872 | 6985 | *rps16-trnQ-UUG* | LSC |
|  | p1 | (A)10 | 10 | 8493 | 8502 | *trnS-GCU -trnG-GCC* | LSC |
|  | c | (T)12agatttctatgtc  agaaatatattttgaatgac  ttgaataagaaacgct  cttattacata(TTAAT)4 | 93 | 9683 | 9775 | *trnR-UCU -atpA* | LSC |
|  | p1 | (T)11 | 11 | 14001 | 14011 | *atpH-atpI* | LSC |
|  | p1 | (T)10 | 10 | 14722 | 14731 | *atpH-atpI* | LSC |
|  | p1 | (A)10 | 10 | 16614 | 16623 | *rps2 -rpoC2* | LSC |
|  | p1 | (A)11 | 11 | 27609 | 27619 | *rpoB -trnC-GCA* | LSC |
|  | p1 | (T)11 | 11 | 29299 | 29309 | *petN-psbM* | LSC |
|  | p2 | (TA)6 | 12 | 30190 | 30201 | *petN-psbM* | LSC |
|  | p4 | (TTTA)3 | 12 | 30821 | 30832 | *psbM-trnD-GUC* | LSC |
|  | p5 | (AAAAG)3 | 15 | 31644 | 31658 | *psbM-trnD-GUC* | LSC |
|  | p1 | (T)17 | 17 | 36782 | 36798 | *psbC-trnS-UGA* | LSC |
|  | p2 | (AT)6 | 12 | 48071 | 48082 | *trnT-UGU-trnL-UAA* | LSC |
|  | p1 | (A)13 | 13 | 48525 | 48537 | *trnT-UGU-trnL-UAA* | LSC |
|  | p2 | (TA)7 | 14 | 50363 | 50376 | *trnF-GAA -ndhJ* | LSC |
|  | p1 | (T)10 | 10 | 51998 | 52007 | *ndhK-ndhC* | LSC |
|  | p1 | (T)10 | 10 | 58737 | 58746 | *rbcL-accD* | LSC |
|  | p1 | (C)10 | 10 | 60653 | 60662 | *accD-psaI* | LSC |
|  | p1 | (T)13 | 13 | 61233 | 61245 | *psaI -ycf4* | LSC |
|  | p1 | (T)11 | 11 | 62339 | 62349 | *ycf4-cemA* | LSC |
|  | p1 | (A)10 | 10 | 62478 | 62487 | *ycf4-cemA* | LSC |
|  | p1 | (A)12 | 12 | 65055 | 65066 | *petA-psbJ* | LSC |
|  | p1 | (A)11 | 11 | 67008 | 67018 | *psbE-petL* | LSC |
|  | c | (T)11agaataacttat  tct(A)13 | 39 | 68893 | 68931 | *psaJ-rpl33* | LSC |
|  | p1 | (T)15 | 15 | 69189 | 69203 | *psaJ-rpl33* | LSC |
|  | p1 | (A)11 | 11 | 70626 | 70636 | *rpl20-rps12* | LSC |
|  | p2 | (AT)6 | 12 | 73750 | 73761 | *clpP-psbB* | LSC |
|  | p1 | (T)13 | 13 | 84285 | 84297 | *rpl16 -rps3* | LSC |
|  | p1 | (T)10 | 10 | 85083 | 85092 | *rps3 -rpl22* | LSC |
|  | p1 | (G)10 | 10 | 95798 | 95807 | *ycf2-trnL-CAA* | IRA |
|  | p1 | (T)11 | 11 | 116214 | 116224 | *rpl32-trnL-UAG* | SSC |
|  | p1 | (C)10 | 10 | 147598 | 147607 | *trnL-CAA-ycf2* | IRB |
| *P. mira* | p1 | (T)12 | 12 | 1688 | 1699 | *psbA-trnK-UUU* | LSC |
|  | p1 | (A)11 | 11 | 3008 | 3018 | *matK* | LSC |
|  | p1 | (T)11 | 11 | 3354 | 3364 | *matK* | LSC |
|  | p1 | (A)11 | 11 | 3845 | 3855 | *trnK-UUU** | LSC |
|  | c | (AAAT)3taattaaattatt  gattttaagtacttttttcttat  tcttccccaaccg(A)16 | 77 | 5635 | 5711 | *rps16** | LSC |
|  | p5 | (TTTGA)3 | 15 | 6050 | 6064 | *rps16** | LSC |
|  | p1 | (A)17 | 17 | 6903 | 6919 | *rps16-trnQ-UUG* | LSC |
|  | p4 | (ATAA)4 | 16 | 8654 | 8669 | *trnS-GCU-trnG-GCC* | LSC |
|  | p1 | (T)11 | 11 | 9984 | 9994 | *trnR-UCU-atpA* | LSC |
|  | p1 | (A)11 | 11 | 12626 | 12636 | *atpF** | LSC |
|  | p2 | (AT)6 | 12 | 12822 | 12833 | *atpF** | LSC |
|  | c | (T)10ctttg(A)12 | 27 | 16654 | 16680 | *rps2-rpoC2* | LSC |
|  | p1 | (T)11 | 11 | 18860 | 18870 | *rpoC2* | LSC |
|  | p1 | (T)10 | 10 | 22982 | 22991 | *rpoC1** | LSC |
|  | p1 | (T)10 | 10 | 26583 | 26592 | *rpoB* | LSC |
|  | p1 | (A)12 | 12 | 27663 | 27674 | *rpoB-trnC-GCA* | LSC |
|  | p1 | (T)10 | 10 | 29520 | 29529 | *petN-psbM* | LSC |
|  | p2 | (TA)6 | 12 | 30416 | 30427 | *petN-psbM* | LSC |
|  | p4 | (TTTA)3 | 12 | 31051 | 31062 | *psbM-trnD-GUC* | LSC |
|  | p1 | (T)10 | 10 | 37016 | 37025 | *psbC-trnS-UGA* | LSC |
|  | p1 | (T)10 | 10 | 37525 | 37534 | *trnS-UGA-psbZ* | LSC |
|  | p4 | (TTTC)3 | 12 | 45027 | 45038 | *ycf3 ** | LSC |
|  | p1 | (A)14 | 14 | 45981 | 45994 | *ycf3 ** | LSC |
|  | p1 | (A)15 | 15 | 48766 | 48780 | *trnT-UGU-trnL-UAA* | LSC |
|  | p2 | (TA)7 | 14 | 50607 | 50620 | *trnF-GAA-ndhJ* | LSC |
|  | p1 | (T)10 | 10 | 50891 | 50900 | *trnF-GAA-ndhJ* | LSC |
|  | p1 | (T)10 | 10 | 52241 | 52250 | *ndhK-ndhC* | LSC |
|  | p1 | (T)10 | 10 | 56300 | 56309 | *atpB* | LSC |
|  | p1 | (T)15 | 15 | 59001 | 59015 | *rbcL-accD* | LSC |
|  | p1 | (C)13 | 13 | 60914 | 60926 | *accD-psaI* | LSC |
|  | p1 | (T)12 | 12 | 61507 | 61518 | *psaI-ycf4* | LSC |
|  | p1 | (T)11 | 11 | 62731 | 62741 | *ycf4-cemA* | LSC |
|  | p1 | (A)11 | 11 | 65446 | 65456 | *petA-psbJ* | LSC |
|  | p1 | (G)10 | 10 | 66635 | 66644 | *psbE-petL* | LSC |
|  | p1 | (A)14 | 14 | 67418 | 67431 | *psbE-petL* | LSC |
|  | c | (T)11agaataacttattct(A)12 | 38 | 69297 | 69334 | *psaJ-rpl33* | LSC |
|  | p1 | (T)17 | 17 | 69593 | 69609 | *psaJ-rpl33* | LSC |
|  | p1 | (A)13 | 13 | 70329 | 70341 | *rpl20-rps12* | LSC |
|  | p1 | (A)11 | 11 | 71032 | 71042 | *rpl20-rps12* | LSC |
|  | p4 | (TAAA)3 | 12 | 72351 | 72362 | *clpP** | LSC |
|  | p1 | (T)13 | 13 | 73536 | 73548 | *clpP** | LSC |
|  | p2 | (AT)6 | 12 | 74187 | 74198 | *clpP-psbB* | LSC |
|  | p1 | (T)11 | 11 | 77122 | 77132 | *petB** | LSC |
|  | p1 | (A)10 | 10 | 79916 | 79925 | *petD-rpoA* | LSC |
|  | p6 | (ACTATA)3 | 18 | 82713 | 82730 | *rps8-rpl14* | LSC |
|  | p1 | (T)13 | 13 | 84717 | 84729 | *rpl16-rps3* | LSC |
|  | p1 | (T)10 | 10 | 85515 | 85524 | *rps3-rpl22* | LSC |
|  | p1 | (G)10 | 10 | 96224 | 96233 | *ycf15-trnL-CAA* | IRA |
|  | p1 | (A)10 | 10 | 114799 | 114808 | *ndhF-rpl32* | SSC |
|  | p4 | (TTGA)3 | 12 | 120797 | 120808 | *ndhE* | SSC |
|  | p1 | (T)10 | 10 | 122274 | 122283 | *ndhI* | SSC |
|  | p1 | (T)10 | 10 | 129775 | 129784 | *ycf1* | SSC |
|  | p1 | (A)13 | 13 | 130384 | 130396 | *ycf1* | SSC |
|  | p1 | (T)10 | 10 | 130768 | 130777 | *ycf1* | SSC |
|  | p1 | (C)10 | 10 | 147944 | 147953 | *trnL-CAA-ycf15* | IRB |
| *P.* *mongolica* | p1 | (T)15 | 15 | 1610 | 1624 | *psbA-trnK-UUU* | LSC |
|  | p1 | (A)11 | 11 | 2924 | 2934 | *matK* | LSC |
|  | p1 | (T)11 | 11 | 3270 | 3280 | *matK* | LSC |
|  | p1 | (A)10 | 10 | 3761 | 3770 | *trnK-UUU** | LSC |
|  | c | (AAAT)3taattaaattatt  gattttaagtacttttttcttatt  cttccccaaccg(A)15 | 76 | 5562 | 5637 | *rps16** | LSC |
|  | p5 | (TTTGA)3 | 15 | 5976 | 5990 | *rps16** | LSC |
|  | p1 | (A)15 | 15 | 6829 | 6843 | *rps16-trnQ-UUG* | LSC |
|  | p1 | (A)12 | 12 | 8433 | 8444 | *trnS-GCU-trnG-GCC* | LSC |
|  | p4 | (ATAA)4 | 16 | 8572 | 8587 | *trnS-GCU-trnG-GCC* | LSC |
|  | p1 | (T)10 | 10 | 9508 | 9517 | *trnG-GCC ** | LSC |
|  | p1 | (T)11 | 11 | 9916 | 9926 | *trnR-UCU-atpA* | LSC |
|  | p1 | (A)11 | 11 | 12770 | 12780 | *atpF** | LSC |
|  | p2 | (AT)6 | 12 | 12966 | 12977 | *atpF** | LSC |
|  | p1 | (A)10 | 10 | 13933 | 13942 | *atpH-atpI* | LSC |
|  | p5 | (TTTAT)3 | 15 | 14812 | 14826 | *atpH-atpI* | LSC |
|  | p1 | (T)11 | 11 | 14936 | 14946 | *atpH-atpI* | LSC |
|  | p1 | (A)10 | 10 | 15089 | 15098 | *atpH-atpI* | LSC |
|  | c | (T)12caaag(A)12 | 29 | 16834 | 16862 | *rps2-rpoC2* | LSC |
|  | p1 | (T)11 | 11 | 19053 | 19063 | *rpoC2* | LSC |
|  | p1 | (T)10 | 10 | 26764 | 26773 | *rpoB* | LSC |
|  | p1 | (A)11 | 11 | 27845 | 27855 | *rpoB-trnC-GCA* | LSC |
|  | p1 | (T)11 | 11 | 29684 | 29694 | *petN-psbM* | LSC |
|  | p4 | (TTTA)3 | 12 | 31210 | 31221 | *psbM-trnD-GUC* | LSC |
|  | p1 | (T)12 | 12 | 37161 | 37172 | *psbC-trnS-UGA* | LSC |
|  | p4 | (TTTC)3 | 12 | 45143 | 45154 | *ycf3 ** | LSC |
|  | c | (AT)6aaag(AT)6 | 28 | 48448 | 48475 | *trnT-UGU-trnL-UAA* | LSC |
|  | p1 | (A)28 | 28 | 48918 | 48945 | *trnT-UGU-trnL-UAA* | LSC |
|  | p2 | (TA)12 | 24 | 50781 | 50804 | *trnF-GAA -ndhJ* | LSC |
|  | p1 | (T)10 | 10 | 51081 | 51090 | *trnF-GAA -ndhJ* | LSC |
|  | p1 | (T)10 | 10 | 56418 | 56427 | *atpB* | LSC |
|  | p1 | (T)13 | 13 | 59117 | 59129 | *rbcL-accD* | LSC |
|  | p1 | (C)12 | 12 | 61028 | 61039 | *accD-psaI* | LSC |
|  | p1 | (T)12 | 12 | 61625 | 61636 | *psaI-ycf4* | LSC |
|  | p1 | (T)11 | 11 | 62853 | 62863 | *ycf4 -cemA* | LSC |
|  | p1 | (A)10 | 10 | 62992 | 63001 | *ycf4 -cemA* | LSC |
|  | p1 | (A)12 | 12 | 65588 | 65599 | *petA-psbJ* | LSC |
|  | p1 | (G)13 | 13 | 66776 | 66788 | *psbE-petL* | LSC |
|  | p1 | (T)11 | 11 | 67221 | 67231 | *psbE-petL* | LSC |
|  | p1 | (A)14 | 14 | 67564 | 67577 | *psbE-petL* | LSC |
|  | c | (T)11agaataagttattc  t(A)10 | 36 | 69442 | 69477 | *psaJ-rpl33* | LSC |
|  | p1 | (T)15 | 15 | 69735 | 69749 | *psaJ-rpl33* | LSC |
|  | p1 | (A)13 | 13 | 70469 | 70481 | *rps18* | LSC |
|  | p4 | (TAAA)3 | 12 | 72507 | 72518 | *clpP** | LSC |
|  | p1 | (T)15 | 15 | 73650 | 73664 | *clpP** | LSC |
|  | p2 | (AT)6 | 12 | 74303 | 74314 | *clpP-psbB* | LSC |
|  | p1 | (T)10 | 10 | 77244 | 77253 | *petB** | LSC |
|  | p1 | (T)10 | 10 | 81650 | 81659 | *rps11-rpl36* | LSC |
|  | p1 | (T)12 | 12 | 83347 | 83358 | *rpl14-rpl16* | LSC |
|  | p1 | (T)12 | 12 | 84854 | 84865 | *rpl16 -rps3* | LSC |
|  | p1 | (T)10 | 10 | 85654 | 85663 | *rps3-rpl22* | LSC |
|  | p1 | (G)10 | 10 | 96444 | 96453 | *ycf2-trnL-CAA* | IRA |
|  | p1 | (T)11 | 11 | 101383 | 101393 | *rps12-trnV-GAC* | IRA |
|  | p1 | (A)10 | 10 | 120563 | 120572 | *ndhD-psaC* | SSC |
|  | p4 | (TTGA)3 | 12 | 121125 | 121136 | *ndhE* | SSC |
|  | c | (A)10tgaaccttag  tattatttattagtaaag  taattataaattattctttaat  caagagatttac(T)11 | 83 | 122513 | 122595 | *ndhG-ndhI* | SSC |
|  | p1 | (T)10 | 10 | 130107 | 130116 | *ycf1* | SSC |
|  | p1 | (A)13 | 13 | 130716 | 130728 | *ycf1* | SSC |
|  | p1 | (T)10 | 10 | 131100 | 131109 | *ycf1* | SSC |
|  | p1 | (A)11 | 11 | 143346 | 143356 | *trnV-GAC-rps12* | IRB |
|  | p1 | (C)10 | 10 | 148286 | 148295 | *trnL-CAA-ycf2* | IRB |
| *P.* *pedunculata* | p1 | (T)15 | 15 | 1614 | 1628 | *psbA-trnK-UUU* | LSC |
|  | p1 | (A)11 | 11 | 2929 | 2939 | *matK* | LSC |
|  | p1 | (T)11 | 11 | 3275 | 3285 | *matK* | LSC |
|  | p1 | (A)10 | 10 | 3766 | 3775 | *trnK-UUU** | LSC |
|  | p1 | (T)11 | 11 | 4038 | 4048 | *trnK-UUU** | LSC |
|  | p1 | (A)11 | 11 | 4881 | 4891 | *matK-rps16* | LSC |
|  | c | (AAAT)3taattaaattattgat  tttaagtacttttttcttattcttc  cccaaccg(A)16 | 77 | 5573 | 5649 | *rps16** | LSC |
|  | p5 | (TTTGA)3 | 15 | 5988 | 6002 | *rps16** | LSC |
|  | p1 | (A)15 | 15 | 6841 | 6855 | *rps16-trnQ-UUG* | LSC |
|  | p1 | (A)13 | 13 | 7693 | 7705 | *psbK-psbI* | LSC |
|  | p1 | (A)10 | 10 | 8454 | 8463 | *trnS-GCU-trnG-GCC* | LSC |
|  | p4 | (ATAA)4 | 16 | 8592 | 8607 | *trnS-GCU-trnG-GCC* | LSC |
|  | p1 | (T)10 | 10 | 9515 | 9524 | *trnG-GCC** | LSC |
|  | p1 | (T)13 | 13 | 9923 | 9935 | *trnG-GCC** | LSC |
|  | p1 | (T)10 | 10 | 11656 | 11665 | *atpF* | LSC |
|  | p1 | (A)10 | 10 | 12540 | 12549 | *atpF** | LSC |
|  | p2 | (AT)6 | 12 | 12735 | 12746 | *atpF** | LSC |
|  | p1 | (T)12 | 12 | 13117 | 13128 | *atpF-atpH* | LSC |
|  | p4 | (AAAG)3 | 12 | 13276 | 13287 | *atpF-atpH* | LSC |
|  | p1 | (T)11 | 11 | 13980 | 13990 | *atpH-atpI* | LSC |
|  | p1 | (T)10 | 10 | 14703 | 14712 | *atpH-atpI* | LSC |
|  | c | (T)10ctttg(A)16 | 31 | 16576 | 16606 | *rps2-rpoC2* | LSC |
|  | p1 | (T)11 | 11 | 18786 | 18796 | *rpoC2* | LSC |
|  | p1 | (T)10 | 10 | 26504 | 26513 | *rpoB* | LSC |
|  | p1 | (A)12 | 12 | 27584 | 27595 | *rpoB-trnC-GCA* | LSC |
|  | p1 | (T)10 | 10 | 28848 | 28857 | *trnC-GCA-petN* | LSC |
|  | p1 | (T)10 | 10 | 29442 | 29451 | *petN-psbM* | LSC |
|  | p4 | (AATA)3 | 12 | 29586 | 29597 | *petN-psbM* | LSC |
|  | p4 | (TTTA)3 | 12 | 30963 | 30974 | *psbM-trnD-GUC* | LSC |
|  | p4 | (TTTC)3 | 12 | 44912 | 44923 | *ycf3** | LSC |
|  | p1 | (T)10 | 10 | 48332 | 48341 | *trnT-UGU-trnL-UAA* | LSC |
|  | p1 | (A)12 | 12 | 48665 | 48676 | *trnT-UGU-trnL-UAA* | LSC |
|  | p1 | (T)10 | 10 | 56175 | 56184 | *atpB* | LSC |
|  | p1 | (T)10 | 10 | 58650 | 58659 | *rbcL-accD* | LSC |
|  | p1 | (T)15 | 15 | 58885 | 58899 | *rbcL-accD* | LSC |
|  | p1 | (T)10 | 10 | 61377 | 61386 | *psaI-ycf4* | LSC |
|  | p1 | (T)11 | 11 | 62599 | 62609 | *ycf4-cemA* | LSC |
|  | p1 | (A)10 | 10 | 65321 | 65330 | *petA-psbJ* | LSC |
|  | p1 | (G)10 | 10 | 66509 | 66518 | *psbE-petL* | LSC |
|  | p1 | (T)11 | 11 | 66952 | 66962 | *psbE-petL* | LSC |
|  | p1 | (A)18 | 18 | 67295 | 67312 | *psbE-petL* | LSC |
|  | c | (T)11agaataacttattct(A)11 | 37 | 69177 | 69213 | *psaJ-rpl33* | LSC |
|  | p1 | (T)16 | 16 | 69470 | 69485 | *psaJ-rpl33* | LSC |
|  | p1 | (A)13 | 13 | 70205 | 70217 | *rps18* | LSC |
|  | c | (T)10accttacttacacttac  atggaaaaaattt(TAAA)3 | 52 | 72186 | 72237 | *clpP** | LSC |
|  | p1 | (T)14 | 14 | 72372 | 72385 | *clpP** | LSC |
|  | p1 | (A)10 | 10 | 72569 | 72578 | *clpP** | LSC |
|  | p1 | (T)14 | 14 | 73401 | 73414 | *clpP** | LSC |
|  | p2 | (AT)6 | 12 | 74047 | 74058 | *clpP-psbB* | LSC |
|  | p1 | (T)19 | 19 | 76982 | 77000 | *petB** | LSC |
|  | p1 | (A)14 | 14 | 83023 | 83036 | *rpl16* | LSC |
|  | p1 | (A)10 | 10 | 83856 | 83865 | *rpl16** | LSC |
|  | p1 | (T)12 | 12 | 84611 | 84622 | *rpl16-rps3* | LSC |
|  | p1 | (T)10 | 10 | 85408 | 85417 | *rps3-rpl22* | LSC |
|  | p1 | (G)10 | 10 | 96117 | 96126 | *ycf15-trnL-CAA* | IRA |
|  | p4 | (TTGA)3 | 12 | 120629 | 120640 | *ndhE* | SSC |
|  | c | (A)10tgaaccttagtatta  tttattagtaaagtaatagtctt  agtaaagtaattataaattattctt  taatcaagagatttac(T)13 | 102 | 122018 | 122119 | *ndhG-ndhI* | SSC |
|  | p1 | (C)10 | 10 | 123999 | 124008 | *ndhA** | SSC |
|  | p1 | (T)10 | 10 | 129632 | 129641 | *ycf1* | SSC |
|  | p1 | (A)19 | 19 | 130256 | 130274 | *ycf1* | SSC |
|  | p1 | (T)10 | 10 | 130646 | 130655 | *ycf1* | SSC |
|  | p1 | (C)10 | 10 | 147822 | 147831 | *trnL-CAA-ycf15* | IRB |
| *P.* *persica* | p1 | (T)14 | 14 | 1591 | 1604 | *psbA-trnK-UUU* | LSC |
|  | p1 | (A)11 | 11 | 2905 | 2915 | *matK* | LSC |
|  | p1 | (T)11 | 11 | 3251 | 3261 | *matK* | LSC |
|  | p1 | (A)10 | 10 | 3742 | 3751 | *trnK-UUU** | LSC |
|  | p1 | (T)12 | 12 | 4014 | 4025 | *trnK-UUU** | LSC |
|  | c | (C)10tagaaacgtataa  gaagttttctcctcgtacg  gctcaagaaaattataaat  tatgtctatagtatagaattata  acatcaaacatatccata  (AAAT)3Taattaaattattgat  tttaagtacttttttcttattct  tccccaaccg(A)14 | 177 | 5457 | 5633 | *rps16** | LSC |
|  | p5 | (TTTGA)3 | 15 | 5977 | 5991 | *rps16** | LSC |
|  | c | (A)11gaatcacattctat  accaatattatacccttaaac  ggaagactgttcgaaaagaca  atctttaatttttatatactttattatg  atatag(AT)6 | 113 | 6827 | 6939 | *rps16-trnQ-UUG* | LSC |
|  | p1 | (A)10 | 10 | 8447 | 8456 | *trnS-GCU-trnG-GCC* | LSC |
|  | c | (T)12agatttctatgtc  aagaaatatattttgaatgac  ttgaataagaaacgctcttatt  acata(TTAAT)4 | 93 | 9637 | 9729 | *trnR-UCU-atpA* | LSC |
|  | p1 | (T)10 | 10 | 11612 | 11621 | *atpA -atpF* | LSC |
|  | p1 | (A)12 | 12 | 12497 | 12508 | *atpF** | LSC |
|  | p1 | (T)10 | 10 | 14670 | 14679 | *atpH-atpI* | LSC |
|  | c | (T)12ctttg(A)11 | 28 | 16548 | 16575 | *rps2-rpoC2* | LSC |
|  | p1 | (T)11 | 11 | 18755 | 18765 | *rpoC2* | LSC |
|  | p1 | (T)15 | 15 | 22877 | 22891 | *rpoC1** | LSC |
|  | p1 | (T)10 | 10 | 26483 | 26492 | *rpoB* | LSC |
|  | p1 | (A)11 | 11 | 27563 | 27573 | *rpoB-trnC-GCA* | LSC |
|  | p1 | (T)11 | 11 | 29253 | 29263 | *petN-psbM* | LSC |
|  | p2 | (TA)6 | 12 | 30150 | 30161 | *petN-psbM* | LSC |
|  | p4 | (TTTA)3 | 12 | 30781 | 30792 | *psbM-trnD-GUC* | LSC |
|  | p1 | (C)10 | 10 | 33810 | 33819 | *trnT-GGU-psbD* | LSC |
|  | p1 | (T)18 | 18 | 36743 | 36760 | *psbC-trnS-UGA* | LSC |
|  | p1 | (T)10 | 10 | 37239 | 37248 | *trnS-UGA-psbZ* | LSC |
|  | p1 | (T)10 | 10 | 44171 | 44180 | *ycf3** | LSC |
|  | p4 | (TTTC)3 | 12 | 44739 | 44750 | *ycf3** | LSC |
|  | p1 | (A)13 | 13 | 45714 | 45726 | *ycf3** | LSC |
|  | p2 | (AT)6 | 12 | 48038 | 48049 | *trnT-UGU-trnL-UAA* | LSC |
|  | p1 | (A)15 | 15 | 48492 | 48506 | *trnT-UGU-trnL-UAA* | LSC |
|  | p1 | (T)10 | 10 | 51949 | 51958 | *ndhK-ndhC* | LSC |
|  | p1 | (T)10 | 10 | 56026 | 56035 | *atpB* | LSC |
|  | p1 | (T)16 | 16 | 58725 | 58740 | *rbcL -accD* | LSC |
|  | p1 | (C)11 | 11 | 60639 | 60649 | *accD-psaI* | LSC |
|  | p1 | (T)13 | 13 | 61220 | 61232 | *psaI-ycf4* | LSC |
|  | p1 | (T)12 | 12 | 62326 | 62337 | *ycf4-cemA* | LSC |
|  | p1 | (T)11 | 11 | 62449 | 62459 | *ycf4-cemA* | LSC |
|  | p1 | (A)10 | 10 | 62588 | 62597 | *ycf4-cemA* | LSC |
|  | p1 | (A)12 | 12 | 65165 | 65176 | *petA -psbJ* | LSC |
|  | p1 | (G)10 | 10 | 66324 | 66333 | *psbE-petL* | LSC |
|  | p1 | (A)12 | 12 | 67119 | 67130 | *psbE-petL* | LSC |
|  | c | (T)11agaataagttattct  (A)13 | 39 | 69005 | 69043 | *psaJ -rpl33* | LSC |
|  | p1 | (T)15 | 15 | 69301 | 69315 | *psaJ -rpl33* | LSC |
|  | p1 | (A)13 | 13 | 70035 | 70047 | *rps18* | LSC |
|  | p1 | (A)12 | 12 | 70738 | 70749 | *rpl20-rps12* | LSC |
|  | p4 | (AAAT)3 | 12 | 72059 | 72070 | *clpP ** | LSC |
|  | p1 | (A)11 | 11 | 72859 | 72869 | *clpP ** | LSC |
|  | p1 | (A)11 | 11 | 72991 | 73001 | *clpP ** | LSC |
|  | p1 | (T)17 | 17 | 73207 | 73223 | *clpP ** | LSC |
|  | p2 | (AT)6 | 12 | 73862 | 73873 | *clpP-psbB* | LSC |
|  | p1 | (T)10 | 10 | 76798 | 76807 | *petB** | LSC |
|  | p1 | (T)11 | 11 | 84415 | 84425 | *rpl16 -rps3* | LSC |
|  | p1 | (T)10 | 10 | 85211 | 85220 | *rps3-rpl22* | LSC |
|  | p1 | (G)10 | 10 | 95920 | 95929 | *ycf2 -trnL-CAA* | IRA |
|  | p1 | (A)10 | 10 | 112256 | 112265 | *ycf1* | SSC |
|  | p1 | (T)10 | 10 | 115324 | 115333 | *ndhF -rpl32* | SSC |
|  | p4 | (TTGA)3 | 12 | 120488 | 120499 | *ndhE* | SSC |
|  | p1 | (A)10 | 10 | 120853 | 120862 | *ndhE -ndhG* | SSC |
|  | p1 | (T)10 | 10 | 129489 | 129498 | *ycf1* | SSC |
|  | p1 | (A)13 | 13 | 130098 | 130110 | *ycf1* | SSC |
|  | p1 | (T)10 | 10 | 130482 | 130491 | *ycf1* | SSC |
|  | p1 | (C)10 | 10 | 147658 | 147667 | *trnL-CAA-ycf2* | IRB |
| *P.* *tangutica* | p1 | (T)17 | 17 | 1693 | 1709 | *psbA-trnK-UUU* | LSC |
|  | p1 | (A)11 | 11 | 3010 | 3020 | *matK* | LSC |
|  | p1 | (T)11 | 11 | 3356 | 3366 | *matK* | LSC |
|  | p1 | (A)10 | 10 | 3847 | 3856 | *trnK-UUU ** | LSC |
|  | p1 | (T)11 | 11 | 4119 | 4129 | *trnK-UUU ** | LSC |
|  | p1 | (A)10 | 10 | 4962 | 4971 | *trnK-UUU -rps16* | LSC |
|  | c | (AAAT)3taattaaattat  tgattttaagtacttttttcttatt  cttccccaaccg(A)18 | 79 | 5652 | 5730 | *rps16** | LSC |
|  | p5 | (TTTGA)3 | 15 | 6069 | 6083 | *rps16** | LSC |
|  | p1 | (A)14 | 14 | 6923 | 6936 | *rps16-trnQ-UUG* | LSC |
|  | p1 | (A)14 | 14 | 7774 | 7787 | *psbK-psbI* | LSC |
|  | p1 | (A)11 | 11 | 8536 | 8546 | *trnS-GCU-trnG-GCC* | LSC |
|  | p4 | (ATAA)4 | 16 | 8674 | 8689 | *trnS-GCU-trnG-GCC* | LSC |
|  | p1 | (T)13 | 13 | 10004 | 10016 | *trnR-UCU-atpA* | LSC |
|  | p1 | (T)10 | 10 | 11717 | 11726 | *atpA-atpF* | LSC |
|  | p1 | (A)10 | 10 | 12601 | 12610 | *atpF** | LSC |
|  | p2 | (AT)6 | 12 | 12796 | 12807 | *atpF** | LSC |
|  | p1 | (T)12 | 12 | 13178 | 13189 | *atpF-atpH* | LSC |
|  | p4 | (AAAG)3 | 12 | 13337 | 13348 | *atpF-atpH* | LSC |
|  | p1 | (T)12 | 12 | 14041 | 14052 | *atpH-atpI* | LSC |
|  | p1 | (T)10 | 10 | 14764 | 14773 | *atpH-atpI* | LSC |
|  | c | (T)10ctttg(A)15 | 30 | 16637 | 16666 | *rps2-rpoC2* | LSC |
|  | p1 | (T)11 | 11 | 18846 | 18856 | *rpoC2* | LSC |
|  | p1 | (T)10 | 10 | 26562 | 26571 | *rpoB* | LSC |
|  | p1 | (A)11 | 11 | 27642 | 27652 | *rpoB -trnC-GCA* | LSC |
|  | p1 | (T)10 | 10 | 29498 | 29507 | *petN-psbM* | LSC |
|  | p4 | (AATA)3 | 12 | 29642 | 29653 | *petN-psbM* | LSC |
|  | p4 | (TTTA)3 | 12 | 31014 | 31025 | *psbM -trnD-GUC* | LSC |
|  | p1 | (T)10 | 10 | 36965 | 36974 | *psbC -trnS-UGA* | LSC |
|  | p4 | (TTTC)3 | 12 | 44954 | 44965 | *ycf3** | LSC |
|  | p1 | (A)13 | 13 | 48707 | 48719 | *trnT-UGU-trnL-UAA* | LSC |
|  | p2 | (TA)7 | 14 | 50545 | 50558 | *trnF-GAA -ndhJ* | LSC |
|  | p1 | (T)10 | 10 | 56222 | 56231 | *atpB* | LSC |
|  | p1 | (T)10 | 10 | 58697 | 58706 | *rbcL-accD* | LSC |
|  | p1 | (T)14 | 14 | 58926 | 58939 | *rbcL-accD* | LSC |
|  | p1 | (C)12 | 12 | 60838 | 60849 | *accD-psaI* | LSC |
|  | p1 | (T)10 | 10 | 61420 | 61429 | *psaI-ycf4* | LSC |
|  | p1 | (T)11 | 11 | 62642 | 62652 | *ycf4 -cemA* | LSC |
|  | p1 | (A)12 | 12 | 65364 | 65375 | *petA -psbJ* | LSC |
|  | p1 | (G)13 | 13 | 66553 | 66565 | *psbE-petL* | LSC |
|  | p1 | (T)11 | 11 | 66999 | 67009 | *psbE-petL* | LSC |
|  | p1 | (A)18 | 18 | 67342 | 67359 | *psbE-petL* | LSC |
|  | c | (T)11agaataacttattct(A)10 | 36 | 69223 | 69258 | *psaJ -rpl33* | LSC |
|  | p1 | (T)15 | 15 | 69516 | 69530 | *psaJ -rpl33* | LSC |
|  | p1 | (A)13 | 13 | 70250 | 70262 | *rps18* | LSC |
|  | c | (T)10accttacttacactta  catggaaaaaattt(TAAA)3 | 52 | 72231 | 72282 | *clpP** | LSC |
|  | p1 | (T)17 | 17 | 72417 | 72433 | *clpP** | LSC |
|  | p1 | (A)10 | 10 | 72617 | 72626 | *clpP** | LSC |
|  | p1 | (T)14 | 14 | 73449 | 73462 | *clpP** | LSC |
|  | p2 | (AT)6 | 12 | 74095 | 74106 | *clpP-psbB* | LSC |
|  | p1 | (T)17 | 17 | 77060 | 77076 | *petB** | LSC |
|  | c | (T)10gttttcaatgcaagg  gtctaaat(A)11 | 44 | 83067 | 83110 | *rpl14-rpl16* | LSC |
|  | p1 | (A)10 | 10 | 83923 | 83932 | *rpl16** | LSC |
|  | p1 | (T)19 | 19 | 84679 | 84697 | *rpl16 -rps3* | LSC |
|  | p1 | (T)10 | 10 | 85482 | 85491 | *rps3 -rpl22* | LSC |
|  | p1 | (G)10 | 10 | 96191 | 96200 | *ycf15-trnL-CAA* | IRA |
|  | p4 | (TTGA)3 | 12 | 120697 | 120708 | *ndhE* | SSC |
|  | c | (A)10tgaaccttagtat  tatttattagtaaagtaatagtc  ttagtaaagtaattataaattatt  ctttaatcaagagatttac(T)12 | 101 | 122086 | 122186 | *ndhG-ndhI* | SSC |
|  | p1 | (C)10 | 10 | 124066 | 124075 | *ndhA** | SSC |
|  | p1 | (T)10 | 10 | 129699 | 129708 | *rps15-ycf1* | SSC |
|  | p1 | (A)20 | 20 | 130323 | 130342 | *ycf1* | SSC |
|  | p1 | (T)10 | 10 | 130714 | 130723 | *ycf1* | SSC |
|  | p1 | (C)10 | 10 | 147900 | 147909 | *trnL-CAA -ycf15* | IRB |
| *P.* *triloba* | p1 | (A)11 | 11 | 75 | 85 | *rpl2-trnH-GUG* | LSC |
|  | p1 | (T)17 | 17 | 1901 | 1917 | *psbA -trnK-UUU* | LSC |
|  | p1 | (A)10 | 10 | 5170 | 5179 | *trnK-UUU-rps16* | LSC |
|  | p1 | (A)14 | 14 | 7132 | 7145 | *rps16 -trnQ-UUG* | LSC |
|  | p1 | (A)14 | 14 | 7983 | 7996 | *psbK-psbI* | LSC |
|  | p1 | (A)11 | 11 | 8745 | 8755 | *trnS-GCU-trnG-GCC* | LSC |
|  | p4 | (ATAA)4 | 16 | 8883 | 8898 | *trnS-GCU-trnG-GCC* | LSC |
|  | p1 | (T)13 | 13 | 10213 | 10225 | *trnR-UCU-atpA* | LSC |
|  | p1 | (T)10 | 10 | 11950 | 11959 | *atpA -atpF* | LSC |
|  | p1 | (T)12 | 12 | 13411 | 13422 | *atpF-atpH* | LSC |
|  | p4 | (AAAG)3 | 12 | 13570 | 13581 | *atpF-atpH* | LSC |
|  | p1 | (T)12 | 12 | 14274 | 14285 | *atpH-atpI* | LSC |
|  | p1 | (T)10 | 10 | 14997 | 15006 | *atpH-atpI* | LSC |
|  | c | (T)10ctttg(A)15 | 30 | 16870 | 16899 | *rps2-rpoC2* | LSC |
|  | p1 | (A)11 | 11 | 27877 | 27887 | *rpoB -trnC-GCA* | LSC |
|  | p1 | (T)10 | 10 | 29733 | 29742 | *petN-psbM* | LSC |
|  | p4 | (AATA)3 | 12 | 29877 | 29888 | *petN-psbM* | LSC |
|  | p4 | (TTTA)3 | 12 | 31249 | 31260 | *psbM-trnD-GUC* | LSC |
|  | p1 | (T)10 | 10 | 37200 | 37209 | *psbC -trnS-UGA* | LSC |
|  | p1 | (A)13 | 13 | 48941 | 48953 | *trnT-UGU-trnL-UAA* | LSC |
|  | p2 | (TA)7 | 14 | 50779 | 50792 | *trnF-GAA -ndhJ* | LSC |
|  | p1 | (T)10 | 10 | 58908 | 58917 | *rbcL-accD* | LSC |
|  | p1 | (T)14 | 14 | 59138 | 59151 | *rbcL-accD* | LSC |
|  | p1 | (C)12 | 12 | 61050 | 61061 | *accD-psaI* | LSC |
|  | p1 | (T)10 | 10 | 61632 | 61641 | *psaI -ycf4* | LSC |
|  | p1 | (T)11 | 11 | 62854 | 62864 | *ycf4-cemA* | LSC |
|  | p1 | (A)12 | 12 | 65576 | 65587 | *petA-psbJ* | LSC |
|  | p1 | (G)13 | 13 | 66765 | 66777 | *psbE-petL* | LSC |
|  | p1 | (T)11 | 11 | 67211 | 67221 | *psbE-petL* | LSC |
|  | p1 | (A)18 | 18 | 67554 | 67571 | *psbE-petL* | LSC |
|  | c | (T)11agaataacttattct(A)10 | 36 | 69435 | 69470 | *psaJ -rpl33* | LSC |
|  | p1 | (T)15 | 15 | 69727 | 69741 | *psaJ -rpl33* | LSC |
|  | c | (T)10gttttcaatgcaagggtc  taaat(A)11 | 44 | 83271 | 83314 | *rpl14-rpl16* | LSC |
|  | p1 | (T)19 | 19 | 84883 | 84901 | *rpl16-rps3* | LSC |
|  | p1 | (T)10 | 10 | 85687 | 85696 | *rps3-rpl22* | LSC |
|  | p1 | (G)10 | 10 | 96396 | 96405 | *ycf15 -trnL-CAA* | IRA |
|  | c | (A)10tgaaccttagtattatttat  tagtaaagtaatagtcttagtaa  agtaattataaattattctttaatc  aagagatttac(T)12 | 101 | 122291 | 122391 | *ndhI-ndhA* | SSC |
|  | p1 | (T)10 | 10 | 129904 | 129913 | *rps15-ycf1* | SSC |
|  | p1 | (C)10 | 10 | 148105 | 148114 | *trnL-CAA-ycf15* | IRB |
| *P. tenella* | p1 | (T)15 | 15 | 1944 | 1958 | *psbA-trnK-UUU* | LSC |
|  | p1 | (A)11 | 11 | 3258 | 3268 | *matK* | LSC |
|  | p1 | (T)11 | 11 | 3604 | 3614 | *matK* | LSC |
|  | p1 | (T)10 | 10 | 4366 | 4375 | *trnK-UUU** | LSC |
|  | c | (AAAT)3taattaaatta  ttgattttaagtacttttttctt  attcttccccaaccg(A)13 | 74 | 5903 | 5976 | *rps16** | LSC |
|  | p5 | (TTTGA)4 | 20 | 6315 | 6334 | *rps16** | LSC |
|  | p1 | (A)14 | 14 | 7173 | 7186 | *rps16-trnQ-UUG* | LSC |
|  | c | (A)12ttttcatttcgatttct  atgattatcgaatcaaatgat  atcgaatcagagtgtcatttctta  tcttaattttttatatttc(TTAT)3  attccttttatttcaatttcaattaaa  gtaaat(ATAA)4 | 154 | 8767 | 8920 | *trnS-GCU-trnG-GCC* | LSC |
|  | p1 | (T)10 | 10 | 9826 | 9835 | *trnG-GCC** | LSC |
|  | p1 | (T)11 | 11 | 10234 | 10244 | *trnR-UCU-atpA* | LSC |
|  | p1 | (A)11 | 11 | 12909 | 12919 | *atpF** | LSC |
|  | p2 | (AT)6 | 12 | 13105 | 13116 | *atpF** | LSC |
|  | p5 | (TTTAT)3 | 15 | 14986 | 15000 | *atpH-atpI* | LSC |
|  | p1 | (T)11 | 11 | 15110 | 15120 | *atpH-atpI* | LSC |
|  | c | (T)12ctttg(A)12 | 29 | 16984 | 17012 | *rps2-rpoC2* | LSC |
|  | p1 | (T)11 | 11 | 19203 | 19213 | *rpoC2** | LSC |
|  | p1 | (T)10 | 10 | 26914 | 26923 | *rpoB* | LSC |
|  | p1 | (T)11 | 11 | 27559 | 27569 | *rpoB-trnC-GCA* | LSC |
|  | p1 | (A)11 | 11 | 27996 | 28006 | *rpoB-trnC-GCA* | LSC |
|  | p1 | (T)11 | 11 | 29860 | 29870 | *petN-psbM* | LSC |
|  | p4 | (TTTA)3 | 12 | 31392 | 31403 | *psbM-trnD-GUC* | LSC |
|  | p1 | (T)11 | 11 | 37351 | 37361 | *psbC-trnS-UGA* | LSC |
|  | p1 | (T)10 | 10 | 37840 | 37849 | *trnS-UGA -psbZ* | LSC |
|  | p4 | (TTTC)3 | 12 | 45371 | 45382 | *psaA-trnS-GGA* | LSC |
|  | p2 | (AT)6 | 12 | 48670 | 48681 | *trnT-UGU-trnL-UAA* | LSC |
|  | p1 | (A)16 | 16 | 49131 | 49146 | *trnT-UGU-trnL-UAA* | LSC |
|  | p2 | (TA)7 | 14 | 50982 | 50995 | *trnF-GAA-ndhJ* | LSC |
|  | p1 | (T)11 | 11 | 51265 | 51275 | *trnF-GAA-ndhJ* | LSC |
|  | p1 | (T)10 | 10 | 56681 | 56690 | *atpB* | LSC |
|  | p1 | (T)10 | 10 | 59149 | 59158 | *rbcL-accD* | LSC |
|  | p1 | (T)13 | 13 | 59375 | 59387 | *rbcL-accD* | LSC |
|  | p1 | (C)17 | 17 | 61286 | 61302 | *accD-psaI* | LSC |
|  | p1 | (T)11 | 11 | 61890 | 61900 | *psaI-ycf4* | LSC |
|  | p1 | (T)11 | 11 | 63117 | 63127 | *ycf4 -cemA* | LSC |
|  | p1 | (A)11 | 11 | 65849 | 65859 | *petA-psbJ* | LSC |
|  | p1 | (G)11 | 11 | 67036 | 67046 | *psbE-petL* | LSC |
|  | p1 | (T)10 | 10 | 67479 | 67488 | *psbE-petL* | LSC |
|  | p1 | (A)16 | 16 | 67821 | 67836 | *psbE-petL* | LSC |
|  | c | (T)12agaataacttattct(A)10 | 37 | 69702 | 69738 | *psaJ -rpl33* | LSC |
|  | p1 | (T)16 | 16 | 69997 | 70012 | *psaJ -rpl33* | LSC |
|  | p1 | (A)13 | 13 | 70732 | 70744 | *rps18* | LSC |
|  | p4 | (TAAA)3 | 12 | 72752 | 72763 | *clpP** | LSC |
|  | p1 | (T)11 | 11 | 73953 | 73963 | *clpP** | LSC |
|  | p2 | (AT)6 | 12 | 74602 | 74613 | *clpP-psbB* | LSC |
|  | p1 | (T)10 | 10 | 77537 | 77546 | *psbH-petD* | LSC |
|  | p5 | (GATCC)3 | 15 | 79136 | 79150 | *petD** | LSC |
|  | c | (T)10gttttcaatgcaag  ggtctaaaaaaaaagaaata  ttatttgttcaaaacaaaaaaa  cctgcaa(T)10 | 83 | 83558 | 83640 | *rpl14 -rpl16* | LSC |
|  | p1 | (A)11 | 11 | 84395 | 84405 | *rpl16** | LSC |
|  | p1 | (T)18 | 18 | 85135 | 85152 | *rpl16-rps3* | LSC |
|  | p1 | (T)10 | 10 | 85938 | 85947 | *rps3-rpl22* | LSC |
|  | p1 | (G)10 | 10 | 96662 | 96671 | *ycf15-trnL-CAA* | IRA |
|  | p1 | (A)10 | 10 | 115254 | 115263 | *ndhF-rpl32* | SSC |
|  | p1 | (A)10 | 10 | 120654 | 120663 | *ndhD- psaC* | SSC |
|  | p4 | (TTGA)3 | 12 | 121216 | 121227 | *ndhE* | SSC |
|  | p1 | (T)15 | 15 | 122692 | 122706 | *ndhI* | SSC |
|  | p1 | (T)10 | 10 | 130217 | 130226 | *ycf1* | SSC |
|  | p1 | (A)13 | 13 | 130826 | 130838 | *ycf1* | SSC |
|  | p1 | (T)10 | 10 | 131210 | 131219 | *ycf1* | SSC |
|  | p1 | (C)10 | 10 | 148386 | 148395 | *trnL-CAA -ycf15* | IRB |

Note: * Indicates that the repeat sequence is in the intron

**Table S4 List of species accessions numbers (GenBank) were used in phylogenetic analysis.**

|  | Species | Accession number |
| --- | --- | --- |
| *Amygdalus* | *Prunus dulcis* | MT019559 |
|  | *Prunus davidiana* var. *potaninii* Rehd. | MT019558 |
|  | *Prunus davidiana* | NC039735 |
|  | *Prunus ferganensis* | MK798146 |
|  | *Prunus kansuensis* | NC023956 |
|  | *Prunus mira* | MK798147 |
|  | *Prunus mongolica* | KY073235 |
|  | *Prunus pedunculate* | MG869261 |
|  | *Prunus persica* | HQ336405 |
|  | *Prunus tangutica* | MK780039 |
|  | *Prunus triloba* | MK790138 |
|  | *Prunus tenella* | MK764428 |
| Rosaceae | *Prunus zippeliana* | NC_043926 |
|  | *Amelanchier obovalis* | MK920296 |
|  | *Chaenomeles japonica* | NC_035566 |
|  | *Cotoneaster submultiflorus* | MK920286 |
|  | *Crataegus kansuensis* | NC_039374 |
|  | *Cydonia oblonga* | KX499857 |
|  | *Dichotomanthes tristaniicarpa* | MN577869 |
|  | *Docynia delavayi* | KX499860 |
|  | *Eriobotrya japonica* | NC_034639 |
|  | *Hesperomeles pernettyoides* | MN068273 |
|  | *Heteromeles arbutifolia* | MK920281 |
|  | *Malus prattii* | MH929090 |
|  | *Mespilus germanica* | MK920295 |
|  | *Micromeles folgneri* | MK161058 |
|  | *Photinia prunifolia* | MK920279 |
|  | *Pyrus pyrifolia* | NC_015996 |
|  | *Rhaphiolepis major* | MN577887 |
|  | *Sorbus torminalis* | NC_033975 |
|  | *Stranvaesia nussia* | MK920284 |
|  | *Vauquelinia californica* | MN068269 |
|  | *Bencomia exstipulata* | MG682353 |
|  | *iphora fruticosa* | NC_036423 |
|  | *Fragaria pentaphylla* | NC_034347 |
|  | *Geum rupestre* | NC_037392 |
|  | *Hagenia abyssinica* | KX008604 |
|  | *Potentilla hebiichigo* | MK301251 |
|  | *Rosa banksiae* | NC_042194 |
|  | *Rubus hybrid* | MH992399 |
|  | *Sanguisorba officinalis* | NC_044694 |
|  | *Pentactina rupicola* | NC_016921 |
| Outgroups | *Prunus discoidea* | MN158647 |
|  | *Prunus maximowiczii* | NC_026981 |
|  | *Prunus pseudocerasus* | NC_030599 |
